# Educational attainment, structural brain reserve and Alzheimer’s disease: a Mendelian randomization analysis

**DOI:** 10.1093/brain/awac392

**Published:** 2022-10-31

**Authors:** Aida Seyedsalehi, Varun Warrier, Richard A I Bethlehem, Benjamin I Perry, Stephen Burgess, Graham K Murray

**Affiliations:** Department of Public Health and Primary Care, University of Cambridge, Cambridge CB2 0SR, UK; Department of Psychiatry, Warneford Hospital, University of Oxford, Oxford OX3 7JX, UK; Autism Research Centre, Department of Psychiatry, University of Cambridge, Cambridge CB2 8AH, UK; Autism Research Centre, Department of Psychiatry, University of Cambridge, Cambridge CB2 8AH, UK; Brain Mapping Unit, Department of Psychiatry, University of Cambridge, Cambridge CB2 0SZ, UK; Department of Psychiatry, University of Cambridge, Cambridge CB2 8AH, UK; CAMEO, Cambridgeshire and Peterborough NHS Foundation Trust, Cambridge CB4 1PX, UK; MRC Biostatistics Unit, University of Cambridge, Cambridge CB2 0SR, UK; Cardiovascular Epidemiology Unit, Department of Public Health and Primary Care, University of Cambridge, Cambridge CB2 0BB, UK; Department of Psychiatry, University of Cambridge, Cambridge CB2 8AH, UK; CAMEO, Cambridgeshire and Peterborough NHS Foundation Trust, Cambridge CB4 1PX, UK; Institute for Molecular Bioscience, University of Queensland, Brisbane 4072, Australia

**Keywords:** Mendelian randomization, Alzheimer’s disease, brain reserve, educational attainment, MRI

## Abstract

Higher educational attainment is observationally associated with lower risk of Alzheimer’s disease. However, the biological mechanisms underpinning this association remain unclear. The protective effect of education on Alzheimer’s disease may be mediated via increased brain reserve. We used two-sample Mendelian randomization to explore putative causal relationships between educational attainment, structural brain reserve as proxied by MRI phenotypes and Alzheimer’s disease.

Summary statistics were obtained from genome-wide association studies of educational attainment (*n* = 1 131 881), late-onset Alzheimer’s disease (35 274 cases, 59 163 controls) and 15 measures of grey or white matter macro- or micro-structure derived from structural or diffusion MRI (*n*_max_ = 33 211). We conducted univariable Mendelian randomization analyses to investigate bidirectional associations between (i) educational attainment and Alzheimer’s disease; (ii) educational attainment and imaging-derived phenotypes; and (iii) imaging-derived phenotypes and Alzheimer’s disease. Multivariable Mendelian randomization was used to assess whether brain structure phenotypes mediated the effect of education on Alzheimer’s disease risk.

Genetically proxied educational attainment was inversely associated with Alzheimer’s disease (odds ratio per standard deviation increase in genetically predicted years of schooling = 0.70, 95% confidence interval 0.60, 0.80). There were positive associations between genetically predicted educational attainment and four cortical metrics (standard deviation units change in imaging phenotype per one standard deviation increase in genetically predicted years of schooling): surface area 0.30 (95% confidence interval 0.20, 0.40); volume 0.29 (95% confidence interval 0.20, 0.37); intrinsic curvature 0.18 (95% confidence interval 0.11, 0.25); local gyrification index 0.21 (95% confidence interval 0.11, 0.31)]; and inverse associations with cortical intracellular volume fraction [−0.09 (95% confidence interval −0.15, −0.03)] and white matter hyperintensities volume [−0.14 (95% confidence interval −0.23, −0.05)]. Genetically proxied levels of surface area, cortical volume and intrinsic curvature were positively associated with educational attainment [standard deviation units change in years of schooling per one standard deviation increase in respective genetically predicted imaging phenotype: 0.13 (95% confidence interval 0.10, 0.16); 0.15 (95% confidence interval 0.11, 0.19) and 0.12 (95% confidence interval 0.04, 0.19)]. We found no evidence of associations between genetically predicted imaging-derived phenotypes and Alzheimer’s disease. The inverse association of genetically predicted educational attainment with Alzheimer’s disease did not attenuate after adjusting for imaging-derived phenotypes in multivariable analyses.

Our results provide support for a protective causal effect of educational attainment on Alzheimer’s disease risk, as well as potential bidirectional causal relationships between education and brain macro- and micro-structure. However, we did not find evidence that these structural markers affect risk of Alzheimer’s disease. The protective effect of education on Alzheimer’s disease may be mediated via other measures of brain reserve not included in the present study, or by alternative mechanisms.

## Teaser

Seyedsalehi *et al.* provide evidence that education leads to changes in brain structure: greater brain volume, surface area and curvature. However, the protective effects of education against Alzheimer's disease do not appear to be mediated by these effects on brain structure.

## Introduction

Despite a large body of observational epidemiological evidence supporting education as a protective factor for Alzheimer’s disease,^1–8^ the biological mechanisms underpinning this association are not well-established. A potential mechanism by which higher educational attainment (EA) protects against risk of Alzheimer’s is through increasing or maintaining the underlying ‘brain reserve’ of the individual.^5^ Brain reserve refers to individual differences in the anatomical and structural characteristics of the brain that enable some individuals to preserve their cognitive and functional status despite neuropathology.^9–11^ The progression of amyloid-β and tau pathology in Alzheimer’s disease is associated with several structural alterations in the brain, including progressive cortical thinning,^12–15^ widespread grey matter atrophy in cortical and subcortical regions^16,17^ and damage to the integrity and organization of white matter tracts.^18–21^ Many of these structural characteristics can be measured *in vivo* using structural or diffusion MRI, and thus their pre-morbid levels may serve as a useful proxy for the structural basis of brain reserve capacity.^22,23^ Consistent with the brain reserve hypothesis, observational neuroimaging studies of cognitively intact older adults have provided evidence for an association of education with several MRI measures of brain macro- and micro-structure that are implicated in Alzheimer’s disease. For instance, higher education levels have been associated with increased whole-brain and regional grey matter volume,^24–26^ cortical thickness^27–31^ and increased surface areas of sub-regions of the hippocampus and amygdala that are vulnerable to Alzheimer’s disease pathology.^32^ Similar findings have been reported for the association between education levels and white matter micro-structure in cognitively intact elderly, with more highly educated individuals showing increased white matter tract integrity in several brain regions that are characteristic sites of Alzheimer’s pathology.^28,33^

Given the observed associations between education and structural markers of brain reserve, it is plausible to hypothesize that changes in brain structure may mediate the protective effect of education on Alzheimer’s disease risk, through determining the underlying brain reserve of the individual. However, interpreting these observational associations as evidence of causal relationships between education, brain structure and Alzheimer’s risk relies on several untestable and potentially implausible assumptions, including the absence of residual and/or unmeasured confounding and reverse causation.^34^ For instance, the association of education with brain structural metrics may be confounded by early-life factors that are common predictors of both EA and brain structure, such as childhood socioeconomic status,^35,36^ birthweight,^37–41^ childhood cognitive ability^42^ and maternal smoking during pregnancy.^37,43^ Confounding may also affect the relationship between MRI markers of structural brain reserve and Alzheimer’s risk. Several of the established risk factors for Alzheimer’s disease and other dementias (e.g. smoking, hypertension, diabetes, excessive alcohol consumption and physical inactivity) have been shown to be associated with various aspects of brain macro- and micro-structure in community-dwelling populations.^44–55^ Therefore, it may be that these factors, rather than poor pre-morbid structural brain health, underlie the increase in Alzheimer’s disease risk. In addition, even if the observed associations reflect causal relationships, the direction of causality (i.e. from the exposure to the outcome or vice versa) cannot be definitively established using observational methods.^34^ For instance, the observational association between education and brain structure could arise as a result of longer duration of education causing a change in brain structural metrics, or alternatively, individuals with better structural brain health (e.g. larger brain volumes or cortical thickness) seeking longer durations of education (or a combination of both). Reverse causation could also affect the association between MRI markers of brain reserve and Alzheimer’s disease. Specifically, while greater structural brain reserve (as proxied by MRI metrics) may confer protection against advancing neuropathology and risk of an Alzheimer’s diagnosis, it is also possible that changes in these structural markers occur in response to pre-clinical Alzheimer’s disease (e.g. loss of brain volume or cortical thickness prior to clinical manifestations of dementia).

Mendelian randomization (MR)^34,56–58^ can be a useful approach for assessing putative causal relationships between education, brain structure and Alzheimer’s disease, as it addresses some of the common limitations of classical epidemiological studies (e.g. confounding and reverse causation). By using genetic variants that are specifically associated with a putative exposure as instrumental variables, MR can be used to make inferences about the causal effect of an exposure on an outcome.^59,60^ Due to the random assortment of alleles at conception, the distribution of genetic variants that are associated with a particular exposure is largely independent of factors that confound exposure–outcome associations in conventional observational analyses.^61–63^ Therefore, estimates from MR are less affected by environmental confounders, and can provide more reliable insights into causal relationships between risk factors and disease outcomes than classical epidemiological studies. In addition, given that the genotype of an individual is determined at conception and cannot be modified by subsequent disease outcomes, the direction of causation will always be from the genetic variant to the trait of interest, eliminating the potential for reverse causation.^34^ Therefore, MR can be particularly useful for obtaining reliable causal inferences in retrospective settings (e.g. in case-control studies) where genetic variants are measured after the occurrence of the disease outcome.^64^ For instance, while MRI markers of structural brain reserve cannot be reliably measured in individuals with Alzheimer’s disease (as the disease is likely to distort their measurement in cases), genetic variants associated with brain structure cannot be modified by the disease event and can therefore be used as a reliable proxy for pre-morbid brain structure in individuals with Alzheimer’s disease.

**Figure 1:**
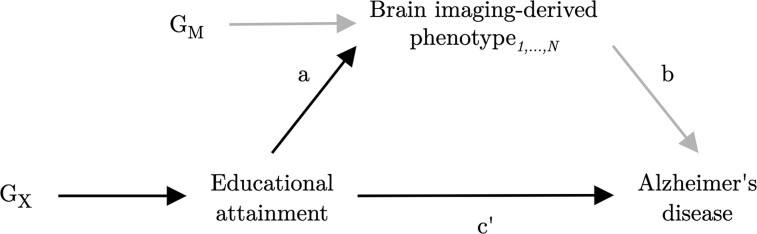
**Directed acyclic graph illustrating the putative causal relationships examined in this study.** Imaging-derived measures of brain structure are explored as potential mediators of the effect of EA (exposure) on Alzheimer’s disease (outcome). G_X_ represents the set of instrumental variables for EA. G_M_ represents the set of instrumental variables for brain imaging-derived phenotype*_1, …, N_*. Parameter *a* represents the direct causal effect of EA on imaging-derived phenotype*_1, …, N_*. Parameter *b* represents the direct causal effect of imaging-derived phenotype*_1, …, N_* on Alzheimer’s disease. Parameter *c’* represents the direct causal effect of EA on Alzheimer’s disease. Parameters *a* and *c’* are estimated using the set of variants G_X_, and parameter *b* is estimated using the set of variants G_M_. Confounding variables are omitted from the diagram.

In recent years, a growing number of MR investigations have found evidence for an inverse association of genetically predicted EA with Alzheimer’s disease risk.^65–69^ However, the mechanisms underlying the protective effect of education remain relatively unexplored. Recent advances in genotyping and multi-modal neuroimaging technologies have facilitated the collection of these data in large-scale prospective cohort studies,^70^ enabling the discovery of genetic variants associated with brain structure using considerably larger sample sizes than was previously possible.^71–74^ The present study aimed to expand on previous work by using MR to investigate putative causal relationships between EA, brain structure as measured by MRI and risk of Alzheimer’s disease. In addition, we aimed to explore whether there was any evidence for the hypothesis that brain structural alterations lie on the causal pathway from education to Alzheimer’s disease (i.e. the brain reserve hypothesis). We proxied structural brain reserve using imaging markers of grey and white matter macro- and micro-structure derived from structural or diffusion MRI (see Methods for the description and choice of these imaging-derived phenotypes). Our primary objectives were: (i) to replicate, using the latest genome-wide association study (GWAS) data, the previous MR findings supporting a protective causal effect of EA on Alzheimer’s disease risk; (ii) to assess whether EA has a causal effect on brain macro- and/or micro-structure; (iii) to assess whether brain macro- and/or micro-structure phenotypes causally affect risk of Alzheimer’s disease; and (iv) to assess whether the protective effect of EA on Alzheimer’s disease risk is mediated via changes to brain macro- and/or micro-structure, using multivariable MR (an extension to univariable MR which can be used to investigate mediating relationships).^75,76^ A directed acyclic graph illustrating the putative causal effects explored in this study is presented in Fig. 1. Although our primary hypotheses were in the directions stated before and shown in Fig. 1, we tested for a causal effect between each pair of variables in both directions (i.e. bidirectional or reciprocal MR), since this is a useful method for orientating the direction of causal effect (if any) between two variables.^77^

## Materials and methods

### Data sources

The present study is based on a two-sample MR analysis strategy, an extension of MR in which the effects of the genetic instruments on the exposure and the outcome are obtained from two separate datasets.^78,79^ To minimize the possibility of confounding due to population stratification, all data sources were restricted to individuals of European ancestry. The GWAS used to obtain the summary statistics for each phenotype are listed in Table 1. Detailed description of the data sources is available in Supplementary material.

**Table 1:** Data sources used to obtain summary statistics for MR analyses

Phenotype	GWAS	*n* (as exposure)	*n* (as outcome)
Educational attainment	Lee *et al.*^80^	1 131 881	766 345
Late-onset Alzheimer’s disease	Kunkle *et al.*^81^	35 274 cases 59 163 controls	21 982 cases 41 944 controls
**Cortical macro-structure**			
Surface area	In-house GWAS^82^	31 779	31 779
Volume		31 780	31 780
Cortical thickness		31 779	31 779
Local gyrification index		31 708	31 708
Mean curvature		31 779	31 779
Intrinsic curvature		31 748	31 748
**Cortical micro-structure**			
Fractional anisotropy (cortex)	In-house GWAS^82^	31 782	31 782
Mean diffusivity (cortex)		31 714	31 714
Intracellular volume fraction		31 768	31 768
Orientation dispersion index		31 797	31 797
**White matter micro-structure**			
Fractional anisotropy (white matter tracts)	Zhao *et al.*^74^	32 565	32 565
Mean diffusivity (white matter tracts)		32 343	32 343
**Subcortical volume**			
Volume of left hippocampus	Smith *et al.*^73^	33 211	33 211
Volume of right hippocampus		33 211	33 211
**White matter hyperintensities volume**			
Total volume of white matter hyperintensities	Smith *et al.*^73^	32 114	32 114

All genetic association estimates were taken from GWAS conducted on combined discovery and replication cohorts. Note that for some phenotypes there is a difference in sample size between the exposure and outcome datasets. This was either because the summary statistics for the full set of variants were not publicly available for one or more of the meta-analysed cohorts (e.g. 23andMe), or the full replication analysis on which the final list of genome-wide significant variants was based was only conducted on a subset of the variants meeting pre-specified significance thresholds at earlier discovery stages. Where this was the case, all analyses of the phenotype as an exposure were based on genome-wide significant variants from the largest analysed sample (to maximize power for selecting instruments). In the analyses of the phenotype as an outcome, summary statistics were obtained from the dataset for which association estimates were provided for the full set of variants.

**Figure 2:**
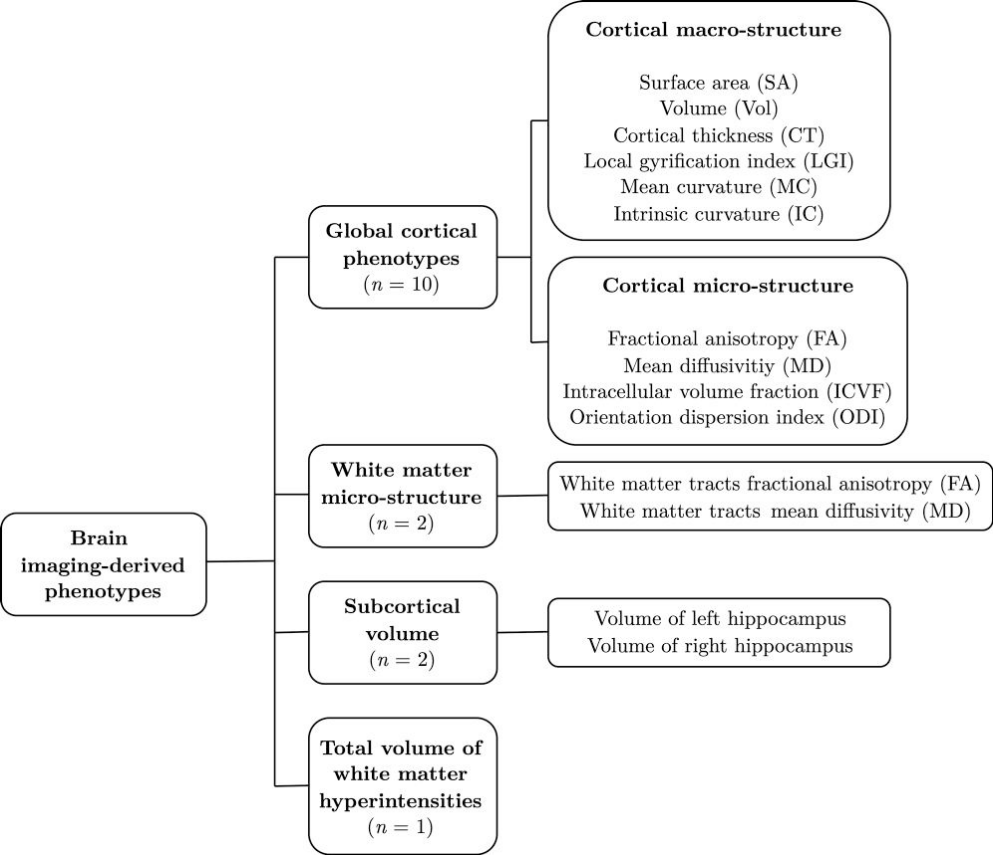
**Overview of the brain imaging-derived phenotypes investigated in this study.** Structural brain reserve was proxied by 15 brain imaging-derived phenotypes. These included 10 global cortical phenotypes (six macro-structural metrics derived from T_1_-weighted structural MRI and four micro-structural metrics derived from diffusion MRI), two white matter micro-structural phenotypes (measured at 21 major white matter tracts across the whole brain), bilateral hippocampal volumes and the total volume of white matter hyperintensities in the brain (derived from T_2_-weighted MRI).

Summary-level genetic association estimates with EA were obtained from a GWAS meta-analysis of ∼1.1 million individuals by Lee *et al*.^80^ EA was defined as the number of years of schooling (YOS) completed, measured at an age of at least 30 years. Genetic variant association estimates with Alzheimer’s disease were taken from the largest available GWAS meta-analysis of clinically diagnosed late-onset Alzheimer’s disease (onset age > 65 years), as conducted by the International Genomics of Alzheimer’s Project.^81^ Summary statistics for the imaging-derived phenotypes were obtained from three separate data sources based on the early-2020 release of combined genetic and multi-modal brain imaging data from the UK Biobank.^83^ Although several thousand imaging-derived phenotypes can be generated from UK Biobank MRI data, many are highly inter-correlated, and analysis of all imaging-derived phenotypes could lead to spurious results or challenges in controlling for multiple comparisons. Our strategy was to focus on 15 imaging-derived phenotypes (Fig. 2 and Supplementary material), most of which reflect global brain measures previously shown to be associated phenotypically with EA and/or Alzheimer’s disease.^12,14,16–19,24–28,31–33^ We also chose to study the total volume of white matter hyperintensities as a metric closely linked to age-related cognitive decline and thus a putative marker of structural brain health.^84–87^ A more detailed explanation of the rationale for selection of imaging-derived phenotypes is provided in the Supplementary material. To generate genetic variant association estimates with the whole-brain cortical macro-structure (surface area, volume, cortical thickness, intrinsic curvature, mean curvature, local gyrification index) and micro-structure phenotypes (mean diffusivity, fractional anisotropy, intracellular volume fraction and orientation dispersion index), we conducted an in-house GWAS^82^ using the latest UK Biobank data release (Supplementary material). Genetic variant association estimates with the white matter micro-structure phenotypes were obtained from a recently published GWAS of diffusion MRI data by Zhao *et al*.^74^ For this study, we used summary statistics for the global fractional anisotropy and mean diffusivity phenotypes, which were averaged across 21 predefined cerebral white matter tracts. For hippocampal volume and the total volume of white matter hyperintensities, we obtained genetic variant association estimates from a recently published GWAS of 3913 imaging-derived phenotypes by Smith *et al*.^73^ The cortical macro-/micro-structure and white matter micro-structure phenotypes were not corrected for intracranial volume (ICV) or total brain volume, as these were all global measures and would be highly correlated with whole-brain volume. However, for the localized phenotypes (hippocampal volume and the total volume of white matter hyperintensities), we used genetic association estimates that were corrected for ICV. All genetic association estimates were taken from GWAS conducted on the combined discovery and replication cohorts.

### Selection of genetic instruments and data harmonization

For each MR analysis, we selected all single nucleotide polymorphisms (SNPs) associated with the exposure of interest at a genome-wide significance threshold (*P* < 5 × 10^−8^) as instrumental variables. The resulting instruments were pruned to near-independence using a linkage disequilibrium (LD) threshold of *r*^2^ < 0.001 over 10 000 kilobase pairs. We used the LD reference panel for the European super-population in the 1000 Genomes Project reference dataset, which was restricted to bi-allelic SNPs with minor allele frequency >0.01. We extracted the following summary-level data from each exposure and outcome GWAS: SNP rs number, effect and other alleles, effect allele frequency, sample size, number of cases/controls (if applicable), standardized β-coefficients, standard errors and *P*-values. Where an instrument SNP was not available in the outcome dataset, we used LDlink^88^ to identify a proxy SNP in LD with the target SNP (*r*^2^ > 0.8). The exposure and outcome GWAS datasets were then harmonized to ensure that the genetic variant association estimates corresponded to the effect of the same allele. We used allele frequency information to infer the orientation of alleles in the exposure and outcome GWAS. Palindromic SNPs with minor allele frequency >0.42 were dropped from the analysis.^89^ Details of the SNPs used as instruments in each MR analysis (including proxies for SNPs not available in the outcome datasets) are available in the Supplementary material. For each analysis, we used the online power calculator by Burgess^90^ (https://sb452.shinyapps.io/power/) to estimate the minimum causal effect that we had 80% power to detect at a multiple-testing-corrected significance threshold (see the ‘Correction for multiple testing’ section later). Power calculations are presented in Supplementary Table 1.

### Statistical analyses

#### Bidirectional univariable Mendelian randomization

We performed univariable MR analyses to estimate each of the following total causal effects: (i) EA on Alzheimer’s disease; (ii) EA on each of the 15 imaging-derived phenotypes; and (iii) imaging-derived phenotypes on Alzheimer’s disease. To examine the direction of association and investigate the possibility of reverse causation, we additionally estimated each of these causal effects in the direction opposite to that initially hypothesized (i.e. bidirectional MR).^77^ As recommended by Burgess *et al.*,^91^ we used inverse-variance weighted (IVW) MR^92^ with multiplicative random-effects as the primary analysis method, as it provides the most efficient combination of the variant-specific ratio estimates^91–93^ and accounts for heterogeneity in the causal estimates obtained from individual variants.^91,94^

#### Correction for multiple testing

To account for the number of MR analyses performed to test for bidirectional effects of education on brain structure and brain structure on Alzheimer’s disease, we applied the multiple-testing correction approach described by Lord *et al*.^68^ Given the correlation structure between the various MRI metrics considered, applying a simple Bonferroni correction could be overly conservative. We therefore used a principal component analysis approach (https://github.com/hagax8/independent_tests) to estimate the number of independent hypotheses tested from the matrix of squared phenotypic correlations between the imaging-derived phenotypes (Supplementary material and Supplementary Fig. 1). We first calculated the number of principal components that explained 99.5% of the variance in the matrix as *n* = 13. The number of individual tests within the squared correlation matrix was then calculated as *T* = (*n × n −* n)/2 (=78), and the square root of *T* (=8.83) was used to establish the final number of individual imaging-derived phenotypes to correct for. Bidirectional associations between EA and imaging-derived phenotypes and between imaging-derived phenotypes and Alzheimer’s disease were therefore considered significant at a multiple-testing corrected threshold of *P* < 0.006 (=0.05/8.83). For testing bidirectional causal relationships between EA and Alzheimer’s disease, we set statistical significance at *P* < 0.05.

Note that the purpose of the multiple-testing correction was to control the family-wise error rate within each objective (i)–(iv), rather than across the total number of univariable MR analyses performed (i.e. 62). Controlling the family-wise error rate across all 62 tests would be appropriate if we were interested in the global null hypothesis (i.e. the intersection hypothesis of all null hypotheses of interest).^95^ Given that each of the objectives of interest were distinct (i.e. we were interested in detecting putative causal relationships between education and Alzheimer’s disease, education and brain structure, and brain structure and Alzheimer’s disease), we applied the multiple-testing correction to the number of hypotheses within each objective.

#### Sensitivity analyses

For each univariable MR analysis where a causal effect was detected using the IVW method, we used the following four methods to assess the robustness of that finding to potential violations of MR assumptions: the Contamination Mixture method,^96^ Weighted Median MR,^97^ MR-Egger^98^ and the MR pleiotropy residual sum and outlier (MR-PRESSO) method.^99^ These four robust methods were selected since they each produce a valid estimate of the causal effect of the exposure on the outcome under different assumptions (see Supplementary material for a description of the assumptions of each method).^100^

In addition to robust MR methods, we performed a range of other sensitivity analyses where a causal effect was detected using the IVW method. First, we assessed the presence of heterogeneity amongst the variant-specific causal estimates using Cochran’s *Q* statistic.^101,102^ Second, we generated funnel plots of the precision of the variant-specific causal estimates against the estimates themselves. The funnel plot is expected to be symmetrical about the IVW estimate, with more precise estimates having less variability.^103^ The presence of asymmetry in the funnel plot is suggestive of directional pleiotropy and bias in the overall causal estimate.^98,103^ Third, we carried out leave-one-out sensitivity analyses to identify influential data points amongst the instruments for each exposure, and to assess the reliance of the causal effect estimate on individual genetic variants.^104^ Finally, to orient the direction of causality between the exposure and the outcome, we used the Steiger directionality test,^105^ an extension to two-sample MR, which detects variants that have a stronger association with the outcome than with the exposure. Where there was evidence from the Steiger test that some genetic instruments had a stronger association with the outcome, we repeated the analysis excluding those variants.^91^

During the review process, we conducted additional sensitivity analyses using summary statistics from a recently published GWAS^106^ of longitudinal changes in brain structure across the lifespan (see Supplementary material and Supplementary Table 7 for details).

#### Multivariable Mendelian randomization to test for mediation

We performed multivariable MR analyses^75,76,107^ to investigate the potential mediating role of brain structure in the relationship between EA and Alzheimer’s disease. Multivariable MR allows the genetic variants associated with both the primary exposure and the secondary exposure (mediator) to be included as instruments in the analysis, and can be used to dissect the total causal effect of the exposure on the outcome into an indirect effect via the mediator, and a direct effect of the exposure on the outcome not via the mediator (i.e. via other causal pathways or other mediators; Supplementary material and Supplementary Fig. 2).^76,108^ While univariable MR estimates the total causal effect of the exposure on the outcome, multivariable MR estimates the direct effect of the exposure on the outcome, while adjusting for the mediator. The indirect (mediated) effect of the exposure on the outcome can then be estimated by subtracting the direct effect from the total effect.^76,108^ This broadly mirrors the difference-in-coefficients method used in traditional non-instrumental variable regression-based approaches to mediation.^109^

Using this approach, we estimated the direct causal effect of EA on Alzheimer’s disease risk, while adjusting for each of the 15 imaging-derived phenotypes in turn. A difference between the estimate of the total causal effect of EA on Alzheimer’s disease (from univariable MR) and the direct causal effect estimate (from the multivariable MR model including an imaging-derived phenotype) would indicate a mediating role of that brain structure phenotype on the causal pathway from education to Alzheimer’s disease.^107,108^ For each multivariable MR analysis, we selected all SNPs associated with the primary exposure (EA) or the mediator (imaging-derived phenotype of interest) as instrumental variables. The pooled set of SNPs were clumped to pairwise LD (*r*^2^ < 0.001) over 10 000 kilobase pairs, on the basis of the lowest *P*-value for association with any of the two traits. The extraction of summary-level data from the Alzheimer’s disease GWAS, harmonization of the exposure and outcome datasets, and identification of proxies for missing SNPs, followed the same procedure as univariable MR, as described before.

All statistical analyses were performed in R software (The R Foundation for Statistical Computing, Vienna, Austria).^110^ We used the ‘TwoSampleMR’ package^89^ (v.0.5.6) for data extraction and harmonization, the ‘ieugwasr’ package (https://mrcieu.github.io/ieugwasr/index.html) for clumping and the ‘LDlink’ package^88^ (v.1.1.2.9) for extracting LD proxies. All MR analyses were carried out using the ‘MendelianRandomization’^111^ (v.0.5.1) and ‘MRPRESSO’^99^ (v.1.0) packages.

### Ethical approval

The present study is a secondary analysis of publicly available data. Ethical approval was granted for each of the original GWAS studies, the details of which can be found in the respective publications.

### Data and code availability

Summary statistics from the in-house GWAS of cortical macro- and micro-structure are available at https://portal.ide-cam.org.uk. Individual-level imaging and genetic data used for the in-house GWAS analyses may be requested through the UK Biobank (https://www.ukbiobank.ac.uk/), and the code used in generating the imaging phenotypes is available on GitHub (https://github.com/ucam-department-of-psychiatry/UKB). Summary-level genetic association estimates with all other phenotypes were obtained from publicly available published GWAS, and can be accessed from the respective publications. R code for reproducing all MR analyses can be found on https://github.com/as2970/EA_brain_AD_MR.

## Results

### Inverse association of genetically predicted educational attainment with Alzheimer’s disease risk

**Figure 3:**
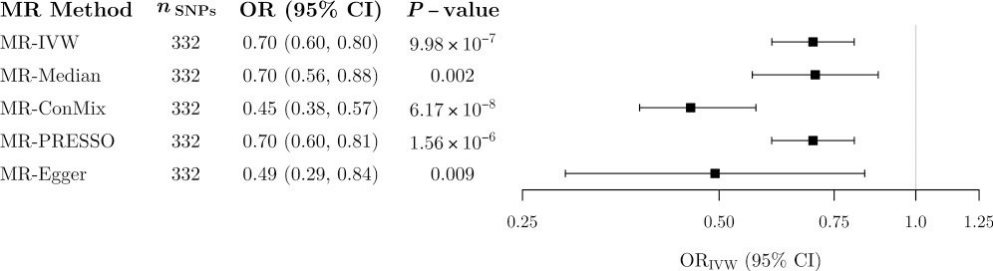
**MR estimates of the association between genetically proxied EA and Alzheimer’s disease.** Estimates represent odds ratios (95% CIs) for late-onset Alzheimer’s disease per 1 SD increase in genetically predicted YOS (∼4.2 years).

There was strong evidence for an inverse association between genetically proxied EA and Alzheimer’s disease across all MR methods (Fig. 3 and Supplementary Fig. 3). The results of the main IVW analysis suggested that each SD increase in genetically predicted YOS (∼4.2 years) was associated with a 30% reduction in odds of Alzheimer’s disease (OR = 0.70, 95% CI 0.60, 0.80). The weighted median and MR-PRESSO methods provided similar estimates to the IVW analysis, while larger inverse estimates were observed in the contamination mixture and MR-Egger analyses (Fig. 3).

Cochran’s *Q* statistic provided no evidence that there was more heterogeneity in the variant-specific causal estimates than expected due to chance (Supplementary Table 2). There was no evidence of directional pleiotropy using the MR-Egger intercept test (Supplementary Table 3), no evidence of departure from symmetry in the funnel plot (Supplementary Fig. 4) and no distortion in the leave-one-out plot (Supplementary Fig. 5). The Steiger directionality test indicated that 24 (7%) of the 332 education-associated SNPs explained more variance in Alzheimer’s disease than in EA. When these variants were excluded from the analysis, there was still strong evidence for an inverse association of genetically proxied education with Alzheimer’s disease (OR_IVW_ = 0.72, 95% CI 0.62, 0.83; Supplementary Table 4).

In the opposite direction, as expected, there was no association between genetically predicted Alzheimer’s disease and years of education. The IVW estimate (representing the SD change in YOS per doubling the odds of Alzheimer’s disease) was 0.00 (95% CI 0.00, 0.01, *P* = 0.145).

### Association of genetically predicted educational attainment with brain structure

**Figure 4:**
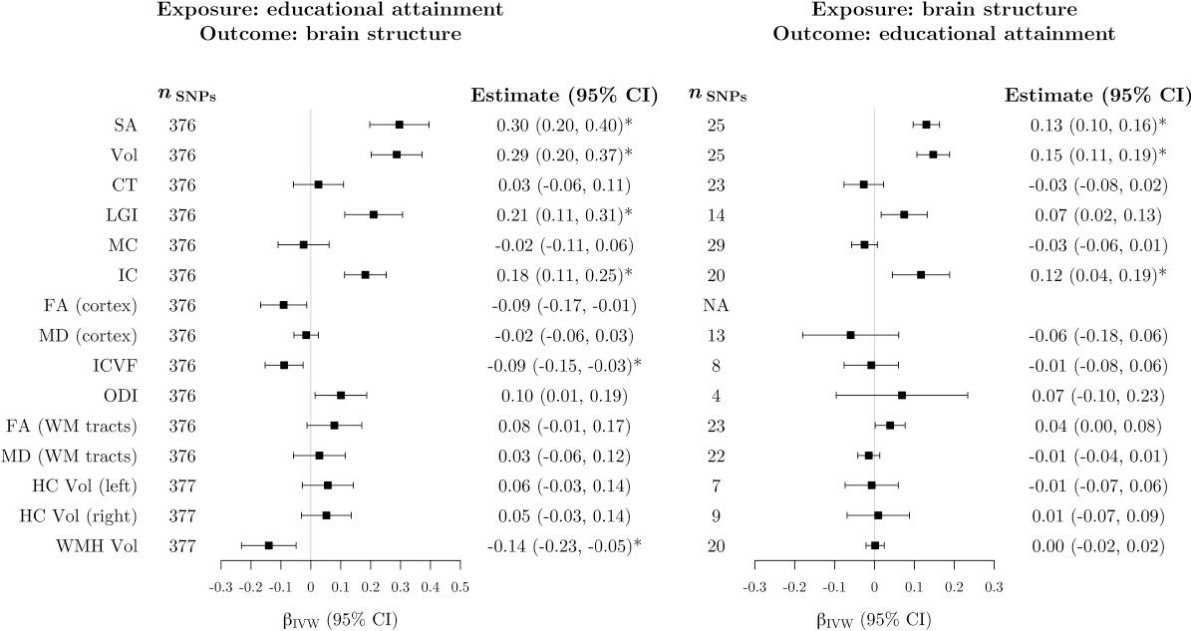
**MR estimates of bidirectional associations between EA and brain imaging-derived phenotypes.** *Left*: Estimates from IVW MR of the association between genetically proxied EA and imaging-derived brain structure phenotypes. Estimates represent the SD change in the imaging phenotype per 1 SD increase in genetically predicted YOS (∼4.2 years). *Right*: Estimates from IVW MR of the association between genetically proxied brain structure phenotypes and EA. Estimates represent the SD change in YOS per 1 SD increase in genetically predicted levels of each imaging-derived phenotype. Estimates marked with an asterisk were significant after correction for multiple testing. The SNPs column represents the number of SNPs remaining after clumping for independence and data harmonization. There were no significant clumps for the FA (cortex) phenotype after LD pruning. CT = cortical thickness; FA = fractional anisotropy; HC = hippocampus; IC = intrinsic curvature; ICVF = intracellular volume fraction; LGI = local gyrification index; MC = mean curvature; MD = mean diffusivity; ODI = orientation dispersion index; SA = surface area; Vol = volume; WM = white matter; WMH = white matter hyperintensities.

After correction for multiple testing, genetically proxied EA was positively associated with four measures of cortical macro-structure (Fig. 4 and Supplementary Fig. 6). In IVW analyses, each SD increase in genetically predicted YOS was associated with a 0.30 SD increase in surface area (95% CI 0.20, 0.40), 0.29 SD increase in cortical volume (95% CI 0.20, 0.37), 0.18 SD increase in intrinsic curvature (95% CI 0.11, 0.25) and 0.21 SD increase in local gyrification index (95% CI 0.11, 0.31). In addition, inverse associations were observed with intracellular volume fraction (β_IVW_ = −0.09, 95% CI −0.15, −0.03) and the total volume of white matter hyperintensities (β_IVW_ = −0.14, 95% CI −0.23, −0.05). The results of the pleiotropy robust methods were broadly consistent with the IVW analyses (Supplementary Fig. 6). For local gyrification index, intracellular volume fraction and white matter hyperintensities volume, confidence intervals around the MR-Egger estimate included the null. However, the point estimates were still in the same direction, and all other robust methods were in line with the IVW analysis.

All analyses demonstrated significant evidence of heterogeneity in the variant-specific causal estimates (Supplementary Table 2). The funnel plot for cortical surface area demonstrated a slight departure from symmetry, but all other funnel plots were symmetrical about the IVW estimate (Supplementary Figs 7–12). The MR-Egger intercept test did not show evidence of directional pleiotropy in any of the analyses (Supplementary Table 3), and there was no distortion in any of the leave-one-out plots (Supplementary Figs 13–18). The proportion of education-associated SNPs that explained more variance in the imaging phenotype than in EA ranged from 14% for intracellular volume fraction to 30% for white matter hyperintensities volume (Supplementary Table 4). In sensitivity analyses excluding the invalid variants from each analysis, all estimates were attenuated towards the null, and the associations with local gyrification index and white matter hyperintensities volume were no longer statistically significant (Supplementary Table 4).

### Association of genetically predicted brain structure with educational attainment

After correction for multiple testing, genetically predicted levels of three cortical macro-structure phenotypes (surface area, volume and intrinsic curvature) were positively associated with EA (Fig. 4 and Supplementary Fig. 19). The IVW estimates, representing the change in YOS (SD units) per 1 SD increase in genetically predicted levels of imaging-derived phenotypes were 0.13 (95% CI 0.10, 0.16) for surface area, 0.15 (95% CI 0.11, 0.19) for volume and 0.12 (95% CI 0.04, 0.19) for intrinsic curvature. For cortical surface area and volume, all robust methods provided similar estimates to the IVW analysis, but confidence intervals around the MR-Egger estimates were considerably wider than the other methods and included the null (Supplementary Fig. 19). The MR-Egger estimate of the association between genetically predicted intrinsic curvature and EA was negative, whereas all other estimates of this association were positive. However, the confidence interval for the MR-Egger estimate still overlapped with all other point estimates (Supplementary Fig. 19).

Using Cochran’s *Q* statistic, we found evidence of heterogeneity in all three analyses (Supplementary Table 2). However, the funnel plots showed little evidence of departure from symmetry (Supplementary Figs 20–22), and the MR-Egger intercept term did not significantly differ from zero in any analysis (Supplementary Table 3). In leave-one-out sensitivity analyses, the overall IVW estimates did not change on exclusion of any variant (Supplementary Figs 23–25). The Steiger directionality test did not identify any SNPs that explained more variance in EA than in the imaging phenotype for any analysis (Supplementary Table 4).

### Association of genetically predicted Alzheimer’s disease with brain structure

**Figure 5:**
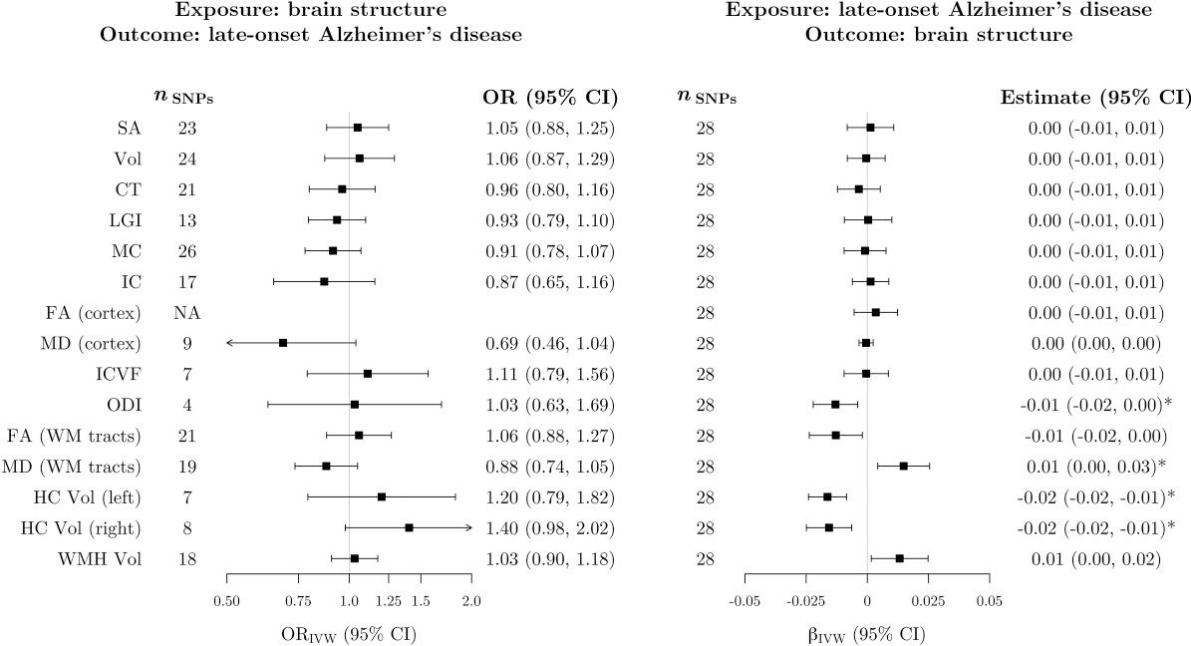
**MR estimates of bidirectional associations between brain imaging-derived phenotypes and Alzheimer’s disease.** *Left*: Estimates from IVW MR of the association between genetically proxied imaging-derived phenotypes and Alzheimer’s disease. Estimates represent the odds ratio for late-onset Alzheimer’s disease per SD increase in genetically predicted levels of each imaging-derived phenotype. *Right*: Estimates from IVW MR of the association between genetically proxied Alzheimer’s disease and brain structure phenotypes. Estimates represent the average change in each imaging-derived phenotype (SD units) per doubling (2-fold increase) in the odds of genetically predicted late-onset Alzheimer’s disease. Estimates marked with an asterisk were significant after correction for multiple testing. The SNPs column represents the number of SNPs remaining after clumping for independence and data harmonization. There were no significant clumps for the FA (cortex) phenotype after LD pruning. CT = cortical thickness; FA = fractional anisotropy; HC = hippocampus; IC = intrinsic curvature; ICVF = intracellular volume fraction; LGI = local gyrification index; MC = mean curvature; MD = mean diffusivity; ODI = orientation dispersion index; SA = surface area; Vol = volume; WM = white matter; WMH = white matter hyperintensities.

Using the primary IVW analysis method, we did not find evidence for an association between genetically predicted levels of any imaging-derived phenotype and Alzheimer’s disease (Fig. 5). However, genetically predicted Alzheimer’s disease was associated with four measures of brain structure (Fig. 5 and Supplementary Fig. 26), namely, reduced cortical orientation dispersion index [β_IVW_ = −0.02 (SD units per doubling the odds of genetically proxied Alzheimer’s disease), 95% CI −0.03, −0.01], increased white matter mean diffusivity (β_IVW_ = 0.02, 95% CI 0.01, 0.04) and reduced hippocampal volume in both hemispheres (left hemisphere: β_IVW_ = −0.02, 95% CI −0.03, −0.01; right hemisphere: β_IVW_ = −0.02, 95% CI −0.04, −0.01). For left and right hippocampal volume, all robust methods provided similar estimates to the IVW analysis (Supplementary Fig. 26). For orientation dispersion index, the 95% CI for the contamination mixture estimate included the null, but the point estimate was identical to the other methods (Supplementary Fig. 26). The contamination mixture and MR-PRESSO methods both suggested a null association of genetically predicted Alzheimer’s disease with white matter mean diffusivity (Supplementary Fig. 26).

We found evidence of heterogeneity in the variant-specific causal estimates for the association of genetically proxied Alzheimer’s disease with white matter mean diffusivity (Supplementary Table 2). In addition, there was evidence of potential horizontal pleiotropy in this analysis using the MR-Egger intercept test (Supplementary Table 3). There was little evidence of departure from symmetry in the funnel plots (Supplementary Figs 27–30). In leave-one-out sensitivity analyses, the association of genetically predicted Alzheimer’s disease with all four imaging-derived phenotypes appeared to be driven entirely by one genetic variant (rs12721046). In each case, exclusion of this variant from the analysis attenuated the IVW estimate towards the null (Supplementary Fig. 3^1^). To further investigate the reliance of the IVW estimates on this variant, we conducted *post hoc* single-SNP MR analyses and visualized the results using forest plots (Supplementary Fig. 3^2^). Although the rs12721046 variant did not have an outlying MR estimate for any imaging-derived phenotype, the precision of its estimates was unusually high, causing this variant to dominate the wider polygenic signal in each case (Supplementary Fig. 3^2^). In Steiger filtering sensitivity analyses, all instruments explained more variance in Alzheimer’s disease than in the imaging phenotypes (Supplementary Table 4).

### Mediation analysis

In multivariable MR analyses, there was little change in the association of genetically predicted YOS with Alzheimer’s disease after adjusting for genetically predicted levels of each of the 15 imaging-derived phenotypes (Table 2).

**Table 2:** MR estimates of the association between genetically proxied EA and Alzheimer’s disease risk, estimated with no adjustment and after adjustment for genetically proxied levels of brain imaging-derived phenotypes

Adjustment	OR_IVW_(95% CI)	*P*-value
None (univariable analysis)	0.70 (0.60, 0.80)	9.98 × 10^−7^
**Cortical macro-structure**		
Surface area	0.71 (0.62, 0.82)	3.68 × 10^−6^
Volume	0.72 (0.62, 0.83)	6.12 × 10^−6^
Cortical thickness	0.69 (0.60, 0.80)	3.62 × 10^−7^
Local gyrification index	0.71 (0.61, 0.81)	1.39 × 10^−6^
Mean curvature	0.69 (0.60, 0.79)	1.80 × 10^−7^
Intrinsic curvature	0.71 (0.62, 0.82)	2.74 × 10^−6^
**Cortical micro-structure**		
Mean diffusivity (cortex)	0.70 (0.61, 0.81)	9.35 × 10^−7^
Intracellular volume fraction	0.71 (0.62, 0.82)	2.72 × 10^−6^
Orientation dispersion index	0.70 (0.61, 0.81)	8.65 × 10^−7^
**White matter micro-structure**		
Fractional anisotropy (white matter tracts)	0.71 (0.61, 0.81)	1.53 × 10^−6^
Mean diffusivity (white matter tracts)	0.71 (0.61, 0.82)	2.42 × 10^−6^
**Subcortical volume**		
Volume of left hippocampus	0.70 (0.61, 0.80)	5.66 × 10^−7^
Volume of right hippocampus	0.70 (0.61, 0.81)	7.74 × 10^−7^
**White matter hyperintensities volume**		
Total volume of white matter hyperintensities	0.71 (0.61, 0.81)	1.43 × 10^−6^

OR (95% CIs) for Alzheimer’s disease are estimated per 1 SD increase in genetically predicted YOS (∼4.2 years), estimated with no adjustment (univariable IVW analysis) and after adjustment for genetically predicted levels of each brain imaging-derived phenotype in turn (multivariable IVW analyses).

## Discussion

We conducted bidirectional univariable and multivariable MR analyses using large genome-wide association datasets to examine putative causal relationships between EA, brain macro- and micro-structure as measured by MRI and risk of Alzheimer’s disease. We found strong evidence in support of a protective causal effect of EA on Alzheimer’s disease risk, with no evidence of a causal effect in the reverse direction. There was also evidence of bidirectional causal relationships between education and several measures of cortical macro- and micro-structure. However, we did not find any evidence in support of a causal effect of the brain structure phenotypes examined in this study on risk of Alzheimer’s disease. Additionally, there was no evidence that the protective effect of EA on Alzheimer’s disease risk was mediated via changes to these structural markers. In the opposite direction, we found evidence for an association of genetically predicted Alzheimer’s disease with hippocampal volume, as well as cortical and white matter micro-structure. However, these effects appeared to be driven entirely by one variant in the *APOC1* gene region, and may therefore represent horizontally pleiotropic effects of this variant rather than a causal effect of Alzheimer’s disease on brain structure.

Our finding of a protective causal effect of education on Alzheimer’s disease risk is consistent with the large body of observational epidemiological literature.^1–5,7^ In the current study, the IVW estimate suggested that every additional 4.2 YOS reduced the odds of Alzheimer’s disease by ∼30%. This effect size is broadly consistent with the findings of a dose-response meta-analysis, in which every additional year of schooling was associated with a 7% reduction in risk of all-cause dementia.^112^ Our results are also consistent with previous MR evidence supporting a causally protective effect of education on Alzheimer’s disease risk.^65–69^ Evidence from three recent multivariable MR studies suggests that the protective effect of EA on Alzheimer’s disease is largely mediated via its effects on later intelligence, rather than an independent protective effect of YOS.^65,68,113^ EA and intelligence are phenotypically^114^ and genetically^115,116^ correlated, and although there is a degree of non-overlap between the genetic predictors of the two traits,^65^ we note that the instruments used to proxy EA in our analyses are unlikely to be uniquely associated with years of education. Therefore, the results of the present study may not be informing us narrowly about time spent in formal education in childhood and adolescence, but rather a proxy measure that reflects EA (and to some extent, intelligence) more broadly. We have focused on EA as the exposure of interest as it is a potentially modifiable factor, and has been suggested to be ‘the most consistent, robust and durable method yet to be identified for raising intelligence levels’.^117^ Indeed, a large meta-analysis of quasi-experimental studies estimated every additional year of education to be associated with an increase of ∼1–5 standardized intelligence quotient points,^117^ a result that was consistent across the lifespan and across broad categories of cognitive ability. If, as previous multivariable MR evidence suggests, intelligence lies on the causal pathway from education to Alzheimer’s disease, increasing the number of YOS could still be an effective public health strategy for reducing the incidence of Alzheimer’s disease, even if the protective effect of higher EA is entirely mediated via increased intelligence levels. Findings from a quasi-experimental study suggest that increasing the school-leaving age in the UK in 1972 has led to improvements in intelligence quotient as well as a range of health and well-being outcomes, including reduced risks of diabetes and all-cause mortality.^37^ Our results indicate that education policy reforms that target the length of compulsory schooling are also likely to be effective for the primary prevention of Alzheimer’s disease at the population level.^65,69^

We found evidence for bidirectional causal relationships between EA and three global macro-structural markers of cortical morphology, namely, volume, surface area and intrinsic curvature. These findings are in agreement with the existing observational neuroimaging literature on the association between EA and brain morphology,^24,26,32^ as well as evidence for positive genetic correlations between education and MRI markers of cortical macro-structure.^71,118^ To investigate the direction of causation in these associations, we performed sensitivity analyses using Steiger filtering where we excluded any SNPs that explained more variance in the outcome than in the exposure. Although the magnitude of the effect size attenuated in all analyses following the removal of these SNPs, all causal effect estimates remained statistically significant. We note that when a bidirectional MR analysis suggests causal effects in both directions, it is difficult to settle the question of whether these truly represent reciprocal causality or may reflect shared aetiology, whereby the genetic variants used as instrumental variables in both analyses predict a common cause of both traits. Thus, while all our sensitivity analyses suggest distinct causal effects in both directions between EA and these macro-structural markers, the shared aetiology explanation is also possible. We also found evidence for causal effects of EA on local gyrification index and on the total volume of white matter hyperintensities. However, when we removed variants that explained more variance in the outcome than in the exposure, these effects were no longer statistically significant, suggesting that they may be explained by reverse causation or horizontal pleiotropy. As white matter hyperintensities mainly develop in elderly individuals,^87^ reverse causation is unlikely in this case and the association with EA probably reflects horizontal pleiotropy.

The observed causal effect of EA on these macro-structural metrics may arise from the beneficial effects of education on neurodevelopment in childhood/adolescence, and/or from its protective effects on neurodegeneration in adulthood/ageing. As we have used GWAS of brain structure measured cross-sectionally in middle to old age, we cannot prioritize either of the two hypotheses in the current study. However, there are several observational studies that have attempted to distinguish between the effects of education on neurodevelopment and neurodegeneration. For instance, a recent study addressed this question by examining the association of EA with total grey matter volume and ICV in both developmental and adult cohorts.^119^ Grey matter volume shows marked changes across the lifespan, increasing substantially along with cortical surface expansion in early childhood^120,121^ and decreasing through adulthood with reductions in cortical thickness and subcortical volumes.^122–124^ ICV, on the other hand, increases until mid-to-late adolescence, with little if any changes observed after,^125^ and may therefore serve as a proxy for maximal neuroanatomical volume.^119^ If education was associated with enhanced brain maturation in childhood/adolescence, we would expect years of education to be strongly related to ICV. If, however, education had a neuroprotective effect in ageing, we would expect a specific association between higher EA and grey matter volume in adulthood/ageing, after controlling for ICV. The study found consistent positive associations between education and ICV in both development and adulthood, but there was no evidence that education was related to ICV-adjusted grey matter volumes in ageing cohorts,^119^ indicating a primarily developmental effect of education that is established in early life rather than a protective effect against neurodegeneration in ageing.^119^ This finding is also compatible with recent observational evidence on the association between education and rates of cognitive/brain ageing over the course of adulthood and old age. For instance, a longitudinal neuroimaging study of >2000 individuals found no evidence of an association between levels of education and longitudinal rates of volume loss in atrophy-prone cortical regions or in the hippocampus.^29^ Similarly, a recent review that examined the effects of EA on cognitive ageing^126^ concluded that while there was strong evidence of a positive association between education and level of cognitive functioning, associations between education and the rate of ageing-associated cognitive decline were negligible. Overall, these findings are more consistent with the account that the effects of education on brain structure and cognitive function are established primarily during neurodevelopment in childhood/adolescence, and are largely preserved into old age rather than being grounded in neuroprotective effects on cognitive/brain ageing.^29,119,126^ In other words, individuals with higher EA might have an initial advantage in terms of cognitive/brain reserve that persists through their adulthood/ageing. It is this advantage, rather than attenuated longitudinal changes in brain structure and cognition, which reduces the risk of being diagnosed with Alzheimer’s disease in more highly educated individuals (i.e. an intercept effect of education across the lifespan rather than an effect on the slope/rate of change).^29,119,126^

Across the 15 cross-sectional imaging-derived phenotypes examined, we did not find any evidence of a causal effect of brain structure on Alzheimer’s disease risk or for a mediating role of these brain structure phenotypes on the causal pathway from EA to Alzheimer’s disease. However, we cannot, on the basis of these findings, rule out the possibility that changes in brain structure mediate the protective effect of education on Alzheimer’s risk through determining the underlying brain reserve of the individual. It is possible that capturing the neural basis of brain reserve requires more granular and specific MRI metrics than the ones used in the current study. We reasoned that any putative protective effects of education on Alzheimer’s risk would be unlikely to be manifest only in a highly regionally specific manner, and hence decided to primarily study global rather than localized imaging phenotypes. Nevertheless, the use of global imaging metrics in the current study may have masked any significant regionally specific causal effects of brain macro- or micro-structure on Alzheimer’s disease risk.^32^ We only present analyses in the main paper based on cross-sectional brain imaging data, and it is possible that analyses based on longitudinal changes may differ—see Supplementary material p. 13 and Supplementary Table 7 for a preliminary analysis using data on brain structural longitudinal change. It is also plausible that the protective effect of education on Alzheimer’s disease is better explained by the related concept of ‘cognitive reserve’, which refers to the capacity of the brain to cope with pathology through more efficient use of pre-existing cognitive networks or via recruitment of compensatory neural pathways.^9,22,127^ Cognitive reserve is a dynamic and active process of adaptation, and is typically measured using behavioural testing or functional neuroimaging modalities (e.g. resting state or task-related networks of brain activation that moderate the effect of Alzheimer’s pathology on cognition).^9,128^ Although the concepts of cognitive and brain reserve are not mutually exclusive and are hypothesized to operate synergistically in moderating the effect of Alzheimer’s pathology on clinical outcome,^9,22^ it is possible that the protective effect of education on Alzheimer’s disease risk is largely mediated via increased cognitive reserve.^128^ Therefore, the use of functional, rather than structural MRI, or cognitive/behavioural phenotypes might be better suited to capturing such mediating effects. Additionally, some of the putative benefits of education on Alzheimer’s risk could be mediated primarily through mechanisms minimally related to brain MRI metrics, such as reduced smoking and improved vascular health^5^ or via other brain processes (e.g. microscopic or intracellular or vascular) that cannot be captured well by our MRI metrics.

In the reverse direction, we found evidence for an association of genetically predicted Alzheimer’s disease with cortical orientation dispersion index, mean diffusivity in the white matter tracts and bilateral hippocampal volumes. However, these results were entirely driven by a single intron variant in the *APOC1* gene region (rs12721046), which has been associated with cerebral amyloid deposition as measured by PET imaging in a recent GWAS.^129^ The *APOC1* gene encodes a member of the apolipoprotein C1 family, which plays a central role in the regulation of lipid levels and metabolism.^130^ It is located in a cluster on chromosome 19, ∼5 kilobase pairs downstream from the *APOE* gene,^130^ where common genetic variation is known to have a relatively large effect on Alzheimer’s disease risk.^131,132^ A recent fine-mapping study of the *APOE* and surrounding regions using whole-genome sequencing data identified a cluster of risk variants (including rs12721046) in the *APOC1* region as potential causal variants for Alzheimer’s disease.^133^ This cluster of variants were found to confer increased risk of Alzheimer’s independent of the *APOE*-ε4 genotype.^133^ In all four analyses, the precision of the variant-specific causal estimate for rs12721046 was substantially higher than the remaining instruments, causing this variant to dominate the IVW estimate. When this variant was removed from the analysis, the overall causal effect estimate was attenuated towards the null and was no longer significant. Since all causal effects were only evidenced by the rs12721046 variant, they may represent horizontally pleiotropic pathways from this variant to brain structure. Therefore, we cannot draw definitive conclusions regarding the effect of genetic liability to Alzheimer’s disease on brain structure.

Our findings should be interpreted in the context of some limitations. First, although we have used imaging-derived phenotypes as markers of structural brain reserve, we cannot determine the extent to which these phenotypes capture pre-morbid brain reserve, as opposed to neuropathological changes secondary to disease (e.g. atrophy secondary to Alzheimer’s disease).^22^ Given the age range of the UK Biobank imaging cohort (45–82 years),^73^ the possibility that some individuals in the imaging GWAS harbour pre-clinical Alzheimer’s disease cannot be excluded. Using both self-reported diagnoses and International Classification of Diseases^134^ codes, we found that only 0.6% of participants in the UK Biobank have a current Alzheimer’s diagnosis. Therefore, it is unlikely that a large proportion of the imaging GWAS sample had current or imminent Alzheimer’s disease at the time of scanning. Nevertheless, with increasing duration of follow-up, a substantial proportion of these participants may go on to develop Alzheimer’s or other dementias.

Second, causal effect estimates in our analyses might be affected by Winner’s curse bias, which occurs when the same dataset is used to select the genetic variants as instrumental variables and to estimate their association with the exposure.^135^ Although this bias could be avoided by using three non-overlapping datasets from the same underlying population for genetic discovery and the estimation of variant-exposure and variant-outcome associations, restricting our analyses to non-overlapping datasets for the phenotypes examined would have substantially reduced the sample sizes. Therefore, a compromise was necessary to balance the risk of bias against imprecision of the causal estimates.^64^

Third, although there were no overlapping samples in the analyses of education and brain structure on Alzheimer’s disease, 39% of participants in the EA GWAS and all of those in the brain imaging GWAS were recruited from the UK Biobank. Overlap between the exposure and outcome datasets in two-sample MR can exacerbate bias due to weak instruments,^91^ with the magnitude of bias being a linear function of the degree of overlap between the two samples.^136^ However, brain imaging is a recent addition to the UK Biobank data collection protocol, and <10% of the UK Biobank cohort contribute to the imaging sample used in the present study. Even if all participants from the UK Biobank imaging sample were included in the EA GWAS, the percentage overlap between the exposure and outcome datasets in the current study would range from 2.8 to 2.9% for the education → brain structure analyses, and from 4.1 to 4.3% for the brain structure → education analyses, suggesting that the extent of sample overlap in the current study is minimal. Therefore, it is unlikely that the use of completely non-overlapping samples would have changed the direction or magnitude of our results.

Fourth, our findings may have been affected by selection bias in the UK Biobank imaging cohort, which occurs when selection into the study sample depends on a collider.^137^ In MR, both the exposure and the outcome are causally downstream of the genetic variants and any confounders of the exposure–outcome association (i.e. they are colliders of the instrumental variable and the confounder). Therefore, if selection into the study sample is a function of the exposure or the outcome, this can introduce an association between the instrumental variable and the confounder^138^ leading to a violation of the instrumental variable assumptions.^139^ In the analyses assessing the causal effect of EA on imaging-derived phenotypes, selection into the imaging sample is likely to have been a function of the exposure, as participants in the UK Biobank have higher education levels compared to the UK general population.^140,141^ Selection bias could have also occurred in the analyses assessing the causal effect of brain structure on Alzheimer’s disease, as recruitment into the imaging sample may have been a function of the outcome. Since UK Biobank participants are healthier than the general population,^142^ and the imaging cohort is a subset of those participants who have volunteered to undergo extensive additional assessments,^141,143^ it is reasonable to assume that those recruited into the imaging cohort were less likely to have Alzheimer’s disease at the time of scanning compared to the general population (or develop Alzheimer’s disease in the future). However, simulation studies have shown that the impact of selection bias on MR estimates and Type I error rates is only severe when the collider has a particularly large effect on selection,^139^ which is unlikely to be the case in the examples discussed previously. Therefore, we believe it is unlikely that our findings can be explained solely by selection bias.

Fifth, genetic association estimates with several phenotypes were taken from GWAS based on the UK Biobank cohort, which is not representative of the general population with regards to several sociodemographic, lifestyle and health-related characteristics. For instance, UK Biobank participants are more likely to be female, older and living in less socioeconomically deprived areas compared with non-participants.^142^ They have also been shown to have a lower prevalence of obesity, smoking and daily alcohol consumption compared with the UK general population, as well as lower age-adjusted rates of total cancer incidence and all-cause mortality.^142^ These differences are consistent with the well-established ‘healthy volunteer effect’,^144^ which may limit the generalizability of our findings.

Sixth, to minimize the possibility of confounding due to population stratification, we limited our analyses to participants of European ancestry. Therefore, it remains unclear whether our findings will extrapolate to other populations.

Finally, the GWAS data sources used for brain imaging phenotypes had relatively small sample sizes. Although larger GWAS of brain structure are available,^71,145^ these are meta-analyses of MRI data from different imaging cohorts. The use of uniform genotyping and neuroimaging protocols in the UK Biobank and the application of consistent genetic and phenotypic quality control pipelines to the data used in the current study is likely to have resulted in lower measurement error and enhanced power, despite the slightly smaller sample size. Nevertheless, statistical power calculations indicated that our analyses may have been underpowered to detect particularly subtle causal effects of brain structure on Alzheimer’s risk (Supplementary Table 1). The imaging sample on which the present study is based represents only 40% of the eventual UK Biobank imaging cohort size.^73^ As data from more participants become available, replication of these findings using larger and better-powered GWAS of imaging-derived phenotypes will enable us to draw more definitive conclusions regarding putative causal relationships between education, brain structure and Alzheimer’s disease.

In conclusion, our findings add to the extensive body of observational epidemiological literature, as well as evidence from a growing number of MR investigations, providing support for a causally protective role of increased EA on risk of Alzheimer’s disease. In addition, our results support potential bidirectional causal associations between education and several aspects of cortical macro- and micro-structure. However, we found no evidence that these structural alterations have downstream effects on risk of Alzheimer’s or that they lie on the causal pathway from education to Alzheimer’s disease. The observed protective effect of increased EA on risk of Alzheimer’s may be mediated via other structural brain changes not captured by these particular MRI phenotypes or by alternative biological mechanisms.

## Supplementary Material

awac392_Supplementary_Data

## References

[1] Caamaño-Isorna F , Corral M, Montes-Martínez A, Takkouche B. Education and dementia: A meta-analytic study. Neuroepidemiology. 2006;26:226–232. 16707907 10.1159/000093378

[2] Sharp ES , Gatz M. Relationship between education and dementia. Alz Dis Assoc Dis. 2011;25:289–304. 10.1097/WAD.0b013e318211c83c PMC3193875 21750453

[3] Meng X , D’Arcy C. Education and dementia in the context of the cognitive reserve hypothesis: A systematic review with meta-analyses and qualitative analyses. PLoS ONE. 2012;7:e38268. 22675535 10.1371/journal.pone.0038268 PMC3366926

[4] Livingston G , Sommerlad A, Orgeta V, et al Dementia prevention, intervention, and care. Lancet. 2017;390:2673–2734. 28735855 10.1016/S0140-6736(17)31363-6

[5] Livingston G , Huntley J, Sommerlad A, et al Dementia prevention, intervention, and care: 2020 report of the Lancet Commission. Lancet. 2020;396:413–446. 32738937 10.1016/S0140-6736(20)30367-6 PMC7392084

[6] Yu JT , Xu W, Tan CC, et al Evidence-based prevention of Alzheimer’s disease: Systematic review and meta-analysis of 243 observational prospective studies and 153 randomised controlled trials. J Neurology Neurosurg Psychiatry. 2020;91:1201–1209. 10.1136/jnnp-2019-321913 PMC7569385 32690803

[7] Satizabal CL , Beiser AS, Chouraki V, Chêne G, Dufouil C, Seshadri S. Incidence of dementia over three decades in the Framingham Heart Study. New Engl J Med. 2016;374:523–532. 26863354 10.1056/NEJMoa1504327 PMC4943081

[8] Norton S , Matthews FE, Barnes DE, Yaffe K, Brayne C. Potential for primary prevention of Alzheimer’s disease: An analysis of population-based data. Lancet Neurol. 2014;13:788–794. 25030513 10.1016/S1474-4422(14)70136-X

[9] Stern Y . Cognitive reserve in ageing and Alzheimer’s disease. Lancet Neurol. 2012;11:1006–1012. 23079557 10.1016/S1474-4422(12)70191-6 PMC3507991

[10] Perneczky R , Kempermann G, Korczyn AD, et al Translational research on reserve against neurodegenerative disease: Consensus report of the international conference on cognitive reserve in the dementias and the Alzheimer’s association reserve, resilience and protective factors professional interest area working groups. BMC Med. 2019;17:47. 30808345 10.1186/s12916-019-1283-z PMC6391801

[11] Satz P . Brain reserve capacity on symptom onset after brain injury: A formulation and review of evidence for threshold theory. Neuropsychology. 1993;7:273–295.

[12] Mak E , Su L, Williams GB, et al Progressive cortical thinning and subcortical atrophy in dementia with Lewy bodies and Alzheimer’s disease. Neurobiol Aging. 2015;36:1743–1750. 25649023 10.1016/j.neurobiolaging.2014.12.038

[13] Singh V , Chertkow H, Lerch JP, Evans AC, Dorr AE, Kabani NJ. Spatial patterns of cortical thinning in mild cognitive impairment and Alzheimer’s disease. Brain. 2006;129:2885–2893. 17008332 10.1093/brain/awl256

[14] Koval I , Bône A, Louis M, et al AD Course map charts Alzheimer’s disease progression. Sci Rep. 2021;11:8020. 33850174 10.1038/s41598-021-87434-1 PMC8044144

[15] Krumm S , Kivisaari SL, Probst A, et al Cortical thinning of parahippocampal subregions in very early Alzheimer’s disease. Neurobiol Aging. 2016;38:188–196. 26827657 10.1016/j.neurobiolaging.2015.11.001

[16] Schroeter ML , Stein T, Maslowski N, Neumann J. Neural correlates of Alzheimer’s disease and mild cognitive impairment: A systematic and quantitative meta-analysis involving 1351 patients. Neuroimage. 2009;47:1196–1206. 19463961 10.1016/j.neuroimage.2009.05.037 PMC2730171

[17] Yang J , Pan P, Song W, et al Voxelwise meta-analysis of gray matter anomalies in Alzheimer’s disease and mild cognitive impairment using anatomic likelihood estimation. J Neurol Sci. 2012;316:21–29. 22385679 10.1016/j.jns.2012.02.010

[18] Stahl R , Dietrich O, Teipel SJ, Hampel H, Reiser MF, Schoenberg SO. White matter damage in Alzheimer disease and mild cognitive impairment: Assessment with diffusion-tensor MR imaging and parallel imaging techniques. Radiology. 2007;243:483–492. 17456872 10.1148/radiol.2432051714

[19] Bozzali M , Falini A, Franceschi M, et al White matter damage in Alzheimer’s disease assessed in vivo using diffusion tensor magnetic resonance imaging. J Neurol Neurosurg Psychiatry. 2002;72:742–746. 12023417 10.1136/jnnp.72.6.742 PMC1737921

[20] Naggara O , Oppenheim C, Rieu D, et al Diffusion tensor imaging in early Alzheimer’s disease. Psychiatry Res. 2006;146:243–249. 16520023 10.1016/j.pscychresns.2006.01.005

[21] Takahashi S , Yonezawa H, Takahashi J, Kudo M, Inoue T, Tohgi H. Selective reduction of diffusion anisotropy in white matter of Alzheimer disease brains measured by 3.0 Tesla magnetic resonance imaging. Neurosci Lett. 2002;332:45–48. 12377381 10.1016/s0304-3940(02)00914-x

[22] Stern Y , Arenaza-Urquijo EM, Bartrés-Faz D, et al Whitepaper: Defining and investigating cognitive reserve, brain reserve, and brain maintenance. Alzheimers Dement. 2020;16:1305–1311. 30222945 10.1016/j.jalz.2018.07.219 PMC6417987

[23] Van Loenhoud AC , Groot C, Vogel JW, van der Flier WM, Ossenkoppele R. Is intracranial volume a suitable proxy for brain reserve? Alzheimers Res Ther. 2018;10:91. 30205838 10.1186/s13195-018-0408-5 PMC6134772

[24] Arenaza-Urquijo EM , Landeau B, Joie RL, et al Relationships between years of education and gray matter volume, metabolism and functional connectivity in healthy elders. Neuroimage. 2013;83:450–457. 23796547 10.1016/j.neuroimage.2013.06.053

[25] Bartrés-Faz D , Solé-Padullés C, Junqué C, et al Interactions of cognitive reserve with regional brain anatomy and brain function during a working memory task in healthy elders. Biol Psychol. 2009;80:256–259. 19022337 10.1016/j.biopsycho.2008.10.005

[26] Solé-Padullés C , Bartrés-Faz D, Junqué C, et al Brain structure and function related to cognitive reserve variables in normal aging, mild cognitive impairment and Alzheimer’s disease. Neurobiol Aging. 2009;30:1114–1124. 18053618 10.1016/j.neurobiolaging.2007.10.008

[27] Vaqué-Alcázar L , Sala-Llonch R, Valls-Pedret C, et al Differential age-related gray and white matter impact mediates educational influence on elders’ cognition. Brain Imaging Behav. 2017;11:318–332. 27535872 10.1007/s11682-016-9584-8

[28] Bartrés-Faz D , Arenaza-Urquijo EM. Structural and functional imaging correlates of cognitive and brain reserve hypotheses in healthy and pathological aging. Brain Topogr. 2011;24:340–357. 21853422 10.1007/s10548-011-0195-9

[29] Nyberg L , Magnussen F, Lundquist A, et al Educational attainment does not influence brain aging. Proc National Acad Sci U S A. 2021;118:e2101644118. 10.1073/pnas.2101644118 PMC8106299 33903255

[30] Kim JP , Seo SW, Shin HY, et al Effects of education on aging-related cortical thinning among cognitively normal individuals. Neurology. 2015;85:806–812. 26231257 10.1212/WNL.0000000000001884

[31] Liu Y , Julkunen V, Paajanen T, et al Education increases reserve against Alzheimer’s disease—Evidence from structural MRI analysis. Neuroradiology. 2012;54:929–938. 22246242 10.1007/s00234-012-1005-0 PMC3435513

[32] Tang X , Varma VR, Miller MI, Carlson MC. Education is associated with sub-regions of the hippocampus and the amygdala vulnerable to neuropathologies of Alzheimer’s disease. Brain Struct Funct. 2017;222:1469–1479. 27535407 10.1007/s00429-016-1287-9 PMC5850930

[33] Teipel SJ , Meindl T, Wagner M, et al White matter microstructure in relation to education in aging and Alzheimer’s disease. J Alzheimers Dis. 2009;17:571–583. 19433891 10.3233/JAD-2009-1077

[34] Smith GD , Hemani G. Mendelian Randomization: Genetic anchors for causal inference in epidemiological studies. Hum Mol Genet. 2014;23:R89–R98. 25064373 10.1093/hmg/ddu328 PMC4170722

[35] Jednoróg K , Altarelli I, Monzalvo K, et al The influence of socioeconomic status on children’s brain structure. PLoS ONE. 2012;7:e42486. 22880000 10.1371/journal.pone.0042486 PMC3411785

[36] Noble KG , Houston SM, Brito NH, et al Family income, parental education and brain structure in children and adolescents. Nat Neurosci. 2015;18:773–778. 25821911 10.1038/nn.3983 PMC4414816

[37] Davies NM , Dickson M, Smith GD, van den Berg GJ, Windmeijer F. The causal effects of education on health outcomes in the UK Biobank. Nat Hum Behav. 2018;2:117–125. 30406209 10.1038/s41562-017-0279-y PMC6217998

[38] Jefferis BJMH , Power C, Hertzman C. Birth weight, childhood socioeconomic environment, and cognitive development in the 1958 British birth cohort study. BMJ. 2002;325:305. 12169505 10.1136/bmj.325.7359.305 PMC117769

[39] Allin M , Henderson M, Suckling J, et al Effects of very low birthweight on brain structure in adulthood. Dev Med Child Neurol. 2003;46:46–53. 10.1017/s0012162204000088 14974647

[40] Taylor HG , Filipek PA, Juranek J, Bangert B, Minich N, Hack M. Brain volumes in adolescents with very low birth weight: Effects on brain structure and associations with neuropsychological outcomes. Dev Neuropsychol. 2011;36:96–117. 21253993 10.1080/87565641.2011.540544

[41] Farajdokht F , Sadigh-Eteghad S, Dehghani R, et al Very low birth weight is associated with brain structure abnormalities and cognitive function impairments: A systematic review. Brain Cogn. 2017;118:80–89. 28802183 10.1016/j.bandc.2017.07.006

[42] Cox SR , Dickie DA, Ritchie SJ, et al Associations between education and brain structure at age 73 years, adjusted for age 11 IQ. Neurology. 2016;87:1820–1826. 27664981 10.1212/WNL.0000000000003247 PMC5089529

[43] Bublitz MH , Stroud LR. Maternal smoking during pregnancy and offspring brain structure and function: Review and agenda for future research. Nicotine Tob Res. 2011;14:388–397. 22180574 10.1093/ntr/ntr191 PMC3313781

[44] Allan CL , Zsoldos E, Filippini N, et al Lifetime hypertension as a predictor of brain structure in older adults: Cohort study with a 28-year follow-up. Br J Psychiatry. 2015;206:308–315. 25497301 10.1192/bjp.bp.114.153536 PMC4381190

[45] Lane CA , Barnes J, Nicholas JM, et al Associations between blood pressure across adulthood and late-life brain structure and pathology in the neuroscience substudy of the 1946 British birth cohort (insight 46): An epidemiological study. Lancet Neurol. 2019;18:942–952. 31444142 10.1016/S1474-4422(19)30228-5 PMC6744368

[46] Shang X , Hill E, Zhu Z, et al The Association of Age at Diagnosis of Hypertension with Brain Structure and Incident Dementia in the UK Biobank. Hypertension. 2021;78:1463–1474. 34601962 10.1161/HYPERTENSIONAHA.121.17608

[47] Maillard P , Seshadri S, Beiser A, et al Effects of systolic blood pressure on white-matter integrity in young adults in the Framingham heart study: A cross-sectional study. Lancet Neurol. 2012;11:1039–1047. 23122892 10.1016/S1474-4422(12)70241-7 PMC3510663

[48] Gray JC , Thompson M, Bachman C, Owens MM, Murphy M, Palmer R. Associations of cigarette smoking with gray and white matter in the UK Biobank. Neuropsychopharmacology. 2020;45:1215–1222. 32032968 10.1038/s41386-020-0630-2 PMC7235023

[49] Cox SR , Lyall DM, Ritchie SJ, et al Associations between vascular risk factors and brain MRI indices in UK Biobank. Eur Heart J. 2019;40:2290–2300. 30854560 10.1093/eurheartj/ehz100 PMC6642726

[50] Guan Y , Ebrahimzadeh SA, Cheng CH, et al Association of diabetes and hypertension with brain structural integrity and cognition in the Boston Puerto Rican Health study cohort. Neurology. 2022;98:e1534–e1544. 35354581 10.1212/WNL.0000000000200120 PMC9012269

[51] Mackey S , Allgaier N, Chaarani B, et al Mega-analysis of gray matter volume in substance dependence: General and substance-specific regional effects. Am J Psychiatry. 2019;176:119–128. 30336705 10.1176/appi.ajp.2018.17040415 PMC6427822

[52] Daviet R , Aydogan G, Jagannathan K, et al Associations between alcohol consumption and gray and white matter volumes in the UK Biobank. Nat Commun. 2022;13:1175. 35246521 10.1038/s41467-022-28735-5 PMC8897479

[53] Hamer M , Sharma N, Batty GD. Association of objectively measured physical activity with brain structure: UK Biobank study. J Intern Med. 2018;284:439–443. 29776014 10.1111/joim.12772

[54] Benedict C , Brooks SJ, Kullberg J, et al Association between physical activity and brain health in older adults. Neurobiol Aging. 2013;34:83–90. 22592017 10.1016/j.neurobiolaging.2012.04.013

[55] Domingos C , Pêgo JM, Santos NC. Effects of physical activity on brain function and structure in older adults: A systematic review. Behav Brain Res. 2020;402:113061. 33359570 10.1016/j.bbr.2020.113061

[56] Smith GD , Ebrahim S. ‘Mendelian randomization’: Can genetic epidemiology contribute to understanding environmental determinants of disease? Int J Epidemiol. 2003;32:1–22. 12689998 10.1093/ije/dyg070

[57] Evans DM , Smith GD. Mendelian randomization: New applications in the coming age of hypothesis-free causality. Annu Rev Genom Hum G. 2015;16:327–350. 10.1146/annurev-genom-090314-050016 25939054

[58] Smith GD , Ebrahim S. What can Mendelian randomisation tell us about modifiable behavioural and environmental exposures? BMJ. 2005;330:1076–1079. 15879400 10.1136/bmj.330.7499.1076 PMC557238

[59] Wehby GL , Ohsfeldt RL, Murray JC. ‘Mendelian randomization’ equals instrumental variable analysis with genetic instruments. Stat Med. 2008;27:2745–2749. 18344186 10.1002/sim.3255 PMC2706420

[60] Thomas DC , Conti DV. Commentary: The concept of ‘Mendelian randomization’. Int J Epidemiol. 2004;33:21–25. 15075141 10.1093/ije/dyh048

[61] Ebrahim S , Smith GD. Mendelian randomization: Can genetic epidemiology help redress the failures of observational epidemiology? Hum Genet. 2008;123:15–33. 18038153 10.1007/s00439-007-0448-6

[62] Smith GD , Lawlor DA, Harbord R, Timpson N, Day I, Ebrahim S. Clustered environments and randomized genes: A fundamental distinction between conventional and genetic epidemiology. PLoS Med. 2007;4:e352. 18076282 10.1371/journal.pmed.0040352 PMC2121108

[63] Taylor M , Tansey K, Lawlor D, et al Testing the principles of Mendelian randomization: Opportunities and complications on a genomewide scale, biorXiv, 10.1101/124362, 7 April 2017, preprint: not peer reviewed.

[64] Burgess S , Thompson SG. Mendelian randomization: Methods for Causal Inference Using Genetic Variants; 2nd ed. CRC Press, Taylor & Francis Group; 2021.

[65] Anderson EL , Howe LD, Wade KH, et al Education, intelligence and Alzheimer’s disease: Evidence from a multivariable two-sample Mendelian randomization study. Int J Epidemiol. 2020;49:1163–1172. 32003800 10.1093/ije/dyz280 PMC7660137

[66] Larsson SC , Traylor M, Malik R, et al Modifiable pathways in Alzheimer’s disease: Mendelian randomisation analysis 2017;359:j5375. 10.1136/bmj.j5375 PMC5717765 29212772

[67] Raghavan NS , Vardarajan B, Mayeux R. Genomic variation in educational attainment modifies Alzheimer disease risk. Neurol Genet. 2019;5:e310. 30863791 10.1212/NXG.0000000000000310 PMC6395060

[68] Lord J , Green R, Choi SW, et al Disentangling independent and mediated causal relationships between blood metabolites, cognitive factors, and Alzheimer’s disease. Biol Psychiatry Glob Open Sci. 2021;2:167–179. 36325159 10.1016/j.bpsgos.2021.07.010 PMC9616368

[69] Anderson EL , Wade KH, Hemani G, et al The causal effect of educational attainment on Alzheimer’s disease: A two-sample Mendelian randomization study, biorxiv, 10.1101/127993, 17 April 2017, preprint: not peer reviewed.

[70] Knutson KA , Deng Y, Pan W. Implicating causal brain imaging endophenotypes in Alzheimer’s disease using multivariable IWAS and GWAS summary data. Neuroimage. 2020;223:117347. 32898681 10.1016/j.neuroimage.2020.117347 PMC7778364

[71] Grasby KL , Jahanshad N, Painter JN, et al The genetic architecture of the human cerebral cortex. Science. 2020;367:eaay6690. 10.1126/science.aay6690 PMC7295264 32193296

[72] Elliott LT , Sharp K, Alfaro-Almagro F, et al Genome-wide association studies of brain imaging phenotypes in UK Biobank. Nature. 2018;562:210–216. 30305740 10.1038/s41586-018-0571-7 PMC6786974

[73] Smith SM , Douaud G, Chen W, et al An expanded set of genome-wide association studies of brain imaging phenotypes in UK Biobank. Nat Neurosci. 2021;24:737–745. 33875891 10.1038/s41593-021-00826-4 PMC7610742

[74] Zhao B , Li T, Yang Y, et al Common genetic variation influencing human white matter microstructure. Science. 2021;372:eabf3736. 10.1126/science.abf3736 PMC8370718 34140357

[75] Burgess S , Thompson SG. Multivariable Mendelian randomization: The use of pleiotropic genetic variants to estimate causal effects. Am J Epidemiol. 2015;181:251–260. 25632051 10.1093/aje/kwu283 PMC4325677

[76] Carter AR , Sanderson E, Hammerton G, et al Mendelian Randomisation for mediation analysis: Current methods and challenges for implementation. Eur J Epidemiol. 2021;36:465–478. 33961203 10.1007/s10654-021-00757-1 PMC8159796

[77] Burgess S , Daniel RM, Butterworth AS, Thompson SG, EPIC-InterAct Consortium. Network Mendelian randomization: Using genetic variants as instrumental variables to investigate mediation in causal pathways. Int J Epidemiol. 2015;44:484–495. 25150977 10.1093/ije/dyu176 PMC4469795

[78] Inoue A , Solon G. Two-sample instrumental variables estimators. Rev Econ Stat. 2010;92:557–561.

[79] Lawlor DA . Commentary: Two-sample Mendelian randomization: Opportunities and challenges. Int J Epidemiol. 2016;45:908–915. 27427429 10.1093/ije/dyw127 PMC5005949

[80] Lee JJ , Wedow R, Okbay A, et al Gene discovery and polygenic prediction from a genome-wide association study of educational attainment in 1.1 million individuals. Nat Genet. 2018;50:1112–1121. 30038396 10.1038/s41588-018-0147-3 PMC6393768

[81] Kunkle BW , Grenier-Boley B, Sims R, et al Genetic meta-analysis of diagnosed Alzheimer’s disease identifies new risk loci and implicates aβ, tau, immunity and lipid processing. Nat Genet. 2019;51:414–430. 30820047 10.1038/s41588-019-0358-2 PMC6463297

[82] Warrier V , Stauffer EM, Huang QQ, et al The genetics of cortical organisation and development: a study of 2,347 neuroimaging phenotypes, biorXiv, 10.1101/2022.09.08.507084, 8 September 2022, preprint: not peer reviewed.

[83] Sudlow C , Gallacher J, Allen N, et al UK Biobank: An open access resource for identifying the causes of a wide range of complex diseases of middle and old age. PLoS Med. 2015;12:e1001779. 10.1371/journal.pmed.1001779 PMC4380465 25826379

[84] Salthouse TA . Neuroanatomical substrates of age-related cognitive decline. Psychol Bull. 2011;137:753–784. 21463028 10.1037/a0023262 PMC3132227

[85] Garde E , Mortensen EL, Krabbe K, Rostrup E, Larsson HB. Relation between age-related decline in intelligence and cerebral white-matter hyperintensities in healthy octogenarians: A longitudinal study. Lancet. 2000;356:628–634. 10968435 10.1016/S0140-6736(00)02604-0

[86] Maillard P , Carmichael O, Fletcher E, Reed B, Mungas D, DeCarli C. Coevolution of white matter hyperintensities and cognition in the elderly. Neurology. 2012;79:442–448. 22815562 10.1212/WNL.0b013e3182617136 PMC3405254

[87] Moroni F , Ammirati E, Rocca MA, Filippi M, Magnoni M, Camici PG. Cardiovascular disease and brain health: Focus on white matter hyperintensities. Int J Cardiol Hear Vasc. 2018;19:63–69. 10.1016/j.ijcha.2018.04.006 PMC6016077 29946567

[88] Myers TA , Chanock SJ, Machiela MJ. LDlinkr: An R package for rapidly calculating linkage disequilibrium statistics in diverse populations. Front Genet. 2020;11:157. 32180801 10.3389/fgene.2020.00157 PMC7059597

[89] Hemani G , Bowden J, Smith GD. Evaluating the potential role of pleiotropy in Mendelian randomization studies. Hum Mol Genet. 2018;27:R195–R208. 29771313 10.1093/hmg/ddy163 PMC6061876

[90] Burgess S . Sample size and power calculations in Mendelian randomization with a single instrumental variable and a binary outcome. Int J Epidemiol. 2014;43:922–929. 24608958 10.1093/ije/dyu005 PMC4052137

[91] Burgess S , Smith GD, Davies NM, et al Guidelines for performing Mendelian randomization investigations. Wellcome Open Res. 2019;4:186. 32760811 10.12688/wellcomeopenres.15555.1 PMC7384151

[92] Burgess S , Butterworth A, Thompson SG. Mendelian Randomization analysis with multiple genetic variants using summarized data. Genet Epidemiol. 2013;37:658–665. 24114802 10.1002/gepi.21758 PMC4377079

[93] Burgess S , Dudbridge F, Thompson SG. Combining information on multiple instrumental variables in Mendelian randomization: Comparison of allele score and summarized data methods. Stat Med. 2016;35:1880–1906. 26661904 10.1002/sim.6835 PMC4832315

[94] Bowden J , Del Greco MF, Minelli C, Smith GD, Sheehan N, Thompson J. A framework for the investigation of pleiotropy in two-sample summary data Mendelian randomization. Stat Med. 2017;36:1783–1802. 28114746 10.1002/sim.7221 PMC5434863

[95] Parker RA , Weir CJ. Non-adjustment for multiple testing in multi-arm trials of distinct treatments: Rationale and justification. Clin Trials. 2020;17:562–566. 32666813 10.1177/1740774520941419 PMC7534018

[96] Burgess S , Foley CN, Allara E, Staley JR, Howson JMM. A robust and efficient method for Mendelian randomization with hundreds of genetic variants. Nat Commun. 2020;11:376. 31953392 10.1038/s41467-019-14156-4 PMC6969055

[97] Bowden J , Smith GD, Haycock PC, Burgess S. Consistent estimation in Mendelian randomization with some invalid instruments using a weighted median estimator. Genet Epidemiol. 2016;40:304–314. 27061298 10.1002/gepi.21965 PMC4849733

[98] Bowden J , Smith GD, Burgess S. Mendelian Randomization with invalid instruments: Effect estimation and bias detection through Egger regression. Int J Epidemiol. 2015;44:512–525. 26050253 10.1093/ije/dyv080 PMC4469799

[99] Verbanck M , Chen CY, Neale B, Do R. Detection of widespread horizontal pleiotropy in causal relationships inferred from Mendelian randomization between complex traits and diseases. Nat Genet. 2018;50:693–698. 29686387 10.1038/s41588-018-0099-7 PMC6083837

[100] Slob EAW , Burgess S. A comparison of robust Mendelian randomization methods using summary data. Genet Epidemiol. 2020;44:313–329. 32249995 10.1002/gepi.22295 PMC7317850

[101] Greco MFD , Minelli C, Sheehan NA, Thompson JR. Detecting pleiotropy in Mendelian randomisation studies with summary data and a continuous outcome. Stat Med. 2015;34:2926–2940. 25950993 10.1002/sim.6522

[102] Bowden J , Hemani G, Smith GD. Invited commentary: Detecting individual and global horizontal pleiotropy in Mendelian randomization—A job for the humble heterogeneity statistic? Am J Epidemiol. 2018;187:2681–2685. 30188969 10.1093/aje/kwy185 PMC6269239

[103] Burgess S , Bowden J, Fall T, Ingelsson E, Thompson SG. Sensitivity analyses for robust causal inference from Mendelian randomization analyses with multiple genetic variants. Epidemiology. 2017;28:30–42. 27749700 10.1097/EDE.0000000000000559 PMC5133381

[104] Corbin LJ , Richmond RC, Wade KH, et al BMI As a modifiable risk factor for type 2 diabetes: Refining and understanding causal estimates using Mendelian randomization. Diabetes. 2016;65:3002–3007. 27402723 10.2337/db16-0418 PMC5279886

[105] Hemani G , Tilling K, Smith GD. Orienting the causal relationship between imprecisely measured traits using GWAS summary data. PLoS Genet. 2017;13:e1007081. 10.1371/journal.pgen.1007081 PMC5711033 29149188

[106] Brouwer RM , Klein M, Grasby KL, et al Genetic variants associated with longitudinal changes in brain structure across the lifespan. Nat Neurosci. 2022;25:421–432. 35383335 10.1038/s41593-022-01042-4 PMC10040206

[107] Sanderson E , Smith GD, Windmeijer F, Bowden J. An examination of multivariable Mendelian randomization in the single-sample and two-sample summary data settings. Int J Epidemiol. 2018;48:713–727. 10.1093/ije/dyy262 PMC6734942 30535378

[108] Burgess S , Thompson DJ, Rees JMB, Day FR, Perry JR, Ong KK. Dissecting causal pathways using Mendelian randomization with summarized genetic data: Application to age at menarche and risk of breast cancer. Genetics. 2017;207:481–487. 28835472 10.1534/genetics.117.300191 PMC5629317

[109] MacKinnon DP , Lockwood CM, Hoffman JM, West SG, Sheets V. A comparison of methods to test mediation and other intervening variable effects. Psychol Methods. 2002;7:83–104. 11928892 10.1037/1082-989x.7.1.83 PMC2819363

[110] R Core Team . R: A language and environment for statistical computing. Vienna: R Foundation for Statistical Computing; 2015.

[111] Yavorska OO , Burgess S. Mendelian randomization: An R package for performing Mendelian randomization analyses using summarized data. Int J Epidemiol. 2017;46:1734–1739. 28398548 10.1093/ije/dyx034 PMC5510723

[112] Xu W , Tan L, Wang HF, et al Education and risk of dementia: Dose-response meta-analysis of prospective cohort studies. Mol Neurobiol. 2016;53:3113–3123. 25983035 10.1007/s12035-015-9211-5

[113] Grant AJ , Burgess S. Pleiotropy robust methods for multivariable Mendelian randomization. Stat Med. 2021;40:5813–5830. 34342032 10.1002/sim.9156 PMC7612169

[114] Strenze T . Intelligence and socioeconomic success: A meta-analytic review of longitudinal research. Intelligence. 2007;35:401–426.

[115] Hill WD , Marioni RE, Maghzian O, et al A combined analysis of genetically correlated traits identifies 187 loci and a role for neurogenesis and myelination in intelligence. Mol Psychiatry. 2019;24:169–181. 29326435 10.1038/s41380-017-0001-5 PMC6344370

[116] Johnson W , Deary IJ, Silventoinen K, Tynelius P, Rasmussen F. Family background buys an education in Minnesota but not in Sweden. Psychol Sci. 2009;21:1266–1273. 10.1177/0956797610379233 PMC2939922 20679521

[117] Ritchie SJ , Tucker-Drob EM. How much does education improve intelligence? A meta-analysis. Psychol Sci. 2018;29:1358–1369. 29911926 10.1177/0956797618774253 PMC6088505

[118] Liu S , Smit DJA, Abdellaoui A, van Wingen GA, Verweij KJH. Brain structure and function show distinct relations with genetic predispositions to mental health and cognition. medrXiv.[Preprint] doi:10.1101/2021.03.07.21252728 35961582

[119] Walhovd KB , Fjell AM, Wang Y, et al Education and income show heterogeneous relationships to lifespan brain and cognitive differences across European and US cohorts. Cereb Cortex. 2021;32:839–854. 10.1093/cercor/bhab248 PMC8841563 34467389

[120] Gilmore JH , Shi F, Woolson SL, et al Longitudinal development of cortical and subcortical gray matter from birth to 2 years. Cereb Cortex. 2012;22:2478–2485. 22109543 10.1093/cercor/bhr327 PMC3464410

[121] Li G , Nie J, Wang L, et al Mapping region-specific longitudinal cortical surface expansion from birth to 2 years of age. Cereb Cortex. 2013;23:2724–2733. 22923087 10.1093/cercor/bhs265 PMC3792744

[122] Storsve AB , Fjell AM, Tamnes CK, et al Differential longitudinal changes in cortical thickness, surface area and volume across the adult life span: Regions of accelerating and decelerating change. J Neurosci. 2014;34:8488–8498. 24948804 10.1523/JNEUROSCI.0391-14.2014 PMC6608217

[123] Walhovd KB , Westlye LT, Amlien I, et al Consistent neuroanatomical age-related volume differences across multiple samples. Neurobiol Aging. 2011;32:916–932. 19570593 10.1016/j.neurobiolaging.2009.05.013 PMC4040218

[124] Walhovd KB , Fjell AM, Reinvang I, et al Effects of age on volumes of cortex, white matter and subcortical structures. Neurobiol Aging. 2005;26:1261–1270. 16005549 10.1016/j.neurobiolaging.2005.05.020

[125] Mills KL , Goddings AL, Herting MM, et al Structural brain development between childhood and adulthood: Convergence across four longitudinal samples. Neuroimage. 2016;141:273–281. 27453157 10.1016/j.neuroimage.2016.07.044 PMC5035135

[126] Lövdén M , Fratiglioni L, Glymour MM, Lindenberger U, Tucker-Drob EM. Education and cognitive functioning across the life span. Psychol Sci Public Interest. 2020;21:6–41. 32772803 10.1177/1529100620920576 PMC7425377

[127] Barulli D , Stern Y. Efficiency, capacity, compensation, maintenance, plasticity: Emerging concepts in cognitive reserve. Trends Cogn Sci. 2013;17:502–509. 24018144 10.1016/j.tics.2013.08.012 PMC3840716

[128] Menardi A , Pascual-Leone A, Fried PJ, Santarnecchi E. The role of cognitive reserve in Alzheimer’s disease and aging: A multi-modal imaging review. J Alzheimers Dis. 2018;66:1341–1362. 30507572 10.3233/JAD-180549 PMC8972845

[129] Yan Q , Nho K, Del-Aguila JL, et al Genome-wide association study of brain amyloid deposition as measured by Pittsburgh compound-B (PiB)-PET imaging. Mol Psychiatry. 2021;26:309–321. 30361487 10.1038/s41380-018-0246-7 PMC6219464

[130] Woods AG , Sokolowska I, Taurines R, et al Potential biomarkers in psychiatry: Focus on the cholesterol system. J Cell Mol Med. 2012;16:1184–1195. 22304330 10.1111/j.1582-4934.2012.01543.x PMC3823072

[131] Liu CC , Liu CC, Kanekiyo T, Xu H, Bu G. Apolipoprotein E and Alzheimer disease: Risk, mechanisms and therapy. Nat Rev Neurol. 2013;9:106–118. 23296339 10.1038/nrneurol.2012.263 PMC3726719

[132] Bangen KJ , Beiser A, Delano-Wood L, et al APOE Genotype modifies the relationship between midlife vascular risk factors and later cognitive decline. J Stroke Cerebrovasc Dis. 2013;22:1361–1369. 23601373 10.1016/j.jstrokecerebrovasdis.2013.03.013 PMC3849195

[133] Zhou X , Chen Y, Mok KY, et al Non-coding variability at the APOE locus contributes to the Alzheimer’s risk. Nat Commun. 2019;10:3310. 31346172 10.1038/s41467-019-10945-z PMC6658518

[134] World Health Organization . ICD-10: International statistical classification of diseases and related health problems: Tenth revision. 2nd edn. World Health Organization; 2004.

[135] Mounier N , Kutalik Z. Correction for sample overlap, winner’s curse and weak instrument bias in two-sample Mendelian randomization. biorXiv. [Preprint] doi:10.1101/2021.03.26.437168

[136] Burgess S , Davies NM, Thompson SG. Bias due to participant overlap in two-sample Mendelian randomization. Genet Epidemiol. 2016;40:597–608. 27625185 10.1002/gepi.21998 PMC5082560

[137] Hernán MA , Hernández-Díaz S, Robins JM. A structural approach to selection bias. Epidemiology. 2004;15:615–625. 15308962 10.1097/01.ede.0000135174.63482.43

[138] Swanson SA , Robins JM, Miller M, Hernán MA. Selecting on treatment: A pervasive form of bias in instrumental variable analyses. Am J Epidemiol. 2015;181:191–197. 25609096 10.1093/aje/kwu284 PMC4312427

[139] Gkatzionis A , Burgess S. Contextualizing selection bias in Mendelian randomization: How bad is it likely to be? Int J Epidemiol. 2019;48:691–701. 30325422 10.1093/ije/dyy202 PMC6659463

[140] Batty GD , Gale CR, Kivimäki M, Deary IJ, Bell S. Comparison of risk factor associations in UK Biobank against representative, general population based studies with conventional response rates: Prospective cohort study and individual participant meta-analysis. BMJ. 2020;368:m131. 32051121 10.1136/bmj.m131 PMC7190071

[141] Bradley V , Nichols TE. Addressing selection bias in the UK Biobank neurological imaging cohort. medrXiv. [Preprint] doi:10.1101/2022.01.13.22269266

[142] Fry A , Littlejohns TJ, Sudlow C, et al Comparison of sociodemographic and health-related characteristics of UK Biobank participants with those of the general population. Am J Epidemiol. 2017;186:1026–1034. 28641372 10.1093/aje/kwx246 PMC5860371

[143] Littlejohns TJ , Holliday J, Gibson LM, et al The UK Biobank imaging enhancement of 100,000 participants: Rationale, data collection, management and future directions. Nat Commun. 2020;11:2624. 32457287 10.1038/s41467-020-15948-9 PMC7250878

[144] Delgado-Rodríguez M , Llorca J. Bias. J Epidemiol Community Health. 2004;58:635–641. 15252064 10.1136/jech.2003.008466 PMC1732856

[145] Satizabal CL , Adams HHH, Hibar DP, et al Genetic architecture of subcortical brain structures in 38,851 individuals. Nat Genet. 2019;51:1624–1636. 31636452 10.1038/s41588-019-0511-y PMC7055269

