# Supplementary material for: Educational attainment, structural brain reserve and Alzheimer’s disease: a Mendelian randomization analysis
Source: Brain. 2022 Oct 31;146(5):2059–74. doi: 10.1093/brain/awac392 (PMC10151197; doi:10.1093/brain/awac392)
Supplement: awac392_Supplementary_Data [file awac392_supplementary_data.docx]

# **Supplementary materials**

## **Supplementary methods**

### **Description of brain imaging-derived phenotypes**

Local gyrification index (LGI) is a measure of gyrification, the characteristic folding of the human cerebral cortex that emerges during development.^1^ This folding arises from the tangential expansion of the cortex, a process by which an increasingly expanding surface area is enclosed in a relatively small volume.^2,3^ LGI is a surface-based method for quantifying cortical gyrification, which takes into account the three-dimensional nature of the cortical surface, and is computed by estimating gyrification at thousands of points over the entire cortical area.^4^

Mean curvature (MC) is a measure of the extrinsic curvature of the cortical surface (i.e., the folding of the cortex that arises as a result of cortical surface expansion during development). This is an extrinsic property of the cortical surface, in that it is a function of how the surface is embedded in space, rather than reflecting the shape of the surface itself.^2^

Intrinsic curvature (IC) is a mathematically fundamental property of the cortical surface that is intrinsic to the geometry of the surface (i.e., unlike extrinsic curvature, it is not a function of how the cortex is folded in space and cannot be removed without deforming or tearing the surface).^1,5^ IC arises from differential growth between cortical regions,^1^ and has been suggested to be a more sensitive marker of neurodevelopmental abnormalities compared to larger scale measures of gyrification.^2^

Fractional anisotropy (FA) and mean diffusivity (MD) are two commonly used metrics derived from diffusion tensor imaging (DTI),^6^ the standard clinical diffusion MRI technique which enables the assessment of grey and white matter integrity. FA is a measure of the overall integrity and directionality of water diffusion,^7^ which is highly sensitive to general connectivity changes.^8^ MD is a measure reflecting the rotationally invariant magnitude of water diffusion within brain tissue.^7^ Although FA and MD are the most widely used surrogate measures of microstructural tissue change, they are non-specific markers for individual tissue microstructure features.^9^

The intracellular volume fraction (ICVF) and orientation dispersion index (ODI) phenotypes are both derived from neurite orientation dispersion and density imaging (NODDI),^10^ a diffusion MRI technique which was developed specifically for in vivo estimation of the microstructural properties of dendrites and axons – collectively referred to as neurites – in both white and grey matter. NODDI can be used to compartmentalize brain tissue into three microstructural environments with distinct diffusion properties: intra-cellular, extra-cellular, and cerebrospinal fluid. The intra-cellular compartment models the space occupied by the neurites, and can be used to quantify neurite morphology both in terms of their density (ICVF) and their spatial orientation (ODI).^10,11^ ICVF and ODI have been suggested to provide more specific markers of brain tissue microstructure compared to standard DTI-derived parameters such as FA and MD.^10^

### **Rationale for selection of brain imaging-derived phenotypes**

Although several thousand imaging-derived phenotypes can be generated from UK Biobank MRI data, many are highly inter-correlated, and analysis of all imaging-derived phenotypes could lead to spurious results or challenges in controlling for multiple comparisons. Therefore, we decided to focus our analyses on 15 brain imaging-derived phenotypes (Fig. 2), which were selected a-priori as plausible markers of structural brain reserve based on the available observational evidence.

We investigated six macro-structural MRI metrics (volume, cortical thickness [CT], surface area [SA], local gyrification index [LGI], mean curvature [MC], and intrinsic curvature [IC]) at the global cortical level. There is extensive observational evidence indicating that AD is associated with changes in these macro-structural markers, including widespread grey matter atrophy in cortical and sub-cortical regions,^12,13^ progressive cortical thinning,^14–17^ reduced regional surface areas,^18^ as well as reductions in regional mean curvature^19^ and local gyrification index.^20^ Furthermore, in observational neuroimaging studies of cognitively healthy individuals, education has been shown to be positively associated with some of these macro-structural measures, which is consistent with the brain reserve hypothesis. For instance, in cognitively healthy older adults, higher education has been associated with increased grey matter volume, both at the whole-brain level,^21^ as well as within specific regions, particularly in medial frontal and parietal areas.^22,23^ Similar findings have been reported for the association between education levels and cortical thickness in cognitively normal elderly, with more highly educated individuals showing increased global^24^ and regional cortical thickness, particularly in frontal lobe areas^25–27^ and temporal lobe regions, including the isthmus of cingulate gyrus, the temporal pole, and the transverse temporal gyrus.^28^ Evidence also supports a regionally specific effect of education on cortical surface area, with one study reporting positive associations between increased educational attainment in community-dwelling older adults and surface areas of the CA1 and subiculum regions of the hippocampus, as well as the basolateral subregion of the amygdala.^29^

We also investigated four micro-structural MRI metrics (fractional anisotropy [FA], mean diffusivity [MD], intracellular volume fraction [ICVF], and orientation dispersion index [ODI]) at the global cortical level. Although these phenotypes have been studied less often relative to macroscopic markers of cortical morphology, we chose to include them in our analysis as they capture more fine-grained grey matter micro-structural alterations in AD,^30–33^ which cannot be observed using conventional structural MRI sequences. Furthermore, emerging evidence indicates that these microscopic grey matter changes may predate the macro-structural atrophy that is characteristic of AD.^32,34,35^

In the white matter, we investigated two micro-structural metrics (FA and MD), averaged across 21 predefined cerebral white matter tracts. White matter tissue damage has previously been implicated in the pathogenesis of AD, with diffusion-weighted MRI studies reporting higher MD and lower FA in patients with AD compared to controls, both at the whole-brain and at the regional level.^36–39^ In addition, there is observational evidence for a positive effect of educational attainment on the integrity of pathologically relevant white matter fiber tracts.^26^ In cognitively normal elderly individuals, higher education has been associated with increased white matter tract integrity in several brain regions that are characteristic sites of AD pathology, including the medial temporal lobes, fusiform gyrus, insula, superior temporal gyrus, and lingual gyrus.^40^

In addition to the above whole-brain measures, we included one macro-structural MRI metric (volume), measured in a subcortical region (hippocampus). We focused on bilateral hippocampal volumes since the pathological hallmarks of AD (i.e., extra-cellular amyloid-β plaques and intra-cellular neurofibrillary tangles) are typically first observed in the hippocampi and surrounding medial temporal lobes regions,^41,42^ and that hippocampal atrophy is a robust anatomical precursor of the clinical symptoms of AD.^43,44^

Finally, we chose to study the total volume of white matter hyperintensities as a metric closely linked to age-related cognitive decline and thus a putative marker of structural brain health.^45–48^

### **Description of summary statistics data sources**

**GWAS of educational attainment by Lee *et al.*^49^**

The analysis combined summary statistics from 71 separate cohorts, all of which were restricted to individuals of European ancestry. Within each cohort, the major educational qualifications obtained by each individual were mapped onto the International Standard Classification of Education (ISCED) categories. The educational attainment phenotype was then constructed by imputing the equivalent years of education for each ISCED category. The sample-size-weighted mean (standard deviation) of the educational attainment phenotype was 16.8 (4.2) years of schooling across all cohorts. All association analyses were performed at the cohort level and adjusted for age, sex, their interaction, and genetic principal components. The sample-size weighted meta-analysis generated association estimates for approximately 10 million autosomal SNPs that passed the quality-control thresholds in all cohorts, of which 1,271 approximately independent SNPs (*r^2^* < 0.1) were genome-wide significant (*P* < 5 × 10^-8^).

A standard quality-control pipeline was applied to all cohort-level results, and genotypes were imputed using either the 1,000 Genomes Project Phase 3 European reference panel or a larger reference panel released by the Haplotype Reference Consortium. Subject-level filters included the exclusion of individuals of non-European ancestry, those with poor genotyping rates, and genetic outliers. In addition, individuals in whom the educational attainment phenotype was measured prior to age 30 were removed from the analysis. Genotype filters included the removal of INDELS and variants not located on autosomes, SNPs with known strand issues in imputation programs, SNPs with poor imputation accuracy, and those with minor allele counts < 25. Additionally, SNPs were excluded if they had invalid or duplicated chromosomal coordinates, if they had missing values for some of the variables (e.g., missing *P-*values), or if the alleles did not match those in the reference panel.

**GWAS of Late-onset Alzheimer’s disease by Kunkle *et al.*^50^**

The Stage 1 discovery sample (21,982 cases and 41,944 cognitively normal controls) was composed of 46 case-control datasets from the following four consortia: Alzheimer Disease Genetics Consortium (ADGC), Cohorts for Heart and Aging Research in Genomic Epidemiology Consortium (CHARGE), The European Alzheimer’s Disease Initiative (EADI), and Genetic and Environmental Risk in AD/Defining Genetic, Polygenic and Environmental Risk for Alzheimer’s Disease Consortium (GERAD/PERADES).

All association analyses were based on an additive genotype model and were adjusted for age (defined as age at onset of AD for cases and age at last examination for controls), sex, and principal components. The Stage 1 discovery meta-analysis was followed by Stage 2 replication in 8,362 cases and 10,483 controls, using a custom genotyping chip (iSelect) that was described in the 2013 IGAP GWAS meta-analysis (11,632 variants).^51^ Stage 3A (4,930 cases and 6,736 controls) involved replication for variants that showed suggestive association with late-onset AD (*P* < 5 × 10^-7^) in the meta-analysis of Stages 1 and 2, and variants that were genome-wide significant (*P* < 5 × 10^-8^) in the previous IGAP GWAS but not in the meta-analysis of Stages 1 and 2 (11 variants in total). Finally, variants with minor allele frequency (MAF) < 0.05 and *P* < 1 × 10^-5^ or MAF ≥ 0.05 and *P* < 5 × 10^-6^ in Stage 1 that were in regions not well-captured by the iSelect chip (33 variants in total) were selected for follow-up genotyping in Stage 3B (combined samples from Stage 2 and Stage 3A: 13,292 cases and 17,219 controls). The three stages had non-overlapping samples. The final sample consisted of 35,274 clinical and autopsy-documented cases of late-onset AD and 59,163 controls (94,437 individuals in total).

Standard quality control procedures were applied to each dataset prior to imputation, including the exclusion of individuals of non-European ancestry according to principal components analysis and those with a high degree of genetic relatedness. Individuals with low call rate and variants with low call rate were also removed from the analysis. The discovery datasets were then phased and imputed using the 1,000 Genomes Project reference panel (phase 1 integrated release 3, March 2012), using all reference population haplotypes for the imputation. Both common (MAF ≥ 0.01) and rare (MAF < 0.01) variants were sampled. The minimum imputation quality score required for inclusion was 0.4 for common variants and 0.7 for rare variants. Additionally, variants had to be present in at least 30% of cases and 30% of controls across all datasets to be retained in the analysis. A total of 9,456,058 common variants and 2,024,574 rare variants were selected for analysis after quality control.

**Multi-modal brain imaging in the UK Biobank**

Genetic association estimates with imaging-derived phenotypes were based on the largest-to-date release of combined genetic and multi-modal brain imaging data from the UK Biobank. The UK Biobank is a large prospective cohort study of approximately 500,000 individuals (aged 40–69 years at baseline recruitment), gathering extensive lifestyle, cognitive, physical, and biological measures (including genotyping), as well as data on ongoing health outcomes.^52^ An imaging extension was added to the existing UK Biobank study in 2016, aiming to acquire consistent multi-modal brain imaging data in 100,000 participants from the existing cohort by 2022.^53^ The early-2020 release of brain imaging samples from the UK Biobank contained data from approximately 40,000 participants across nearly 4,000 imaging-derived phenotypes. The imaging phenotypes span modalities that capture the anatomical and neuropathological structure of the brain (structural MRI), local microstructure of brain tissue (diffusion MRI), and brain activity (functional MRI).

**In-house GWAS of cortical macro- and micro-structure^54^**

The macro-structure metrics were based on T1-weighted structural images obtained from the UK Biobank in minimally processed form (application number 20904), which were further pre-processed using FreeSurfer software (version 6.0.1).^55^ The processing pipeline documentation and code is available on <https://github.com/ucam-department-of-psychiatry/UKB>. The micro-structure metrics were based on structural diffusion weighted imaging data, which were obtained in processed form from the UK Biobank. Estimation of the NODDI parameters (intracellular volume fraction and orientation dispersion index) from the minimally processed diffusion images was based on the Accelerated Microstructure Imaging via Convex Optimization (AMICO) processing pipeline.^56^ Across all imaging-derived phenotypes, the maximum number of participants and variants retained after quality control was 31,977 and 10,004,255, respectively. All genetic association estimates were adjusted for age, age^2^, sex, age × sex, age^2^ × sex, the first 40 genetic principal components, and the following imaging covariates: imaging center, mean framewise displacement, maximum framewise displacement, and the Euler Index.^57^

The analysis was restricted to individuals of self-reported White European ethnicity. Subject-level exclusion criteria included excessive genetic heterozygosity, non-matching genetic and reported sex, genotyping rate of less than 95%, and a deviation of more than $\pm$5 standard deviations from the means of the first two genetic principal components. Variant quality control involved the removal of SNPs with minor allele frequency (MAF) < 0.1%, SNPs missing in more than 5% of individuals, SNPs deviating from Hardy-Weinberg equilibrium (*P* < 1 × 10^-6^) and those with an imputation *R^2^* > 0.4 (for imputed SNPs). Prior to conducting the GWAS, all phenotypes were scaled to have a mean of 0 and a standard deviation of 1. Phenotypic outliers (i.e., individuals with scores above or below 5 standard deviations from the mean) were excluded from the analysis. Outliers were further removed by visually inspecting the histograms of all phenotypes and removing values above or below 5 median absolute deviations for phenotypes that were substantially skewed, primary the mean diffusivity phenotype.

**GWAS of white matter micro-structure by Zhao *et al.*^8^**

The GWAS of human brain white matter micro-structure was based on diffusion MRI data from 34,024 individuals of British ancestry in the UK Biobank. White matter microstructure was quantified using fractional anisotropy, mean diffusivity, and other metrics derived from diffusion MRI data using DTI models.^6^ Image processing was harmonized using the ENIGMA-DTI pipeline.^58,59^ Each of the DTI-derived metrics was estimated along 21 predefined cerebral white matter tracts (i.e., tract-averaged parameters) and across all 21 white matter tracts (i.e., global parameters). The discovery GWAS identified 151 genomic regions significantly associated with white matter microstructure (*P* < 2.3 × 10^-10^, corrected for the number of phenotypes studied), which together explained 41% of the variation in white matter microstructure.

**GWAS of hippocampal volumes and white matter hyperintensities volume by Smith *et al.*^60^**

Imaging-derived phenotypes were generated using the automated image processing pipeline developed by Alfaro-Almagro et al.^61^ on behalf of UK Biobank (<https://www.fmrib.ox.ac.uk/ukbiobank/fbp/>), which removes artifacts and aligns images across different individuals and imaging modalities. The GWAS was based on data from 33,224 participants (52.4% genetic females), with a mean (standard deviation) age of 64.28 (7.49) years. A total of 16,445,196 autosomal variants were retained in the analysis after applying quality control filters. All genetic association tests were adjusted for age, sex, 40 population genetic principal components, head size, imaging center, scanner table position, and scan date-related slow drifts.

Phenotypic outliers (i.e., greater than $\pm$ 6 median absolute deviation from the median) and individuals with missing data for 50 or more imaging-derived phenotypes were discarded from the analysis. Each imaging-derived phenotypes was quantile-normalized, resulting in a Gaussian distribution with a mean of 0 and a standard deviation of 1. Subject-level quality control filters included removing participants without recent UK ancestry (as determined by self-reported ancestry and genetic principal components threshold), as well as the exclusion of individuals based on genetic relatedness. Quality control filters for the inclusion of genetic variants included MAF ≥ 0.001, information score (INFO)^62^ ≥ 0.3, and a Hardy-Weinberg equilibrium *P* < 1 × 10^-7^.

### **Phenotypic correlations quality control pipeline**

Individual-level data for each imaging-derived phenotype was either obtained in processed form from the UK Biobank (for left and right hippocampal volume and total volume of white matter hyperintensities), or downloaded in minimally processed form from the UK Biobank and underwent the in-house imaging processing pipeline (documented on <https://github.com/ucam-department-of-psychiatry/UKB>). Prior to calculating the pair-wise phenotypic correlations, all phenotypes were scaled to have a mean of 0 and a standard deviation of 1. We removed any individuals who scored above or below 5 standard deviations from the mean for all phenotypes, as these outliers would skew the phenotypic scores and affect the pair-wise correlations between the imaging phenotypes. Additionally, we visually inspected histograms of all phenotypes and further removed any outliers above or below 5 median absolute deviations for phenotypes with substantial skew, primarily for mean diffusivity. We then calculated pairwise Pearson’s correlations between the 15 imaging-derived phenotypes (Supplementary Fig. 1).


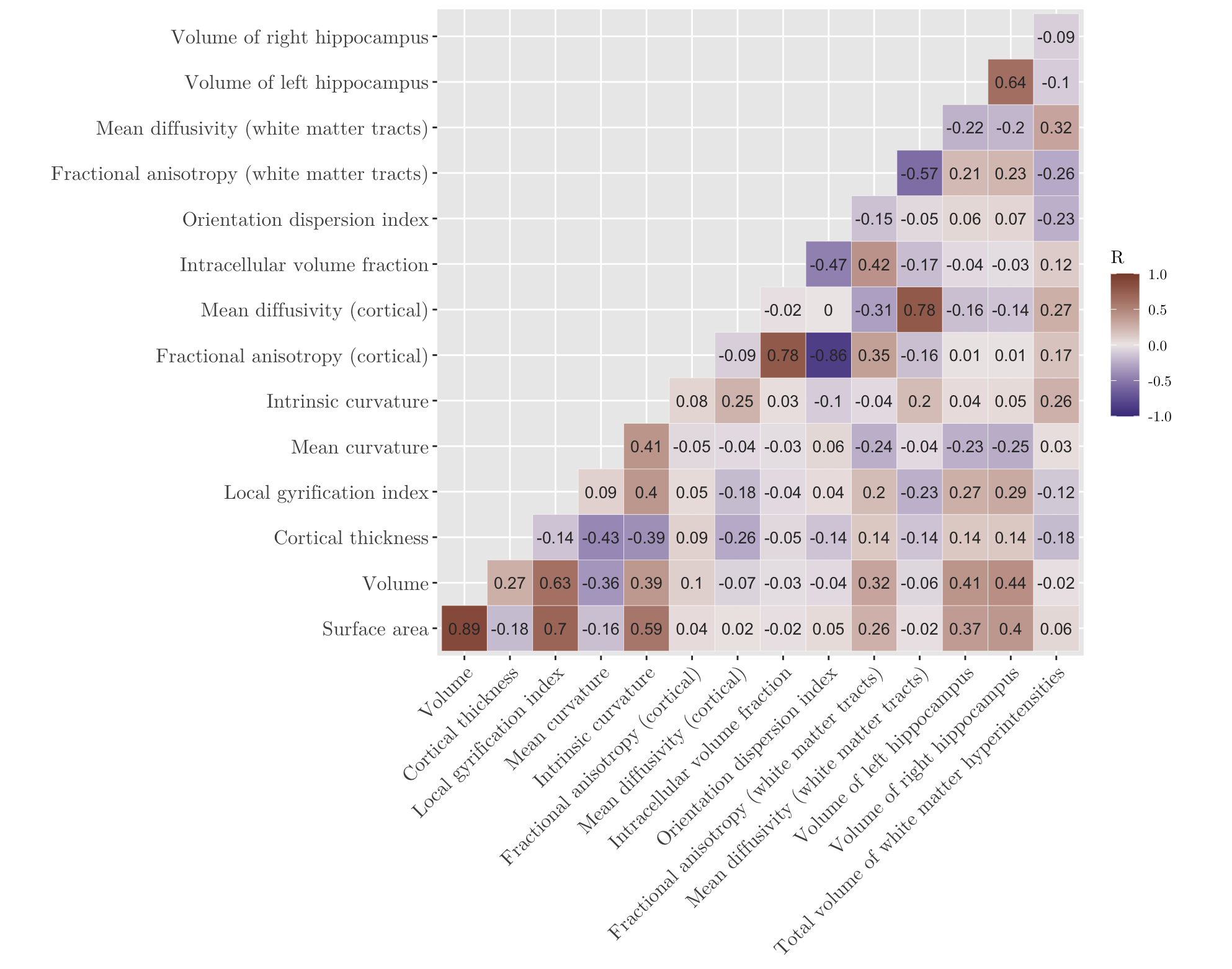


**Supplementary Fig. 1 Matrix of Pearson’s correlation coefficients between each pair of imaging-derived phenotypes.**

### **Robust Mendelian randomization methods**

#### **Weighted Median MR**

The median method is a consensus method, in that it derives its causal effect estimate from a summarised measure of the distribution of the variant-specific ratio estimates.^63^ The simple version of the median method takes the median of the variant-specific ratio estimates as its estimate of the causal effect, with all variants receiving equal weights.^63^ Provided that at least 50% of the genetic variants are valid instrumental variables (IVs), the median of the variant-specific causal estimates will tend towards the true causal parameter, and the estimate from this method will be unbiased (i.e., the “majority valid” assumption).^63^ However, the simple median estimator will be inefficient if there is considerable variation in the precision of the individual ratio estimates.^64^ The current analysis uses the weighted version of the median method,^64^ in which genetic variants receive standardised weights corresponding to the inverse of the variance of their ratio estimate, such that variants with more precise causal estimates receive higher weights. The weighted median estimator is then calculated as the median of the empirical distribution of the individual ratio estimates. This method can provide consistent causal effect estimates even when up to 50% of the weights contributing to the analysis come from invalid IVs.^64^ Although the weighted median estimator will be influenced by invalid IVs in a finite sample, the degree of bias will be far less than for the IVW method.^65^ Furthermore, since the median of a distribution is not influenced by the magnitude of extreme values, weighted median MR is naturally robust to outliers. However, this method is sensitive to the addition or removal of genetic variants from the analysis, as well as changes in the ratio estimates of individual variants contributing to the analysis. Furthermore, the estimate from weighted median MR may be less efficient (i.e., larger standard errors and lower power) than methods that derive their causal estimate from all variants.^63^

#### **Contamination mixture method**

The contamination mixture method^66^ is useful in two broad contexts. First, assuming that there is a single causal effect of the exposure on the outcome, this method can provide a robust and efficient estimate of the overall causal effect in the presence of some invalid IVs. Second, it can identify clusters of genetic variants with similar causal estimates, which may provide evidence for distinct causal mechanisms by which the exposure affects the outcome.^66^ The contamination mixture method can provide a robust and efficient estimate of the overall causal effect under the plurality valid assumption, which states that out of all the different values taken by the variant-specific ratio estimates, the value that is taken by the largest group of variants is the true causal effect of the exposure on the outcome.^63^

The method is performed by constructing a likelihood function from the variant-specific ratio estimates. If a genetic variant is a valid instrument, then its ratio estimate is assumed to follow a normal distribution centred around the true causal effect value. For genetic variants that are invalid instruments, the method assumes that their causal estimates are normally distributed about zero with a large variance. The likelihood function is then maximised by considering different values of the causal effect parameter (*θ* ) in turn and finding the configuration of valid and invalid instruments that maximises the likelihood function for a given value of *θ*. This is a profile likelihood approach to maximisation as it is based on constructing a one-dimensional function of the causal effect parameter.^66^ The value of *θ* that maximises the profile likelihood is taken as the point estimate of the causal effect, and 95% confidence intervals are constructed using the likelihood function (i.e., by finding the range of values of *θ* for which twice the difference between the log-likelihood at that value and the log-likelihood at the maximum likelihood estimator falls below the 95^th^ percentile of a chi-squared distribution with one degree of freedom).^66^ Confidence intervals from the contamination mixture method are typically not symmetric and may comprise several disjointed ranges of values. This would occur if there were multiple groups of variants each supporting distinct causal effect values and there was uncertainty regarding which group has more weight of evidence.^66^

The contamination mixture method is robust to outliers^63^ and does not make any strict modelling assumptions, and so is less likely to be affected by violations of such assumptions. However, it can be sensitive to specification of the variance parameter for the distribution of invalid instruments, as well as the addition or removal of genetic variants from the analysis.^63^

#### **MR-Egger**

The MR-Egger method is an adaptation of Egger regression, which can be used to test and correct for bias arising from directional pleiotropy (i.e., the average pleiotropic effects of genetic variants not equalling zero).^67^ MR-Egger is implemented similarly to the IVW method, except for the inclusion of an intercept term in the regression model. The slope coefficient from Egger regression provides the MR-Egger estimate of the causal effect, and the intercept term represents the average pleiotropic effect across all variants.^67^ The test of the null hypothesis that the MR-Egger estimate does not differ from zero is referred to as the MR-Egger causal test.^68^ Provided that the pleiotropic effect of each genetic variant on the outcome is independent of the association of the variant with the exposure (referred to as the Instrument Strength Independent of Direct Effect – ‘InSIDE’ – assumption), the MR Egger method can provide consistent causal effect estimates even if all of the genetic variants included in the analysis are invalid instruments.^67^ In addition, given that the intercept term will differ from zero either when the average pleiotropic effect is not equal to zero, or when the ‘InSIDE’ assumption is violated, testing the intercept from the MR-Egger analysis can be used to assess whether the instrumental variable assumptions are violated. This test is referred to as the MR-Egger intercept test, with a non-zero intercept indicating that the IVW estimate is biased.^68^

There are a number of issues relating to the MR-Egger method. First, the causal estimate from the MR-Egger method will always have a larger standard error than that from the IVW analysis, and the difference in precision can sometimes be substantial.^65^ In the IVW method, the precision of the causal estimate is dependent on the proportion of variance in the exposure that is accounted for by the genetic variants.^69^ However, the precision of the MR-Egger estimate additionally depends on the heterogeneity between genetic associations with the exposure.^70^ Therefore, if several genetic variants have similar associations with the exposure, the MR-Egger intercept and causal estimate will have low precision and wide confidence intervals.^68^

Second, the MR-Egger causal estimate and intercept test can be highly sensitive to the addition or removal of genetic variants from the analysis.^68^ Finally, in the case of pleiotropy, the assessment and estimation of the causal effect using MR-Egger, as well as the interpretation of the intercept as the average pleiotropic effect across all variants, require the InSIDE assumption to be satisfied. However, this assumption is generally implausible when several genetic variants have pleiotropic effects that act via confounders of the exposure-outcome association. This is because an increase in the association between the genetic variant and the confounder will lead to increases in its association with both the exposure and the outcome. Given that the instrument strengths and pleiotropic effects of such variants both depend on the magnitude of the genetic variant-confounder association, they will be strongly correlated, leading to violation of the InSIDE assumption and bias in the MR-estimate.^68^

#### **MR-PRESSO**

The Mendelian randomization pleiotropy residual sum and outlier (MR-PRESSO) method^71^ is an outlier-robust method which identifies and removes horizontal pleiotropic genetic variants with outlying ratio estimates from the analysis. Provided that the assumptions of the IVW method are met for the genetic variants not identified as outliers, this method can provide consistent causal effect estimates.^65^

The MR-PRESSO method has the following three components: (1) the MR-PRESSO global test, which detects the presence of horizontal pleiotropy; (2) the MR-PRESSO outlier test, which corrects for horizontal pleiotropy by removing outliers; and (3) the MR-PRESSO distortion test, which tests for significant differences between the causal estimates before and after the removal of horizontal pleiotropic outlying variants.^71^ The MR-PRESSO method uses standard IVW meta-analysis to compute the slope of the regression line (representing the causal effect of the exposure on the outcome) while excluding each variant in turn. For each variant, the observed residual sum of squares (RSS_obs_) is computed as the squared deviation between the observed effect of the variant on the outcome and the effect size predicted by the regression line computed without that variant. A distribution of K expected RSS (RSS_exp_) is then computed for each variant by drawing K random pairs of exposure and outcome effect sizes from two Gaussian distributions based on the predicted effect sizes and standard errors using the regression line computed without the variant. The MR-PRESSO global test detects the presence of overall horizontal pleiotropy amongst all variants by comparing the sum of the RSS_obs_ across all variants to the distribution of the K sum of RSS_exp_ (i.e., by comparing the observed RSS of all variants with the expected RSS under the null hypothesis of no horizontal pleiotropy). The MR-PRESSO outlier test can be used to detect specific horizontal pleiotropic outlier variants by comparing the RSS_obs_ for each variant to the distribution of the K RSS_exp_ for that variant. Finally, the MR-PRESSO distortion test assesses whether there is a significant difference between the causal estimate obtained before and after the removal of outlier variants detected by the MR-PRESSO outlier test.

The MR-PRESSO method is efficient when the analysis includes a small number of outlying variants with heterogeneous estimates, as these will be removed from the analysis and the overall causal estimate will not be affected by them.^65^ However, when there are several pleiotropic genetic variants, this method can have a very high false positive rate.^63^ Furthermore, if a substantial proportion of the genetic variants are removed from the analysis, homogeneity amongst the ratio estimates from the remaining variants can give a false impression of over-precision in the overall causal estimate. However, this would not necessarily provide strong evidence in support of a causal association, as a large number of variants have been excluded from the analysis.^65^

### **Assumptions of univariable and multivariable Mendelian randomization**

For a genetic variant to be a valid instrumental variable in a univariable MR model:^65^

1. The variant must be associated with the exposure of interest.
2. The variant must not be associated with any measured or unmeasured confounders of the exposure-outcome association.
3. The variant must only affect the outcome indirectly via the hypothesized causal pathway through the exposure (i.e., no direct pathway from the genetic variant to the outcome).

For a genetic variant to be a valid instrumental variable in a multivariable MR model:^72^

1. The variant must be associated with at least one of the exposures.
2. The variant must not be associated with any confounders of any of the exposure-outcome associations.
3. The variant must only affect the outcome indirectly via one or more of the exposures included in the analysis model.

A directed acyclic graph comparing the assumptions of univariable and multivariable Mendelian randomization is presented in Supplementary Fig. 2.


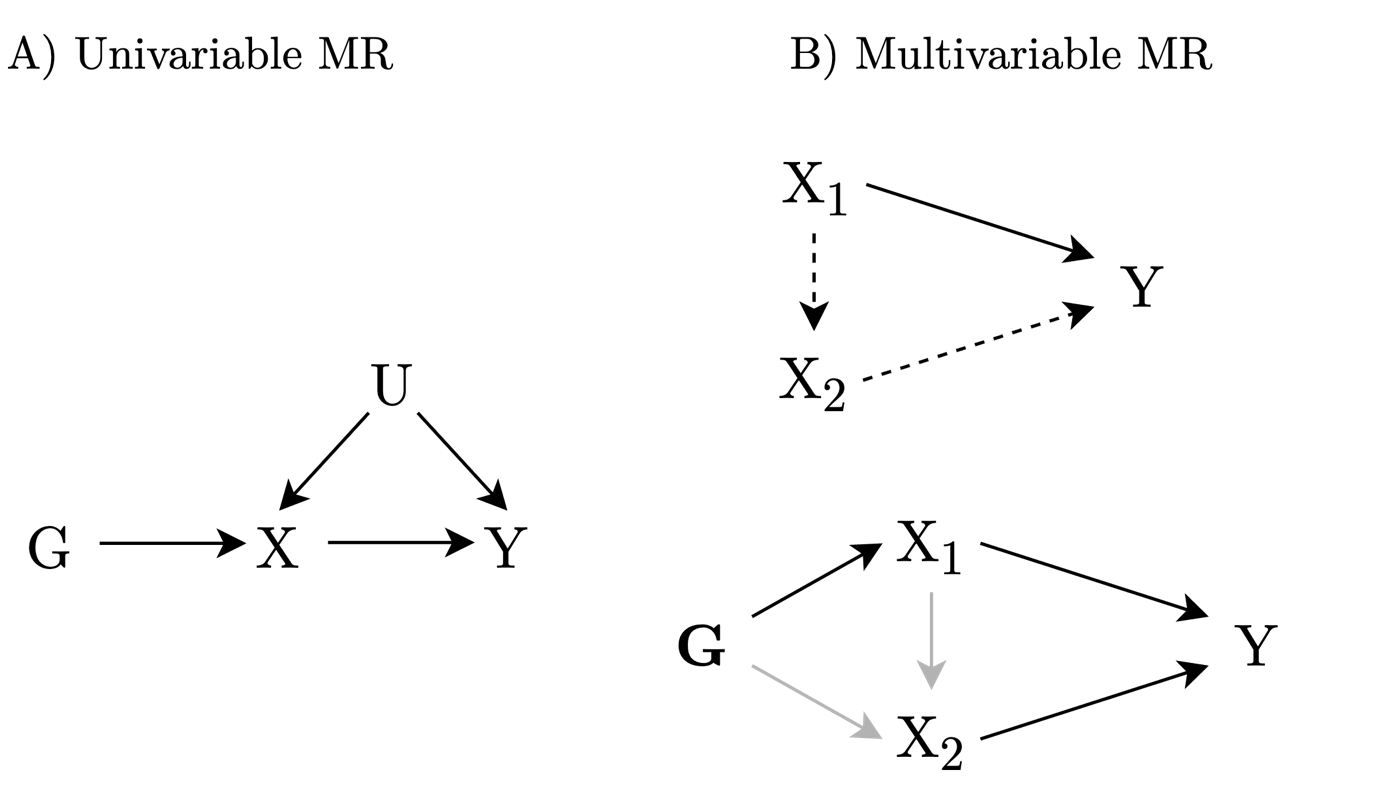


**Supplementary Fig. 2 Assumptions of univariable and multivariable Mendelian randomization.** A) directed acyclic graph illustrating univariable Mendelian randomization for genetic variant G, exposure X, and outcome Y. U represents the unknown confounders of the association between X and Y. The presence of arrows between G and X indicates that the first instrumental variable assumption is satisfied. In addition, G is independent of the confounder U (assumption II), and there are no direct pathways from G to Y apart from that passing through X (assumption III). B) directed acyclic graphs illustrating direct and indirect causal effects in a multivariable Mendelian randomization model with two exposures (X_1_ and X_2_) and one outcome (Y). As shown in the top panel, the total effect of X_1_ on Y has two components: a direct effect of X_1_ on Y (the solid arrow) and an indirect effect of X_1_ on Y via X_2_ (the dashed arrow). Assuming X_1_ is the primary exposure of interest, the estimate from a univariable MR model excluding X_2_ represents the total causal effect of X_1_ on Y. The estimates from a multivariable MR model including both X_1_ and X_2_ represent the direct effect of X_1_ on Y (i.e., not via X_2_) and the direct effect of X_2_ on Y (i.e., not via X_1_). A difference between the univariable and multivariable estimates of the effect of X_1_ on Y indicates that there is a causal effect of X_1_ on X_2_ (grey arrows), and that X_2_ is a mediator on the causal pathway from G to X_1_ to Y. Based on Burgess & Thompson.^65^ MR = Mendelian randomization.

### **Interpretation of results from the MR-Egger method**

In several of the analyses (Fig. 3, Supplementary Figs 6, 19, and 26), estimates from the MR-Egger method differ from the IVW analysis and the remaining robust MR methods. As has been discussed in detail by Burgess & Thompson^68^ and summarized above in the ‘Robust Mendelian randomization methods’ section, estimates from the MR-Egger method tend to have low precision, particularly when the magnitude of the variant-exposure associations is similar for several IVs, which can lead to the MR-Egger method having low power to detect a causal effect.

In the current study, where we observe MR-Egger results which are discordant with the other methods, the pattern of findings is generally consistent with this explanation (i.e., causal estimates from the MR-Egger method having a larger standard error). For instance, in several panels of Supplementary Fig. 6 and Supplementary Fig. 19, the MR-Egger method suggests a null causal effect, while estimates from the other methods suggest positive or negative causal effects. However, in each case, the MR-Egger point estimate is still in the same direction as the estimates from the IVW analysis and other robust methods, and confidence intervals around the MR-Egger estimate overlap with all other point estimates.

To quantify the similarity between the genetic variant-exposure associations and the potential impact of this on the performance of the MR-Egger method in the two-sample summary data context, Bowden et al.^70^ have proposed a heterogeneity measure from the meta-analysis literature (the $\text{I}_{\text{GX}}^{\text{ }\text{2}}$). The $\text{I}_{\text{GX}}^{\text{ }\text{2}}$ statistic quantifies the strength of the “No measurement error violation” assumption and provides a measure of instrument strength for the MR-Egger method, with instrument strength being defined as a collective property of all genetic variants included in the analysis.^70^ When the variant-exposure associations are sufficiently heterogeneous, the $\text{I}_{\text{GX}}^{\text{ }\text{2}}$ value will be close to 1, indicating that any bias in the MR-Egger estimate due to weak instruments is likely to be negligible. However, if the genetic association estimates with the exposure are generally similar in magnitude, the value of the $\text{I}_{\text{GX}}^{\text{ }\text{2}}$ statistic may be much smaller than 1, and MR-Egger regression will tend to underestimate the causal effect.^70^ Bowden et al.^70^ recommend that for $\text{I}_{\text{GX}}^{\text{ }\text{2}}$ values less than 0.9, inferences from the MR-Egger method should be interpreted with caution. As a post-hoc analysis, we calculated the $\text{I}_{\text{GX}}^{\text{ }\text{2}}$ statistic for all MR-Egger analyses. As can be seen in Supplementary Table 5, $\text{I}_{\text{GX}}^{\text{ }\text{2}}$ values were considerably smaller than 1 for all bidirectional analyses of education and brain structure phenotypes. This indicates that causal effect estimates from the MR-Egger approach exhibit weak instrument bias in all of these analyses, providing another explanation for the discordant results between MR-Egger and the other methods.

### **Sensitivity analysis using rate-of-change measures of brain structure from the GWAS of Brouwer et al. (2022)^73^**

During the review process, in relation to the Referees’ comments and a recent GWAS on longitudinal brain changes^73^ being published, we conducted additional sensitivity analyses using the newly available longitudinal brain change summary statistics from the Brouwer et al.^73^ GWAS. The longitudinal GWAS came from the ENIGMA consortium, and included 15,000 individuals from 40 longitudinal cohorts including participants aged 4 to 99 years. The brain phenotypes in the Brouwer et al.^73^ GWAS mostly differed from the ones used in our main analyses, but we took the opportunity to examine three phenotypes from this GWAS which were closely related to the measures we used: annual rate of change of cortical grey matter volume, annual rate of change of hippocampal volume, and annual rate of change of cortical surface area (measured at the grey /white matter boundary). The GWAS assumed a static effect of genetic variants across the lifespan. Due to the modest (by GWAS standards) sample size of this dataset, there were few, if any, instruments available for Mendelian randomization analysis at the rigorous threshold of *P* < 5 × 10^-8^. Therefore, in order to conduct Mendelian randomization analyses, we relaxed the *P*-value inclusion criterion threshold to *P* < 5 × 10^-6^. This, especially in combination with the small number of instruments used, means that the results should be viewed as preliminary and interpreted with caution.

The results of our additional Mendelian randomization analyses using the rates of change in brain metrics are shown in Supplementary Table 7. Most analyses did not demonstrate any significant associations. However, there was evidence of a strong association between genetically-predicted Alzheimer’s disease and rate of change of hippocampal volume. Here, a 2-fold increase in the odds of genetically-predicted Alzheimer’s disease was associated with an approximately 4 mm^3^/year reduction in the rate-of-change of hippocampal volume (β = -4.18, 95% CI -6.69, -1.66), which could either mean slower growth (e.g., from 5 mm^3^/year to 1 mm^3^/year) or faster shrinkage (e.g., from -1 mm^3^/year to -5 mm^3^/year).

We also noted a significant, although very small, association between genetically-predicted rate-of-change of hippocampal volume and Alzheimer’s disease, with a 1 mm^3^/year increase in genetically-predicted change rate of hippocampal volume associated with a 0.5% reduction in the odds of Alzheimer’s disease (OR = 0.995, 95% CI 0.991, 0.999). This sensitivity analysis was based on three genetic instruments that met our relaxed *P*-value threshold but not standard GWAS significance criteria. If this finding is replicated in future analyses with stronger genetic instruments (from larger GWAS), then it would represent a different result from our cross-sectional findings. Our cross-sectional analyes did not show evidence of association between genetically-predicted hippocampal volume and Alzheimer’s disease risk, but this longitudinal analysis hints at a possible (small) association between genetically-proxied hippocampal volume change rate and Alzheimer’s disease risk.

**Supplementary Table 1 Power calculations for bidirectional univariable Mendelian randomization analyses.**

| Hypothesized direction | | | |  | Reverse direction | | | |
| --- | --- | --- | --- | --- | --- | --- | --- | --- |
| Exposure | **Outcome** | **Proportion of variance in the exposure explained by the instrument (*R^2^*)** | **Minimum causal effect detectable with 80% power** |  | **Exposure** | **Outcome** | **Proportion of variance in the exposure explained by the instrument (*R^2^*)** | **Minimum causal effect detectable with 80% power** |
| EA | LOAD | 0.020 | OR ≤ 0.85 \| OR ≥ 1.18 |  | LOAD | EA | 0.458 | \|β\| ≥ 0.005 |
|  |  |  |  |  |  |  |  |  |
| EA | SA | 0.022 | \|β\| ≥ 0.14 |  | SA | EA | 0.027 | \|β\| ≥ 0.02 |
| EA | Vol | 0.022 | \|β\| ≥ 0.14 |  | Vol | EA | 0.021 | \|β\| ≥ 0.03 |
| EA | CT | 0.022 | \|β\| ≥ 0.14 |  | CT | EA | 0.021 | \|β\| ≥ 0.03 |
| EA | LGI | 0.022 | \|β\| ≥ 0.14 |  | LGI | EA | 0.018 | \|β\| ≥ 0.03 |
| EA | MC | 0.022 | \|β\| ≥ 0.14 |  | MC | EA | 0.030 | \|β\| ≥ 0.02 |
| EA | IC | 0.022 | \|β\| ≥ 0.14 |  | IC | EA | 0.010 | \|β\| ≥ 0.04 |
| EA | FA (cortex) | 0.022 | \|β\| ≥ 0.14 |  | FA (cortex) | EA | – | – |
| EA | MD (cortex) | 0.022 | \|β\| ≥ 0.14 |  | MD (cortex) | EA | 0.004 | \|β\| ≥ 0.06 |
| EA | ICVF | 0.022 | \|β\| ≥ 0.14 |  | ICVF | EA | 0.006 | \|β\| ≥ 0.05 |
| EA | ODI | 0.022 | \|β\| ≥ 0.14 |  | ODI | EA | 0.005 | \|β\| ≥ 0.06 |
| EA | FA (WM tracts) | 0.022 | \|β\| ≥ 0.13 |  | FA (WM tracts) | EA | 0.029 | \|β\| ≥ 0.02 |
| EA | MD (WM tracts) | 0.022 | \|β\| ≥ 0.13 |  | MD (WM tracts) | EA | 0.031 | \|β\| ≥ 0.02 |
| EA | HC Vol (left) | 0.022 | \|β\| ≥ 0.13 |  | HC Vol (left) | EA | 0.011 | \|β\| ≥ 0.04 |
| EA | HC Vol (right) | 0.022 | \|β\| ≥ 0.13 |  | HC Vol (right) | EA | 0.011 | \|β\| ≥ 0.04 |
| EA | WMH Vol | 0.022 | \|β\| ≥ 0.13 |  | WMH Vol | EA | 0.039 | \|β\| ≥ 0.02 |
|  |  |  |  |  |  |  |  |  |
| SA | LOAD | 0.026 | OR ≤ 0.83 \| OR ≥ 1.21 |  | LOAD | SA | 0.429 | \|β\| ≥ 0.03 |
| Vol | LOAD | 0.020 | OR ≤ 0.81 \| OR ≥ 1.24 |  | LOAD | Vol | 0.429 | \|β\| ≥ 0.03 |
| CT | LOAD | 0.019 | OR ≤ 0.81 \| OR ≥ 1.24 |  | LOAD | CT | 0.429 | \|β\| ≥ 0.03 |
| LGI | LOAD | 0.017 | OR ≤ 0.79 \| OR ≥ 1.26 |  | LOAD | LGI | 0.443 | \|β\| ≥ 0.03 |
| MC | LOAD | 0.026 | OR ≤ 0.83 \| OR ≥ 1.20 |  | LOAD | MC | 0.429 | \|β\| ≥ 0.03 |
| IC | LOAD | 0.008 | OR ≤ 0.72 \| OR ≥ 1.38 |  | LOAD | IC | 0.429 | \|β\| ≥ 0.03 |
| FA (cortex) | LOAD | – | – |  | LOAD | FA (cortex) | 0.429 | \|β\| ≥ 0.03 |
| MD (cortex) | LOAD | 0.003 | OR ≤ 0.58 \| OR ≥ 1.73 |  | LOAD | MD (cortex) | 0.442 | \|β\| ≥ 0.03 |
| ICVF | LOAD | 0.006 | OR ≤ 0.68 \| OR ≥ 1.48 |  | LOAD | ICVF | 0.429 | \|β\| ≥ 0.03 |
| ODI | LOAD | 0.005 | OR ≤ 0.67 \| OR ≥ 1.50 |  | LOAD | ODI | 0.429 | \|β\| ≥ 0.03 |
| FA (WM tracts) | LOAD | 0.027 | OR ≤ 0.83 \| OR ≥ 1.20 |  | LOAD | FA (WM tracts) | 0.429 | \|β\| ≥ 0.03 |
| MD (WM tracts) | LOAD | 0.028 | OR ≤ 0.84 \| OR ≥ 1.20 |  | LOAD | MD (WM tracts) | 0.429 | \|β\| ≥ 0.03 |
| HC Vol (left) | LOAD | 0.009 | OR ≤ 0.74 \| OR ≥ 1.36 |  | LOAD | HC Vol (left) | 0.460 | \|β\| ≥ 0.03 |
| HC Vol (right) | LOAD | 0.010 | OR ≤ 0.74 \| OR ≥ 1.35 |  | LOAD | HC Vol (right) | 0.460 | \|β\| ≥ 0.03 |
| WMH Vol | LOAD | 0.037 | OR ≤ 0.86 \| OR ≥ 1.17 |  | LOAD | WMH Vol | 0.460 | \|β\| ≥ 0.03 |

Power calculations were performed using the online calculator by Burgess^69^ (<http://sb452.shinyapps.io/power>). R^2^ was approximated using the following formula: $\text{R}^{\text{2}}\text{ }\text{= 2}\text{ }\text{β}_{\text{x}}^{\text{2}}\text{ }\text{MAF}\text{ }\text{(1-MAF)}$,^74^ where β_x_ is the genetic association estimate with the exposure, and MAF is the minor allele frequency. The minimum detectable causal effects are presented as odds ratios (per SD increase in genetically-predicted levels of the exposure) for the binary outcome (late-onset Alzheimer disease) and as beta coefficients (SD change in the outcome per SD increase in genetically-predicted levels of the exposure) for the continuous outcomes (imaging-derived phenotypes and educational attainment). EA = educational attainment; LOAD = late-onset Alzheimer’s disease; OR = odds ratio; SA = surface area; Vol = volume; CT = cortical thickness; LGI = local gyrification index; MC = mean curvature; IC = intrinsic curvature; FA = fractional anisotropy; MD = mean diffusivity; ICVF = intracellular volume fraction; ODI = orientation dispersion index; WM = white matter; HC = hippocampus; WMH = white matter hyperintensities; SD = standard deviation.

**Supplementary Table 2 Cochran’s Q tests for heterogeneity from MR-IVW analyses.**

| Exposure | Outcome | *Q* | df | *P*-value |
| --- | --- | --- | --- | --- |
| EA | LOAD | 333.35 | 331 | 0.453 |
| EA | SA | 1026.59 | 375 | <.001 |
| EA | Vol | 793.55 | 375 | <.001 |
| EA | LGI | 705.43 | 375 | <.001 |
| EA | IC | 872.14 | 375 | <.001 |
| EA | ICVF | 470.19 | 375 | <.001 |
| EA | WMH Vol | 574.26 | 376 | <.001 |
| SA | EA | 127.13 | 24 | <.001 |
| Vol | EA | 148.82 | 24 | <.001 |
| IC | EA | 165.74 | 19 | <.001 |
| LOAD | ODI | 38.60 | 27 | 0.069 |
| LOAD | MD (WM tracts) | 60.06 | 27 | <.001 |
| LOAD | HC Vol (left) | 26.63 | 27 | 0.484 |
| LOAD | HC Vol (right) | 38.35 | 27 | 0.072 |

MR = Mendelian randomization; IVW = inverse-variance weighted; df = degrees of freedom; EA = educational attainment; LOAD = late-onset Alzheimer’s disease; SA = surface area; Vol = volume; LGI = local gyrification index; IC = intrinsic curvature; ICVF = intracellular volume fraction; WMH = white matter hyperintensities; ODI = orientation dispersion index; MD = mean diffusivity; WM = white matter; HC = hippocampus.

**Supplementary Table 3 MR-Egger intercept tests for horizontal pleiotropy.**

| Exposure | Outcome | Egger intercept (95% CI) | *P*-value |
| --- | --- | --- | --- |
| EA | LOAD | 0.00 (0.00, 0.01) | 0.182 |
| EA | SA | 0.00 (-0.01, 0.00) | 0.223 |
| EA | Vol | 0.00 (-0.01, 0.00) | 0.255 |
| EA | LGI | 0.00 (0.00, 0.00) | 0.912 |
| EA | IC | 0.00 (0.00, 0.00) | 0.536 |
| EA | ICVF | 0.00 (0.00, 0.00) | 0.745 |
| EA | WMH Vol | 0.00 (0.00, 0.00) | 0.682 |
| SA | EA | 0.00 (0.00, 0.01) | 0.479 |
| Vol | EA | 0.00 (-0.01, 0.01) | 0.847 |
| IC | EA | 0.01 (0.00, 0.02) | 0.157 |
| LOAD | ODI | 0.00 (0.00, 0.01) | 0.348 |
| LOAD | MD (WM tracts) | -0.01 (-0.01, -0.00) | 0.020 |
| LOAD | HC Vol (left) | 0.00 (0.00, 0.01) | 0.442 |
| LOAD | HC Vol (right) | 0.00 (0.00, 0.01) | 0.467 |

MR = Mendelian randomization; CI = confidence interval; EA = educational attainment; LOAD = late-onset Alzheimer’s disease; SA = surface area; Vol = volume; LGI = local gyrification index; IC = intrinsic curvature; ICVF = intracellular volume fraction; WMH = white matter hyperintensities; ODI = orientation dispersion index; MD = mean diffusivity; WM = white matter; HC = hippocampus.

**Supplementary Table 4 Results of Steiger filtering sensitivity analyses.**

| Exposure | Outcome | N _SNPs_ ^a^ | | MR analysis (all SNPs) | | MR analysis (valid SNPs) | |
| --- | --- | --- | --- | --- | --- | --- | --- |
|  |  | **Total** | **Invalid** | **β _IVW_ (95% CI)** | ***P*-value** | **β _IVW_ (95% CI)** | ***P*-value** |
| EA | LOAD | 332 | 24 | -0.36 (-0.51, -0.22) | 9.98 × 10^-7^ | -0.33 (-0.48, -0.18) | 1.40 × 10^-5^ |
| EA | SA | 376 | 89 | 0.30 (0.20, 0.40) | 4.5 × 10^-9^ | 0.12 (0.05, 0.19) | 6.86 × 10^-4^ |
| EA | Vol | 376 | 87 | 0.29 (0.20, 0.37) | 3.31 × 10^-11^ | 0.14 (0.08, 0.21) | 1.92 × 10^-5^ |
| EA | LGI | 376 | 104 | 0.21 (0.11, 0.31) | 2.05 × 10^-5^ | 0.08 (0.00, 0.16) | 0.059 |
| EA | IC | 376 | 54 | 0.18 (0.11, 0.25) | 2.88 × 10^-7^ | 0.11 (0.06, 0.16) | 3.68 × 10^-5^ |
| EA | ICVF | 376 | 53 | -0.09 (-0.15, -0.03) | 0.006 | -0.07 (-0.13, -0.01) | 0.030 |
| EA | WMH Vol | 377 | 111 | -0.14 (-0.23, -0.05) | 0.003 | -0.05 (-0.13, 0.04) | 0.258 |
| SA | EA | 25 | 0 | 0.13 (0.10, 0.16) | 1.70 × 10^-14^ | 0.13 (0.10, 0.16) | 1.70 × 10^-14^ |
| Vol | EA | 25 | 0 | 0.15 (0.11, 0.19) | 2.00 × 10^-12^ | 0.15 (0.11, 0.19) | 2.00 × 10^-12^ |
| IC | EA | 20 | 0 | 0.12 (0.04, 0.19) | 0.001 | 0.12 (0.04, 0.19) | 0.001 |
| LOAD | ODI | 27 | 0 | -0.03 (-0.05, -0.01) | 0.002 | -0.03 (-0.05, -0.01) | 0.002 |
| LOAD | MD (WM tracts) | 27 | 0 | 0.03 (0.01, 0.05) | 0.008 | 0.03 (0.01, 0.05) | 0.008 |
| LOAD | HC Vol (left) | 27 | 0 | -0.03 (-0.05, -0.02) | 3.59 × 10^-5^ | -0.03 (-0.05, -0.02) | 3.59 × 10^-5^ |
| LOAD | HC Vol (right) | 27 | 0 | -0.03 (-0.05, -0.01) | 5.60 × 10^-4^ | -0.03 (-0.05, -0.01) | 5.60 × 10^-4^ |

^a^ Number of SNPs remaining after clumping for independence and data harmonization.

SNP = single nucleotide polymorphism; MR = Mendelian randomization; IVW = inverse-variance weighted; CI = confidence interval; EA = educational attainment; LOAD = late-onset Alzheimer’s disease; SA = surface area; Vol = volume; LGI = local gyrification index; IC = intrinsic curvature; ICVF = intracellular volume fraction; WMH = white matter hyperintensities; ODI = orientation dispersion index; MD = mean diffusivity; WM = white matter; HC = hippocampus.

**Supplementary Table 5** $\text{I}_{\text{GX}}^{\text{2}}$ **statistics to examine potential violation of the ‘No Measurement Error’ (NOME) Assumption for MR-Egger Analyses.**

| Exposure | Outcome | $\text{I}_{\text{GX}}^{\text{ 2}}$ of SNP-exposure associations |
| --- | --- | --- |
| EA | LOAD | 0.74 |
| EA | SA | 0.72 |
| EA | Vol | 0.72 |
| EA | LGI | 0.72 |
| EA | IC | 0.72 |
| EA | ICVF | 0.72 |
| EA | WMH Vol | 0.73 |
| SA | EA | 0.72 |
| Vol | EA | 0.36 |
| IC | EA | 0 |
| LOAD | ODI | 0.99 |
| LOAD | MD (WM tracts) | 0.99 |
| LOAD | HC Vol (left) | 0.99 |
| LOAD | HC Vol (right) | 0.99 |

MR = Mendelian randomization; SNP = single nucleotide polymorphism; EA = educational attainment; LOAD = late-onset Alzheimer’s disease; SA = surface area; Vol = volume; LGI = local gyrification index; IC = intrinsic curvature; ICVF = intracellular volume fraction; WMH = white matter hyperintensities; ODI = orientation dispersion index; MD = mean diffusivity; WM = white matter; HC = hippocampus.

**Supplementary Table 6 Sensitivity analysis showing Mendelian randomization estimates of the association of genetically-proxied cortical macro-structure, cortical micro-structure, and white matter micro-structure with Alzheimer’s disease risk, estimated with no adjustment (univariable analysis) and after adjustment for genetically-proxied cortical volume (multivariable analysis).**

|  | OR (95% CI) | |
| --- | --- | --- |
| Exposure | **Univariable MR _IVW_** | **Multivariable MR _IVW_** |
| Cortical macro-structure |  |  |
| LGI | 0.93 (0.79, 1.10) | 0.82 (0.62, 1.09) |
| MC | 0.91 (0.78, 1.07) | 0.92 (0.77, 1.11) |
| IC | 0.87 (0.65, 1.16) | 0.82 (0.53, 1.28) |
| Cortical micro-structure |  |  |
| FA (cortex) | NA | NA |
| MD (cortex) | 0.69 (0.46, 1.04) | 0.72 (0.44, 1.19) |
| ICVF | 1.11 (0.79, 1.56) | 1.17 (0.84, 1.62) |
| ODI | 1.03 (0.63, 1.69) | 1.02 (0.67, 1.55) |
| White matter micro-structure |  |  |
| FA (WM tracts) | 1.06 (0.88, 1.27) | 1.02 (0.85, 1.22 |
| MD (WM tracts) | 0.88 (0.74, 1.05) | 0.88 (0.74, 1.04) |

Note that the table only shows results for imaging-derived phenotypes which were not corrected for intracranial or total brain volume in the original GWAS. Surface area and cortical thickness were not included in these analyses as they are highly correlated with cortical volume. OR = odds ratio; CI = confidence interval; MR = Mendelian randomization; IVW = inverse-variance weighted; LGI = local gyrification index; MC = mean curvature; IC = intrinsic curvature; FA = fractional anisotropy; MD = mean diffusivity; ICVF = intracellular volume fraction; ODI = orientation dispersion index; WM = white matter; GWAS = genome-wide association study.

**Supplementary Table 7 Sensitivity analysis showing bidirectional Mendelian randomization results for rate-of-change measures of brain structure from the GWAS of Brouwer et al. (2022)^73^**

| Exposure (change rate) | Outcome | N _SNPs_ | Estimate (95% CI) | Exposure | Outcome (change rate) | N _SNPs_ | Estimate (95% CI) |
| --- | --- | --- | --- | --- | --- | --- | --- |
| Cortical GM | LOAD | 8 | 1.001 (0.995, 1.007) ^a^ | LOAD | Cortical GM | 20 | -35.77 (-142.56, 71.02) ^g^ |
| HC | LOAD | 3 | 0.995 (0.991, 0.999) ^b^ | LOAD | HC | 20 | -4.18 (-6.69, -1.66) ^g^ |
| SA | LOAD | 5 | 0.999 (0.996, 1.003) ^c^ | LOAD | SA | 20 | -4.21 (-23.23, 14.80) ^g^ |
| Cortical GM | EA | 8 | 9.60 × 10^-4^ (-1.47 × 10^-4^, 2.07 × 10^-3^) ^d^ | EA | Cortical GM | 364 | 114.82 (-213.15, 442.79) ^h^ |
| HC | EA | 4 | 2.27 × 10^-4^ (-4.80 × 10^-4^, 9.33 × 10^-4^) ^e^ | EA | HC | 364 | 2.90 (-5.52, 11.31) ^h^ |
| SA | EA | 4 | 9.35 × 10^-5^ (-7.12 × 10^-4^, 8.99 × 10^-4^) ^f^ | EA | SA | 363 | 17.35 (-43.95, 78.66) ^h^ |

^a^ Odds ratio of LOAD per 100 mm^3^/year increase in genetically-predicted change rate of cortical GM volume.

^b^ Odds ratio of LOAD per 1 mm^3^/year increase in genetically-predicted change rate of hippocampal volume.

^c^ Odds ratio of LOAD per 10 mm^2^/year increase in genetically-predicted change rate of surface area.

^d^ SD change in EA per 100 mm^3^/year increase in genetically-predicted change rate of cortical GM volume.

^e^ SD change in EA per 1 mm^3^/year increase in genetically-predicted change rate of hippocampal volume.

^f^ SD change in EA per 10 mm^2^/year increase in genetically-predicted change rate of surface area.

^g^ Unit change in rate-of-change measures of brain structures per doubling (2-fold increase) in the odds of genetically-predicted LOAD.

^h^ Unit change in rate-of-change measures of brain structures per SD increase in genetically-predicted EA.

GWAS = genome-wide association study; SNP = single nucleotide polymorphism; CI = confidence interval; GM = grey matter; LOAD = late-onset Alzheimer’s disease; HC = hippocampus; SA = surface area; EA = educational attainment; SD = standard deviation.


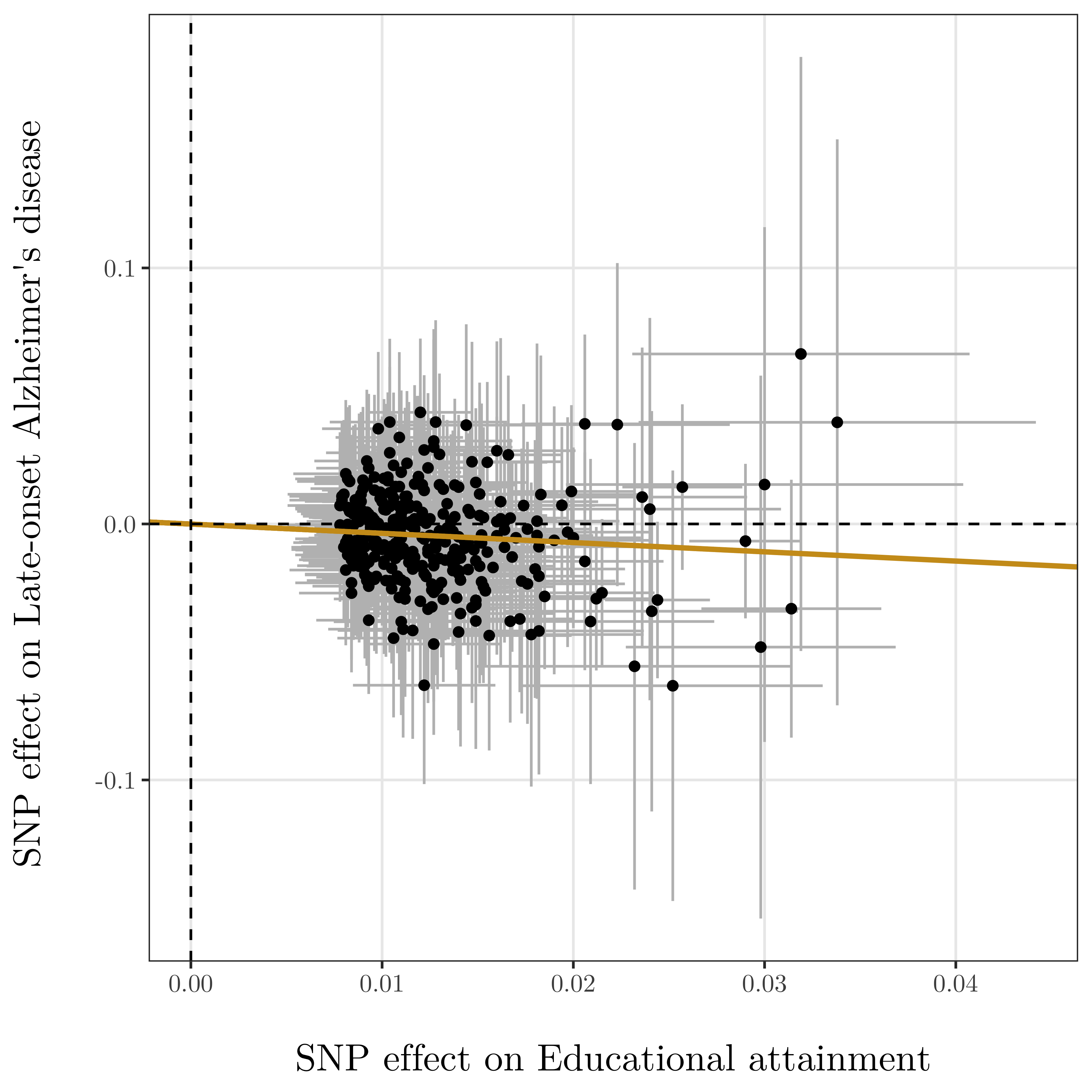


**Supplementary Fig. 3** Genetic associations with educational attainment (horizontal axis, standard deviation units) and with late-onset Alzheimer’s disease (vertical axis, log odds ratios) for 332 genetic variants associated with educational attainment at a genome-wide level of significance. Horizontal and vertical lines represent 95% confidence intervals for the genetic associations. The regression line through the origin represents the inverse-variance weighted Mendelian randomization estimate for the effect of educational attainment on late-onset Alzheimer’s disease risk.


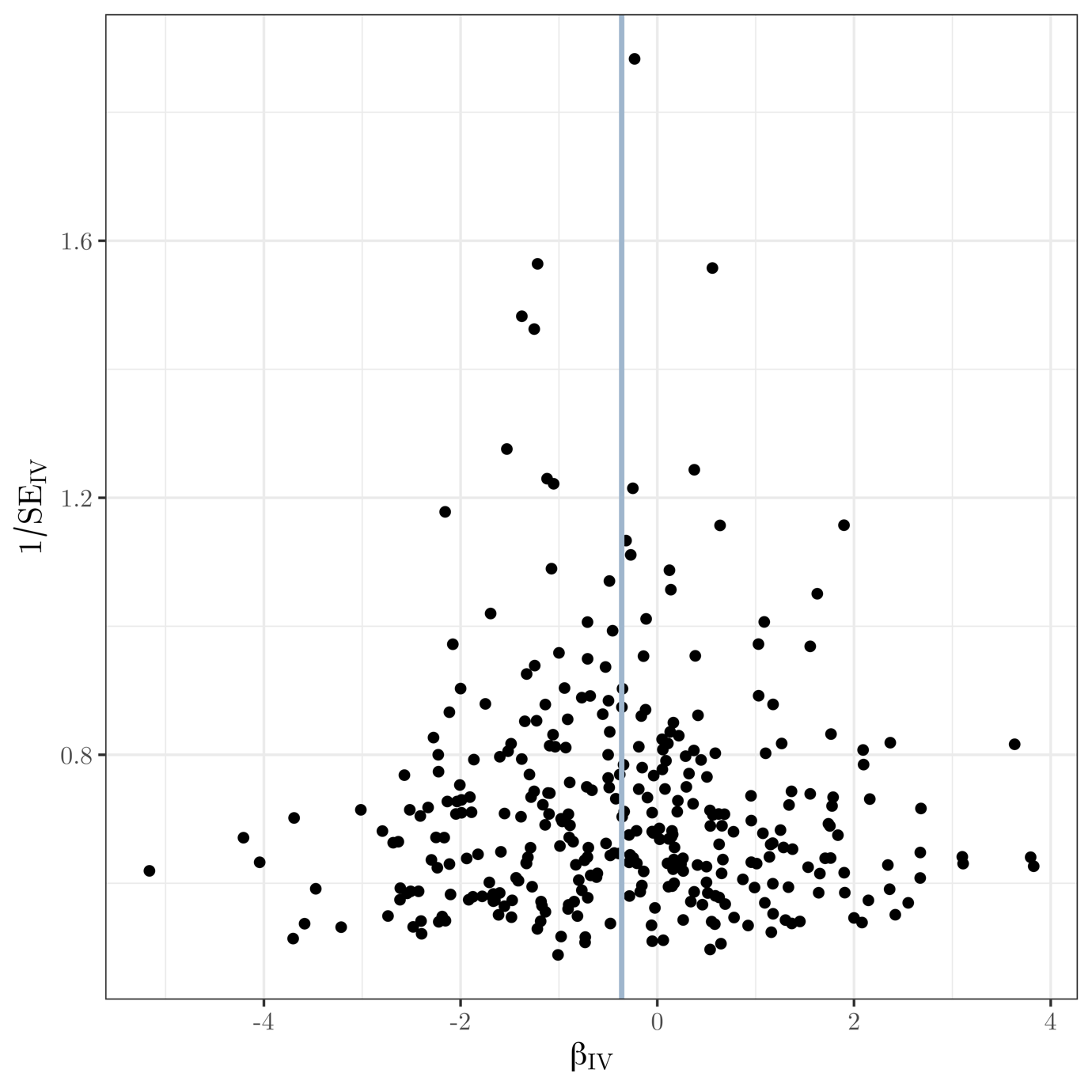


**Supplementary Fig. 4** Funnel plot of instrument precision against instrumental variable estimates for each genetic variant separately for Mendelian randomization analysis of educational attainment on late-onset AD risk. Solid vertical line is the (fixed-effect) inverse-variance weighted estimate.

**
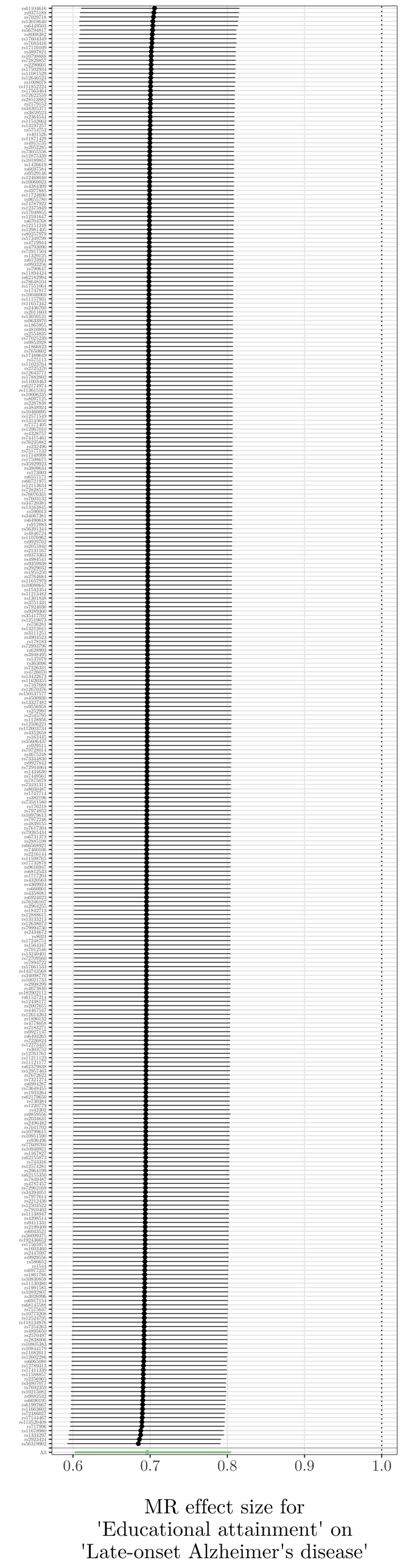
**

**Supplementary Fig. 5** Leave-one-out plot for MR analysis of educational attainment on late-onset AD risk.

| 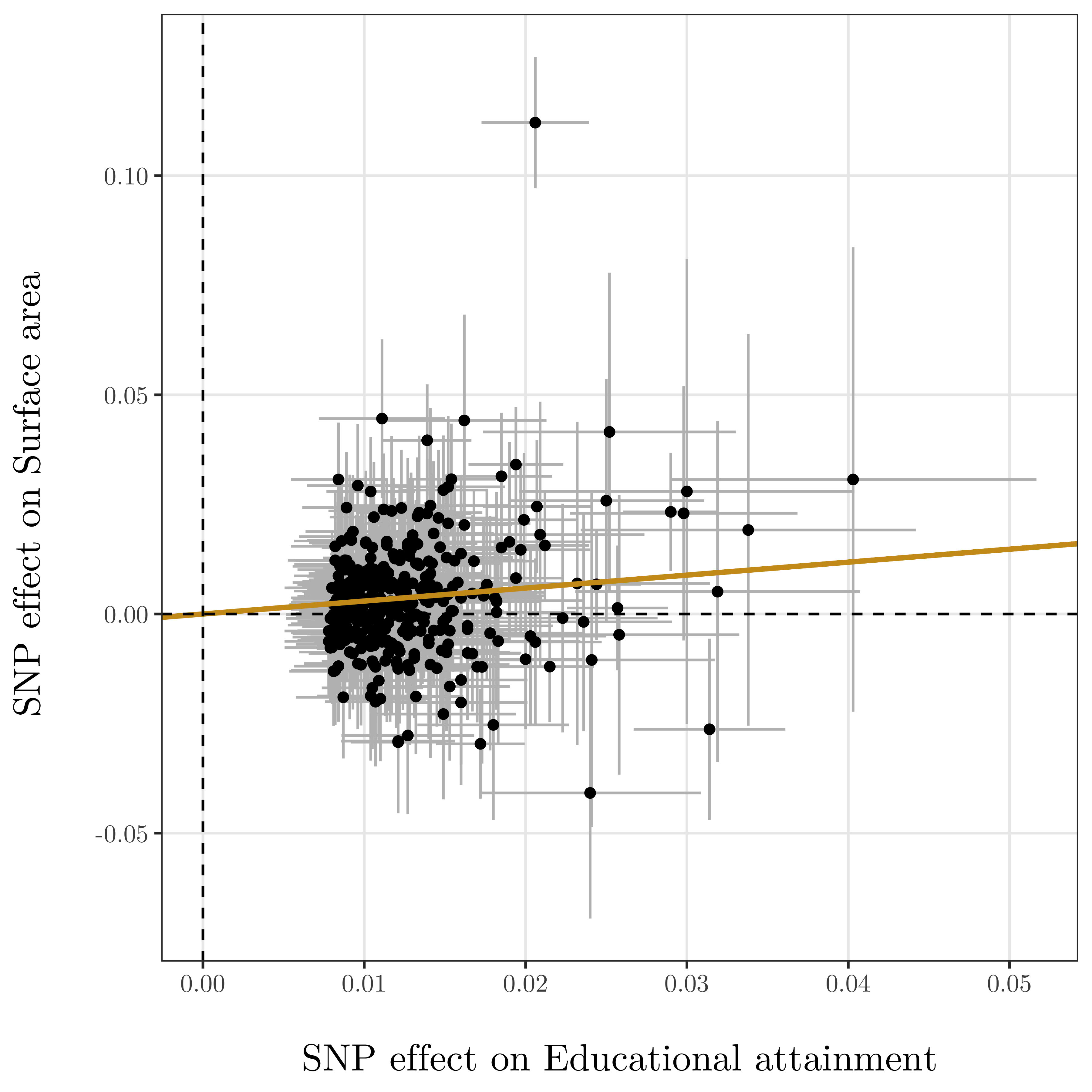 | 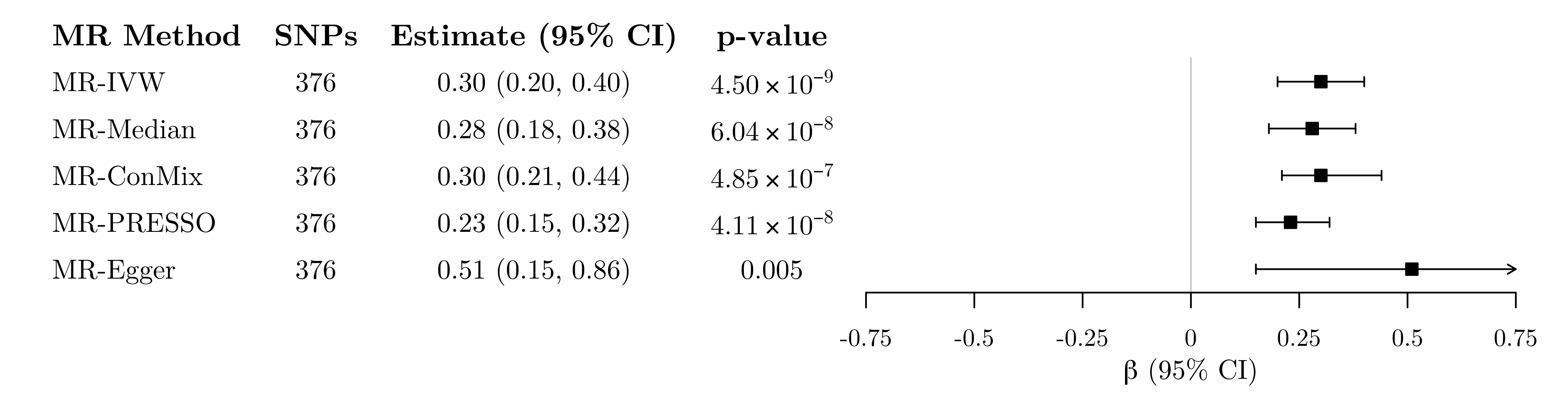 |
| --- | --- |
| 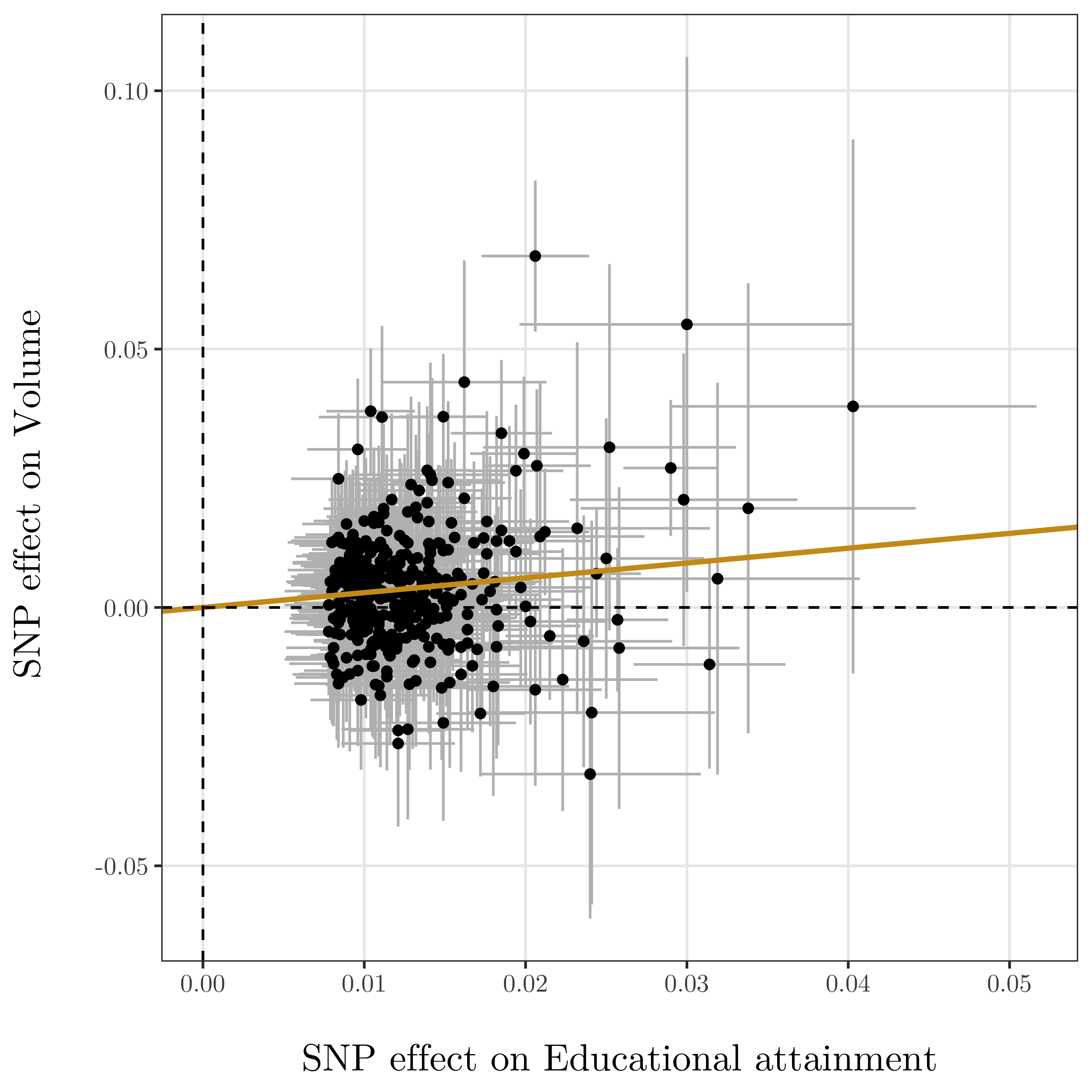 | 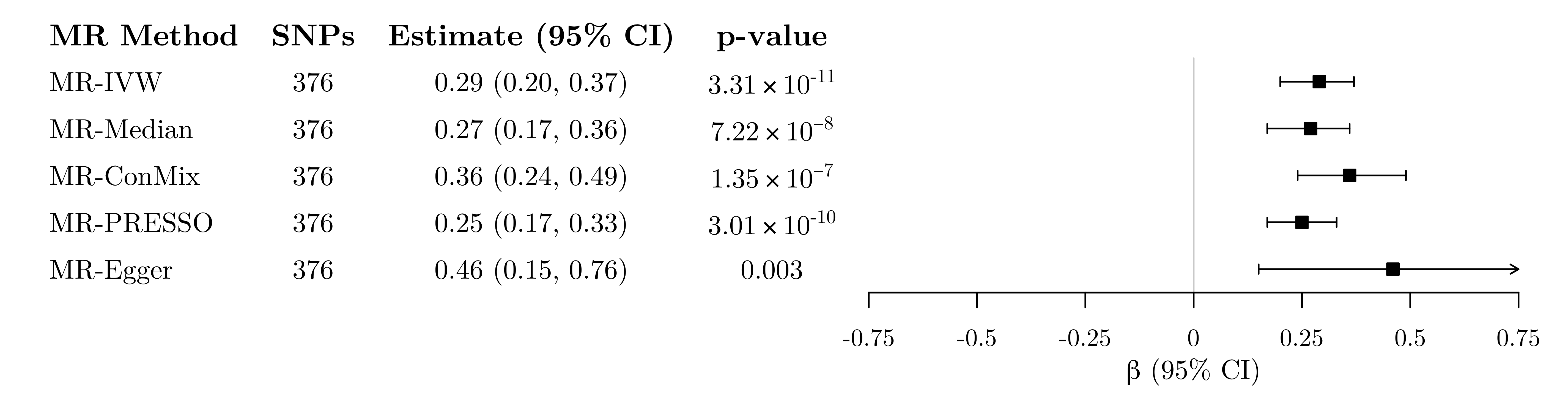 |

**Supplementary Fig. 6** Left: Genetic associations with educational attainment (horizontal axis, standard deviation units) and with imaging-derived phenotypes (vertical axis, standard deviation units) for 376 genetic variants associated with educational attainment at a genome-wide level of significance. Horizontal and vertical lines represent 95% confidence intervals for the genetic associations. The regression line through the origin represents the inverse-variance weighted Mendelian randomization estimate for the effect of educational attainment on each imaging-derived phenotype. Right: Mendelian randomization estimates of the association between genetically-proxied educational attainment and imaging-derived brain structure phenotypes. Estimates represent standard deviation change in the imaging phenotype per 1 standard deviation increase in genetically-predicted years of schooling (approximately 4.2 years).

| 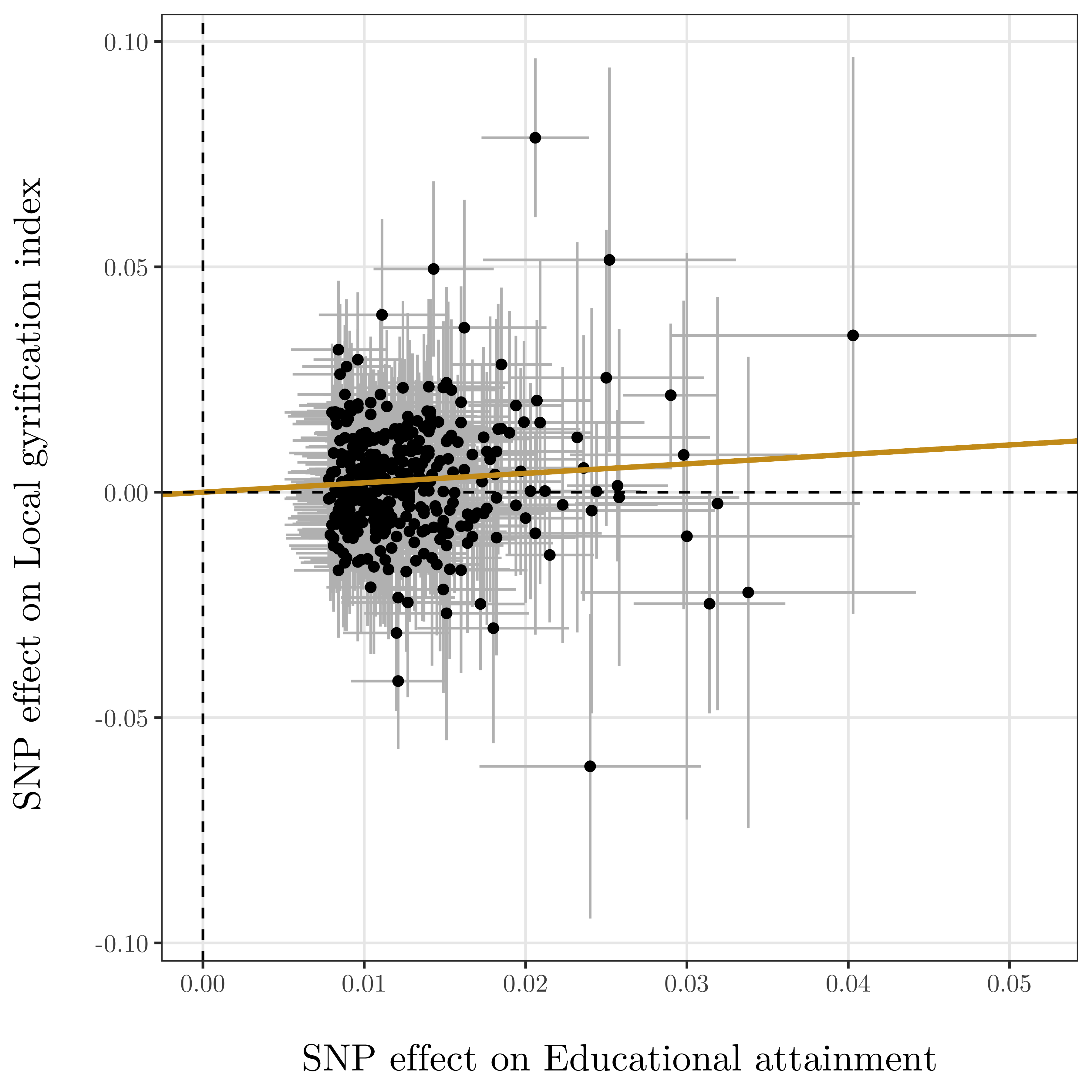 | 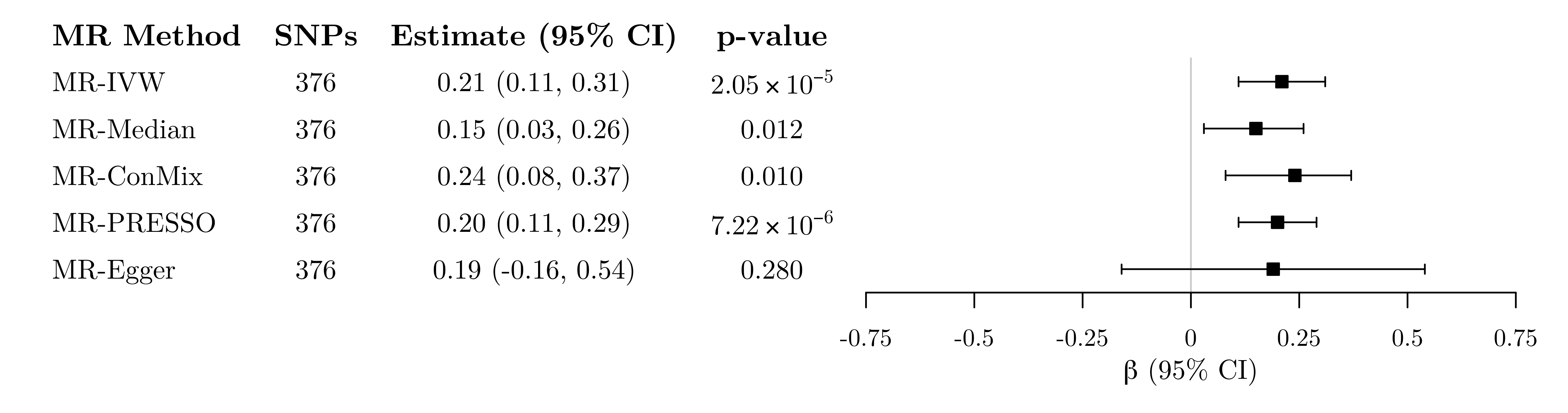 |
| --- | --- |
| 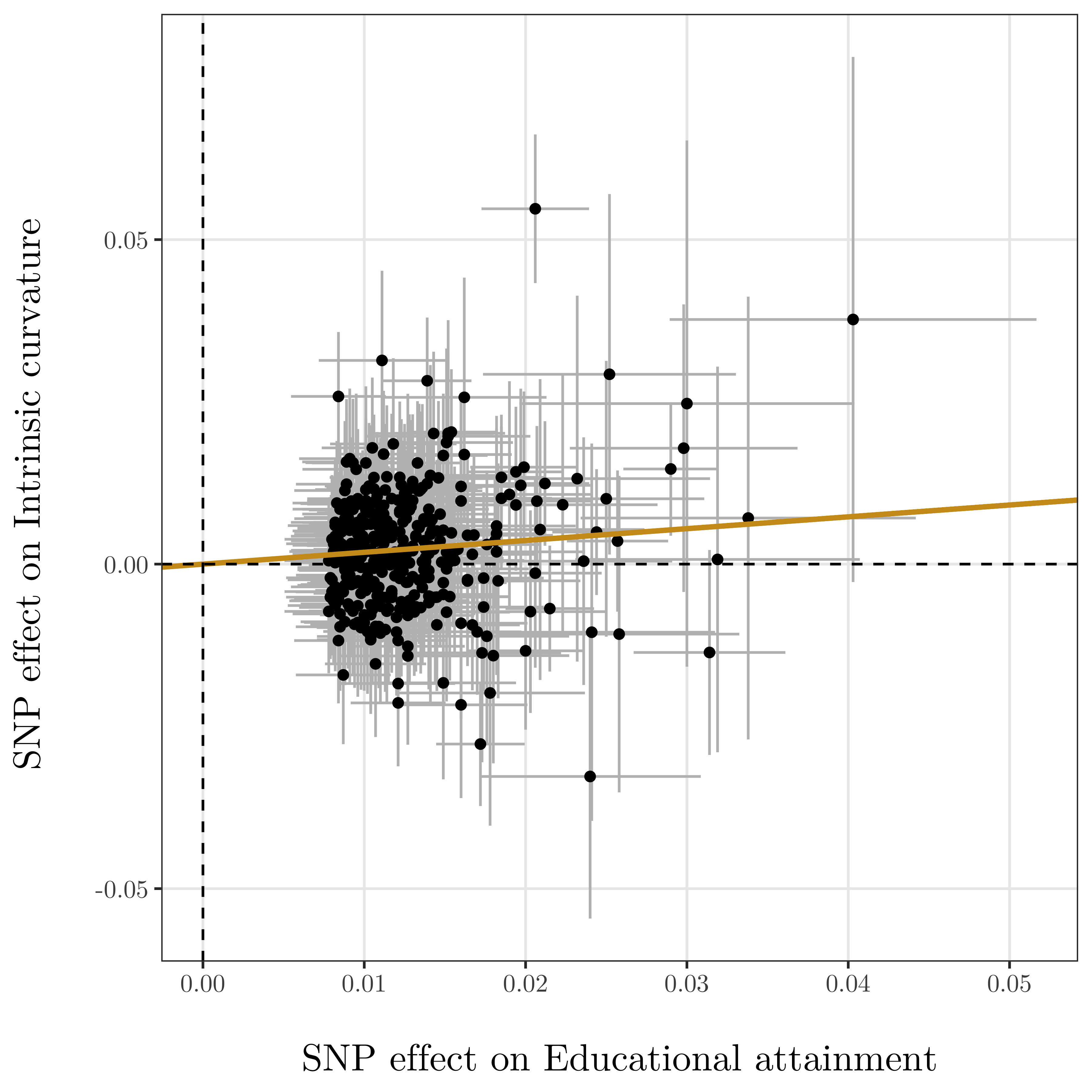 | 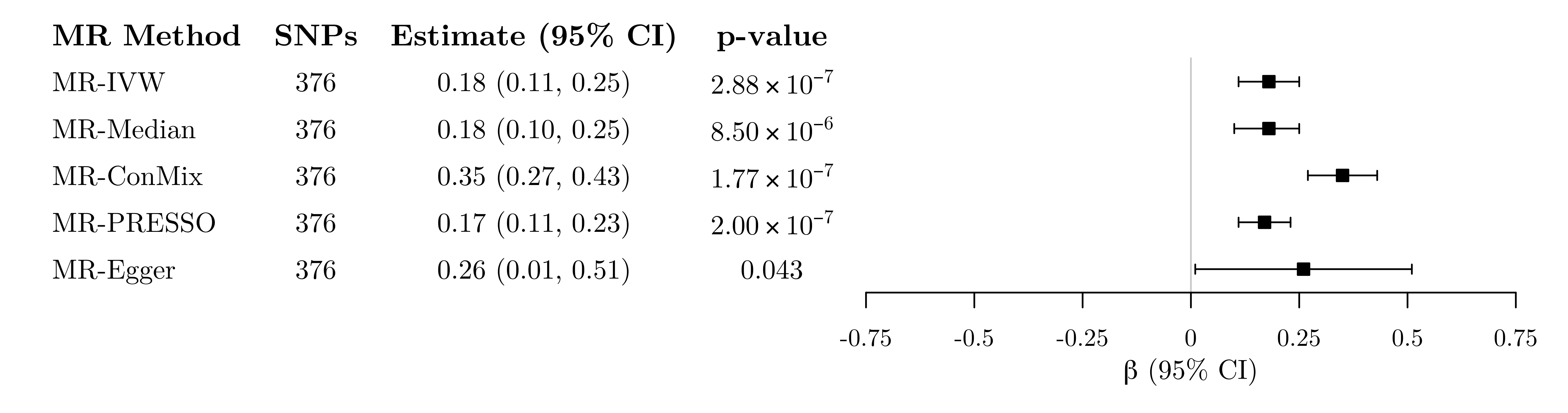 |

**Supplementary Fig. 6 (continued).** Left: Genetic associations with educational attainment (horizontal axis, standard deviation units) and with imaging-derived phenotypes (vertical axis, standard deviation units) for 376 genetic variants associated with educational attainment at a genome-wide level of significance. Horizontal and vertical lines represent 95% confidence intervals for the genetic associations. The regression line through the origin represents the inverse-variance weighted Mendelian randomization estimate for the effect of educational attainment on each imaging-derived phenotype. Right: Mendelian randomization estimates of the association between genetically-proxied educational attainment and imaging-derived brain structure phenotypes. Estimates represent standard deviation change in the imaging phenotype per 1 standard deviation increase in genetically-predicted years of schooling (approximately 4.2 years).

| 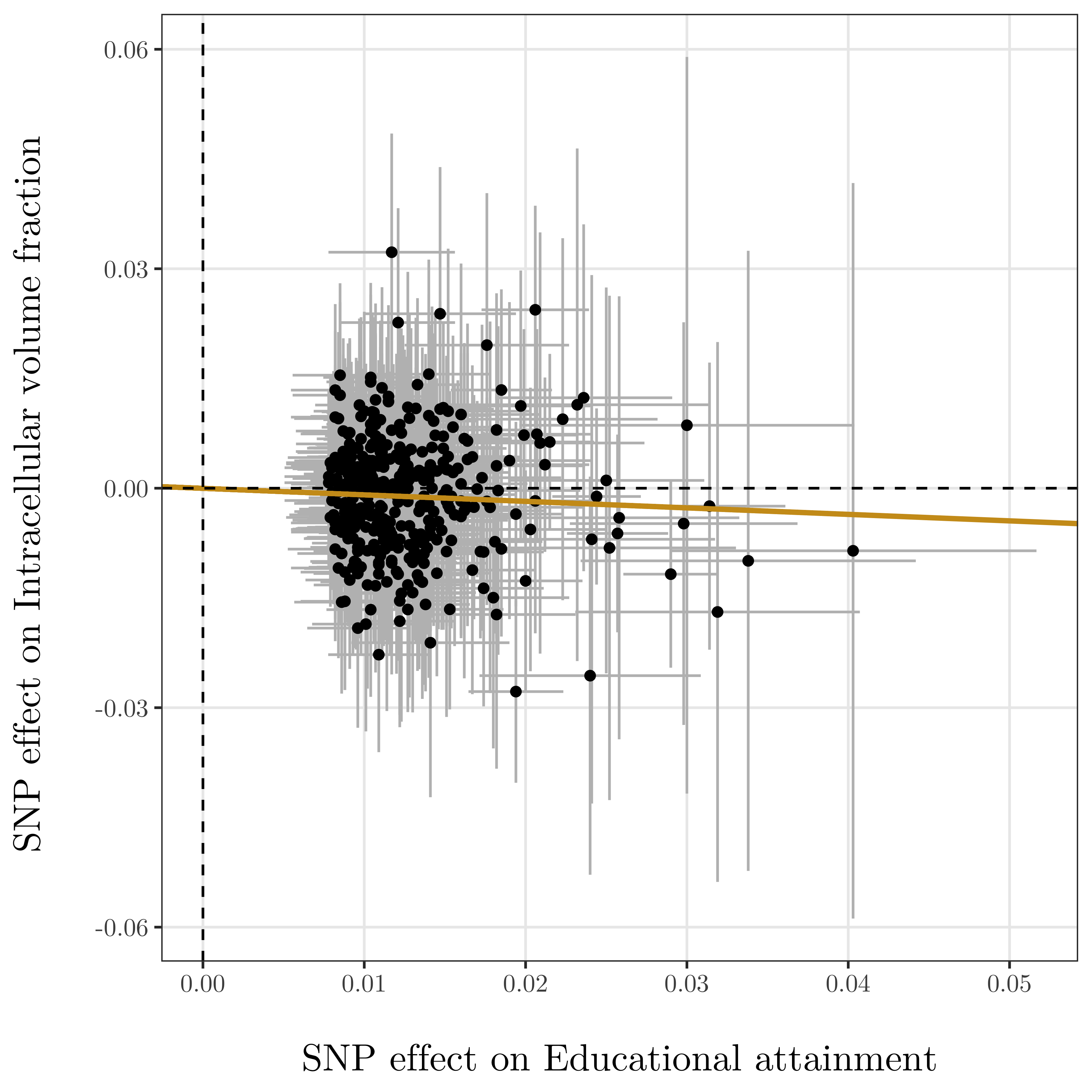 | 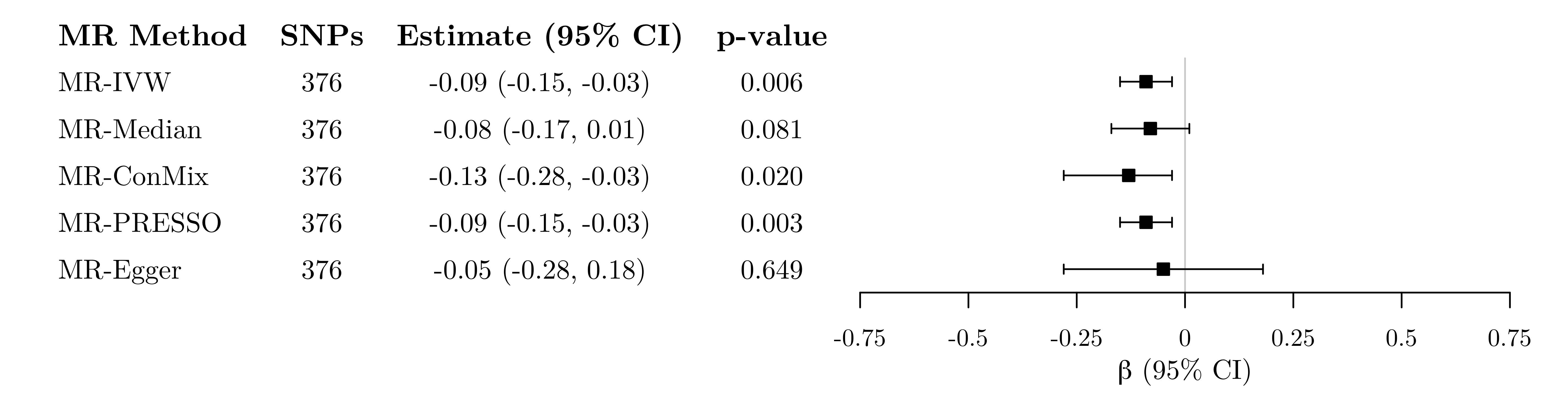 |
| --- | --- |
| 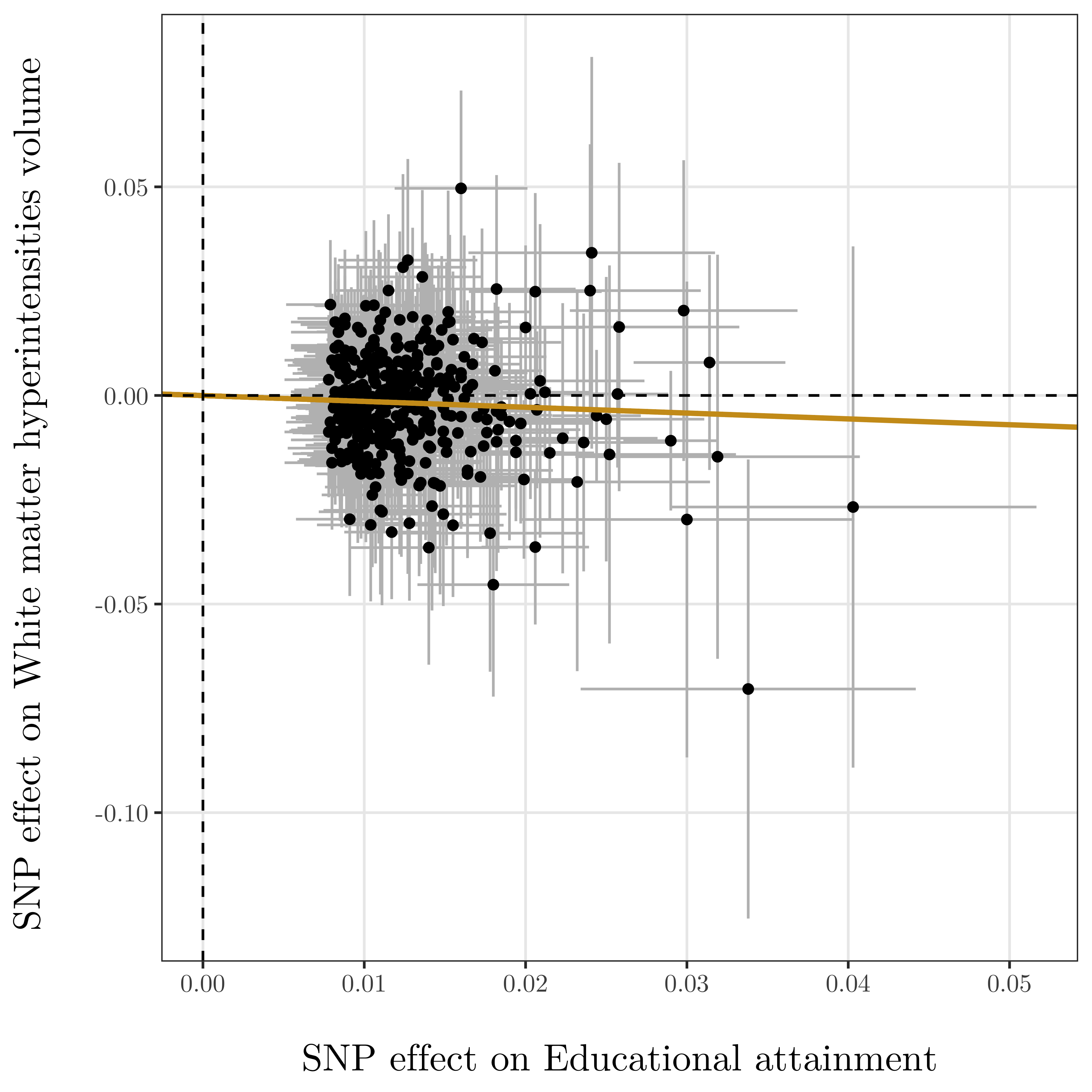 | 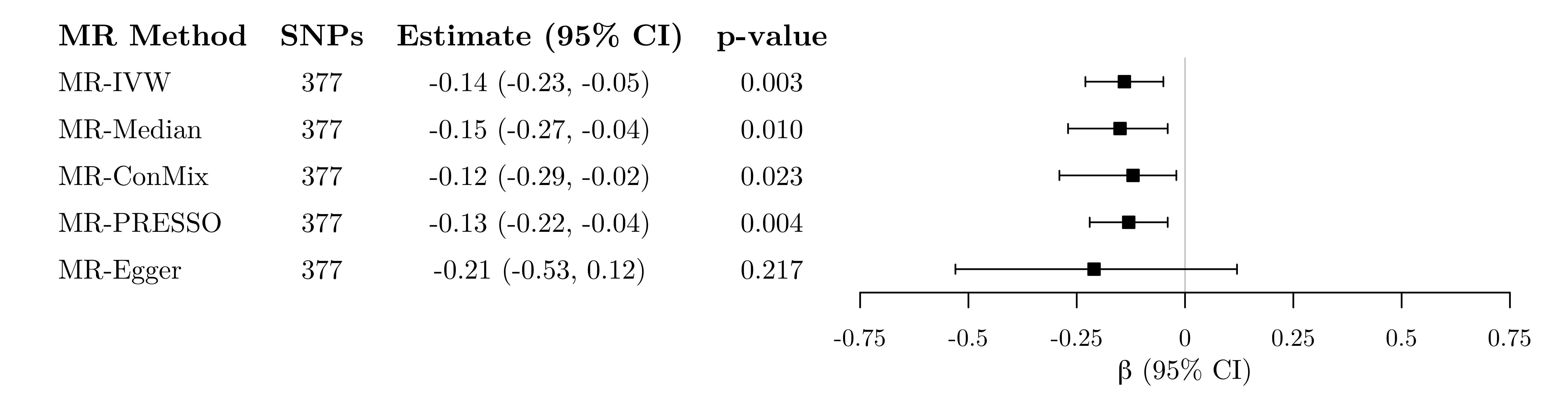 |

**Supplementary Fig. 6 (continued).** Left: Genetic associations with educational attainment (horizontal axis, standard deviation units) and with imaging-derived phenotypes (vertical axis, standard deviation units) for 376 genetic variants associated with educational attainment at a genome-wide level of significance. Horizontal and vertical lines represent 95% confidence intervals for the genetic associations. The regression line through the origin represents the inverse-variance weighted Mendelian randomization estimate for the effect of educational attainment on each imaging-derived phenotype. Right: Mendelian randomization estimates of the association between genetically-proxied educational attainment and imaging-derived brain structure phenotypes. Estimates represent standard deviation change in the imaging phenotype per 1 standard deviation increase in genetically-predicted years of schooling (approximately 4.2 years).

**
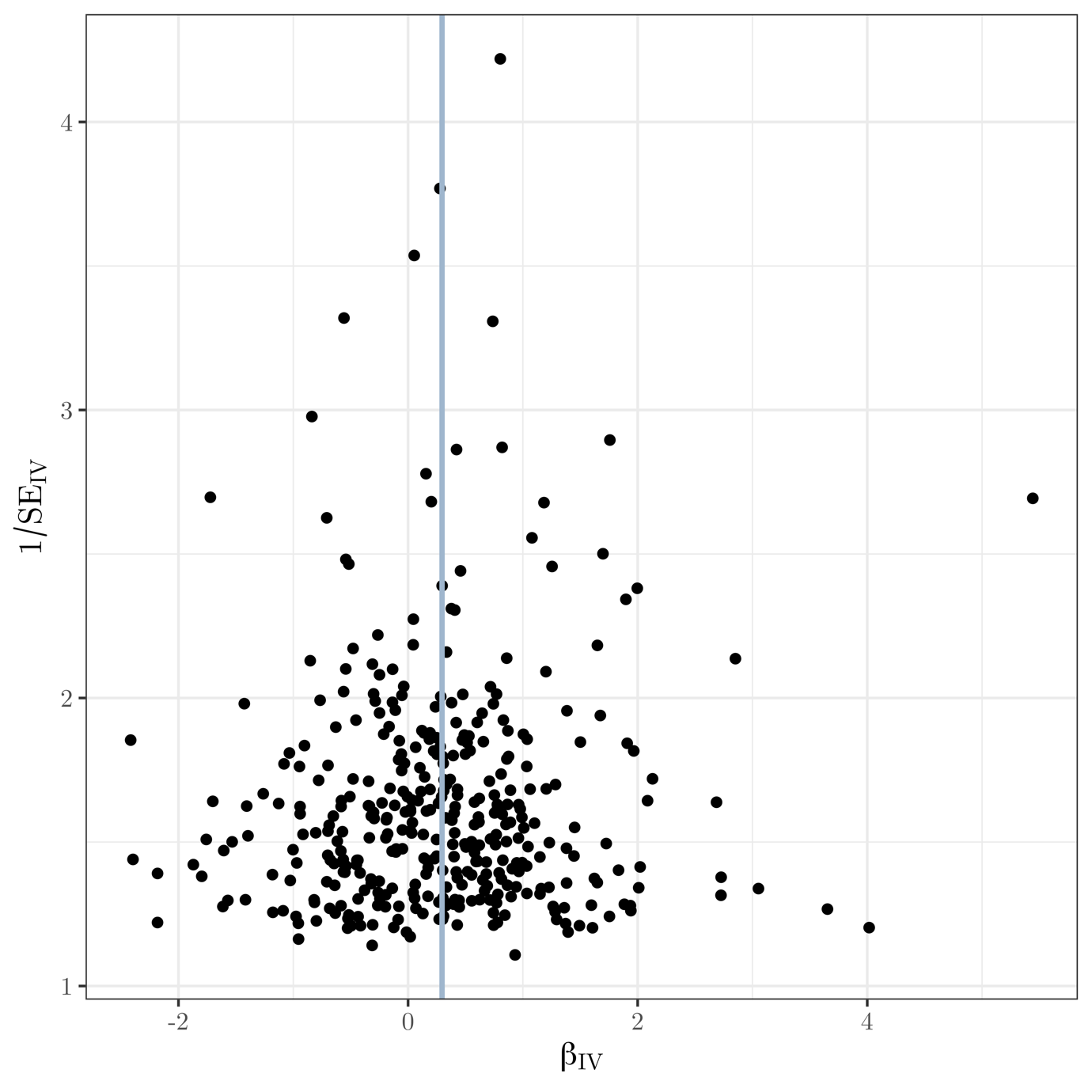
**

**Supplementary Fig. 7** Funnel plot of instrument precision against instrumental variable estimates for each genetic variant separately for Mendelian randomization analysis of educational attainment on surface area. Solid vertical line is the (fixed-effect) inverse-variance weighted estimate.

**
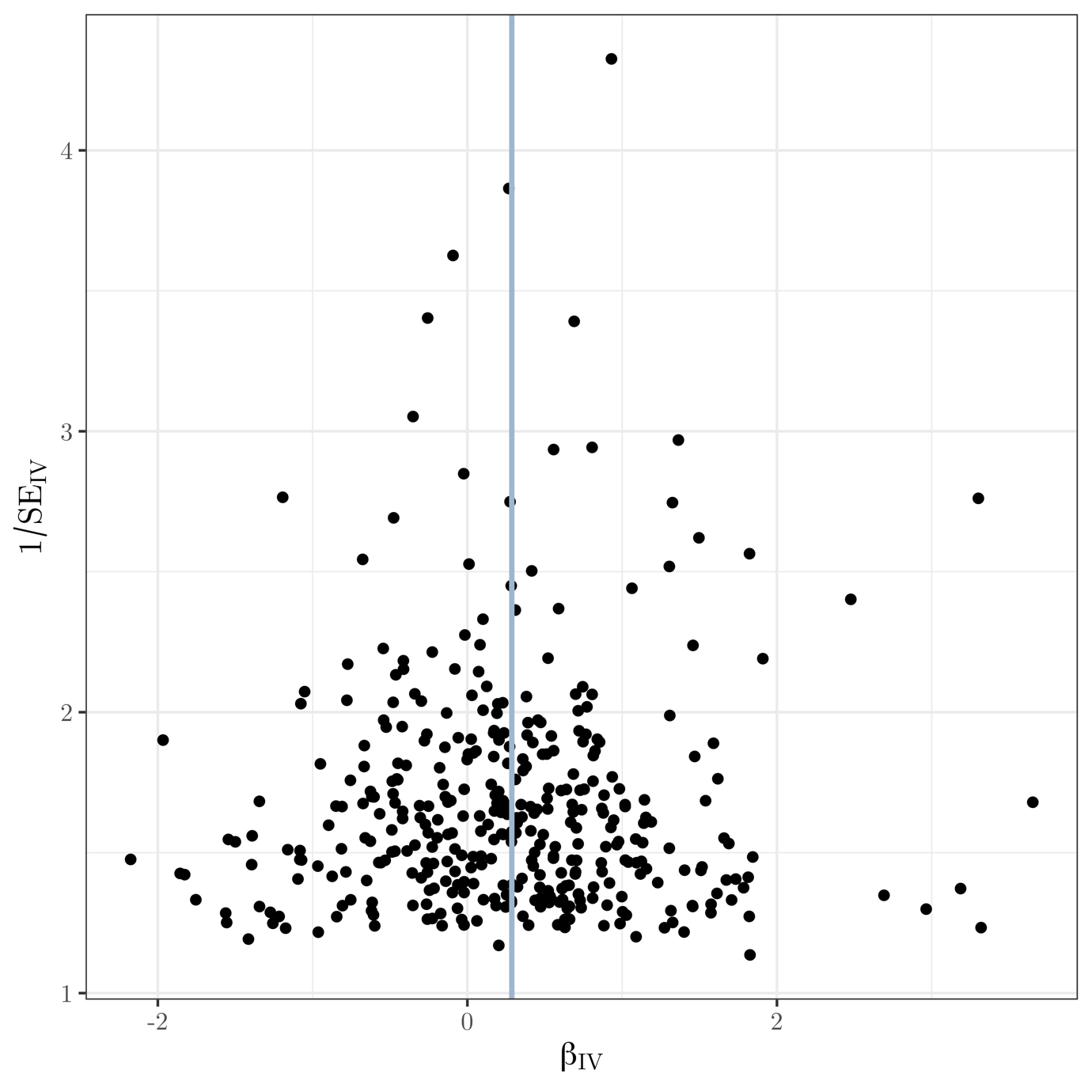
**

**Supplementary Fig. 8** Funnel plot of instrument precision against instrumental variable estimates for each genetic variant separately for Mendelian randomization analysis of educational attainment on volume. Solid vertical line is the (fixed-effect) inverse-variance weighted estimate.

**
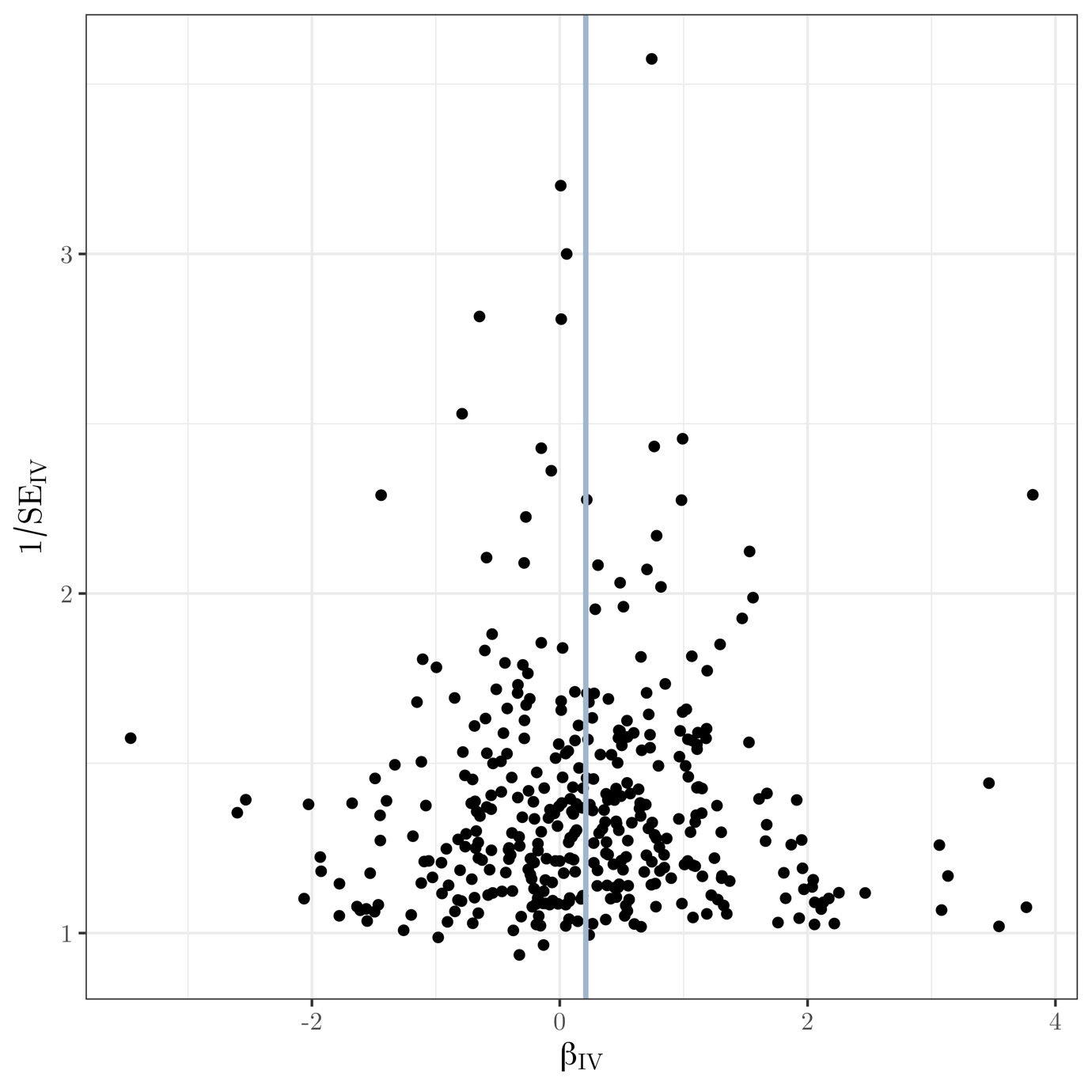
**

**Supplementary Fig. 9** Funnel plot of instrument precision against instrumental variable estimates for each genetic variant separately for Mendelian randomization analysis of educational attainment on local gyrification index. Solid vertical line is the (fixed-effect) inverse-variance weighted estimate.

**
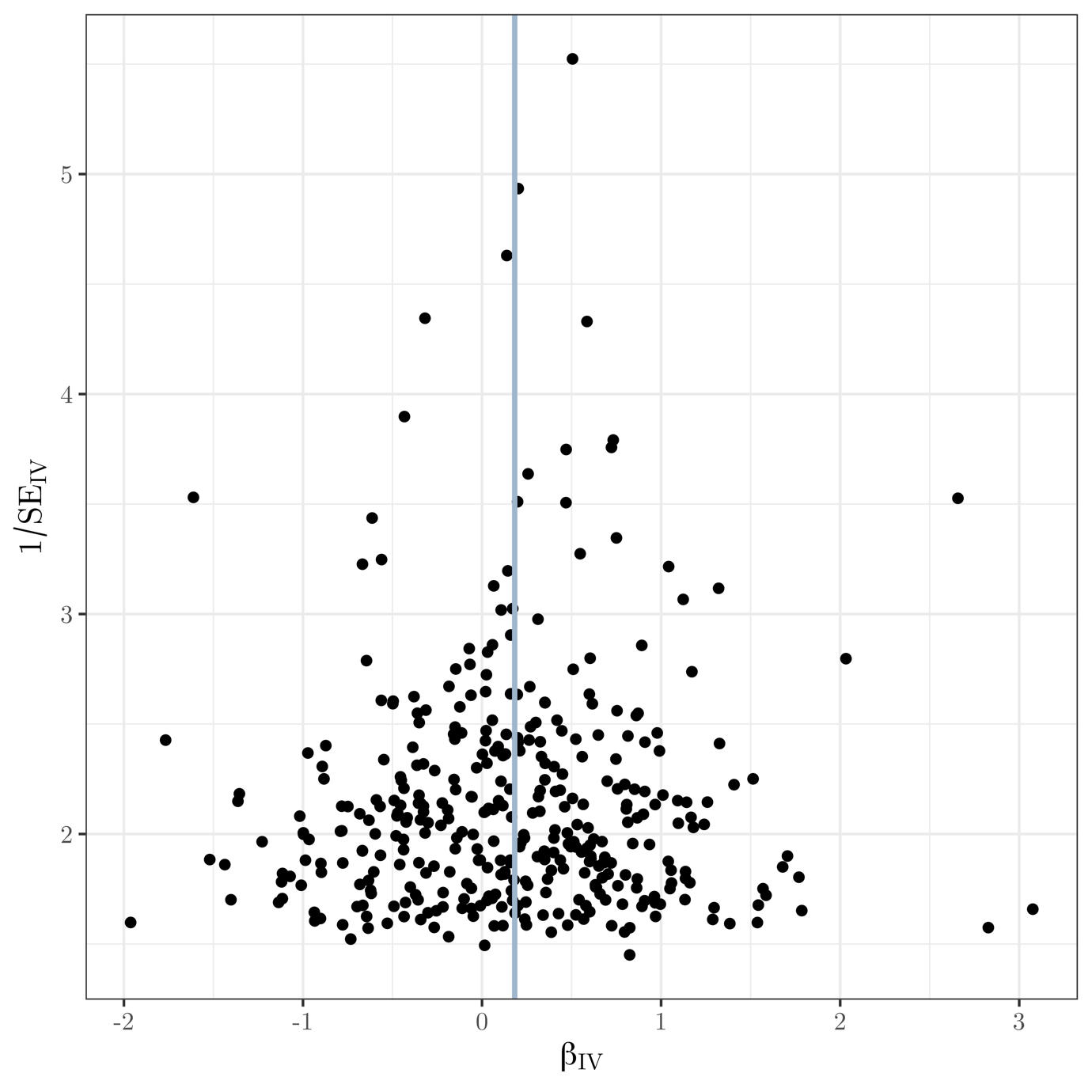
**

**Supplementary Fig. 10** Funnel plot of instrument precision against instrumental variable estimates for each genetic variant separately for Mendelian randomization analysis of educational attainment on intrinsic curvature. Solid vertical line is the (fixed-effect) inverse-variance weighted estimate.

**
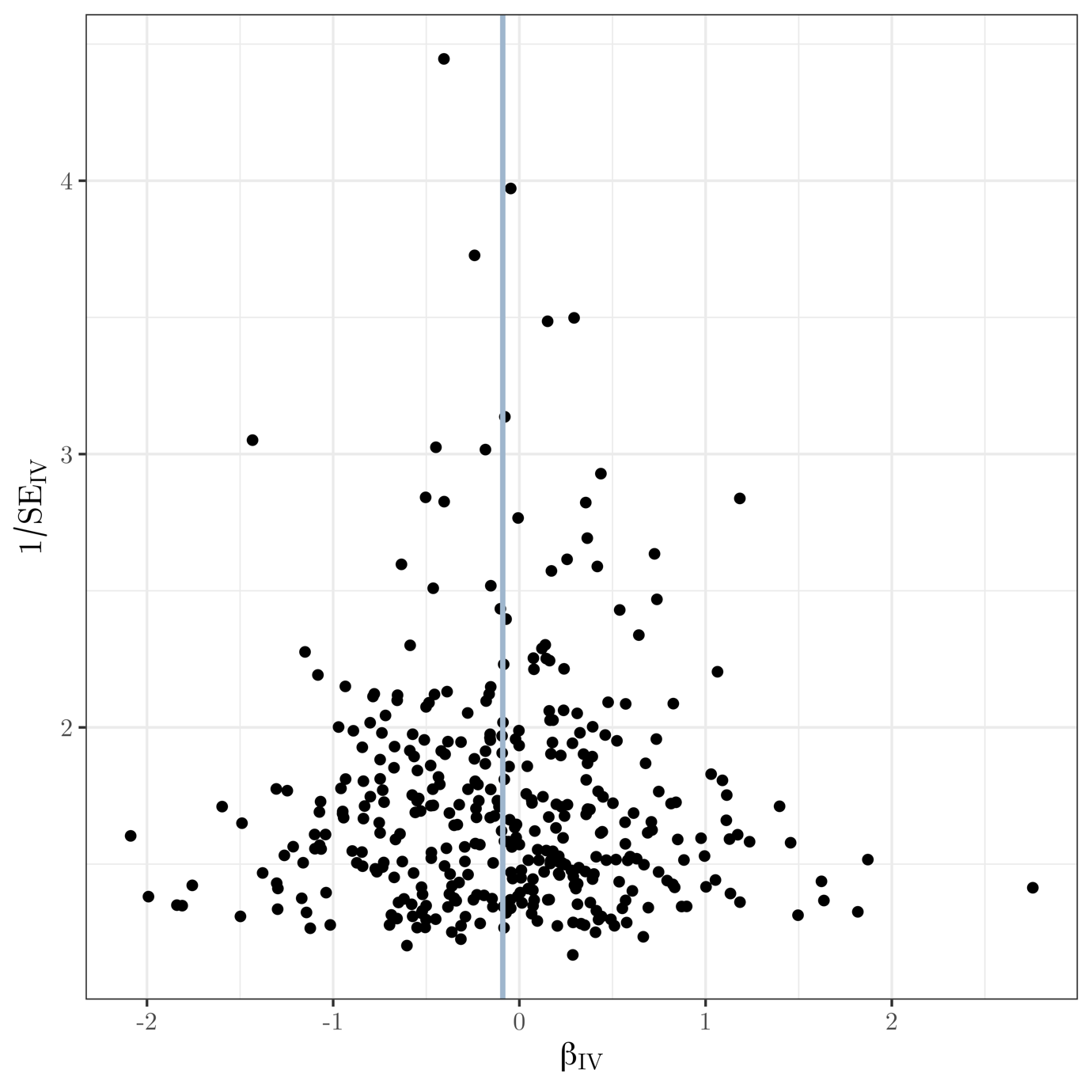
**

**Supplementary Fig. 11** Funnel plot of instrument precision against instrumental variable estimates for each genetic variant separately for Mendelian randomization analysis of educational attainment on intracellular volume fraction. Solid vertical line is the (fixed-effect) inverse-variance weighted estimate.

**
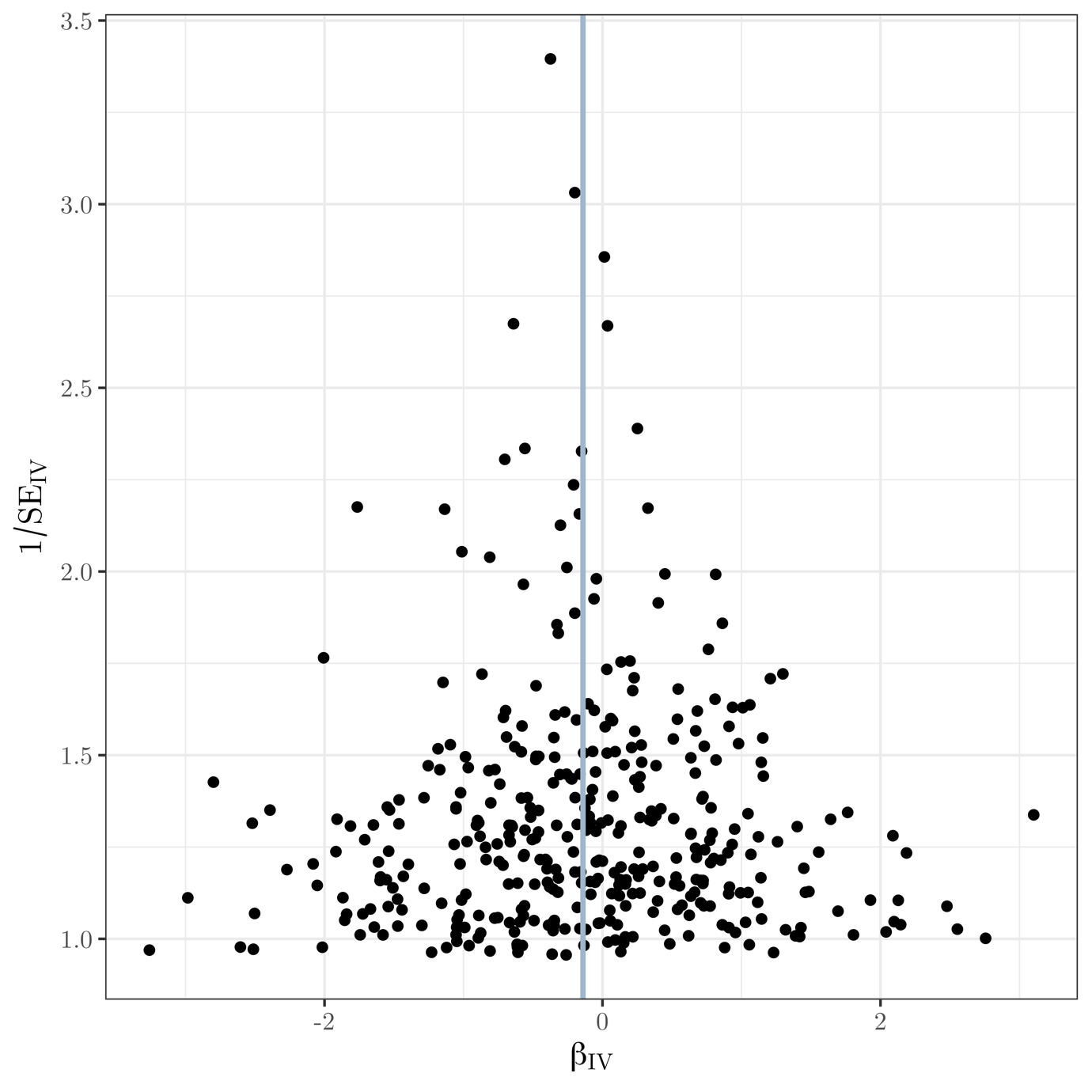
**

**Supplementary Fig. 12** Funnel plot of instrument precision against instrumental variable estimates for each genetic variant separately for Mendelian randomization analysis of educational attainment on total volume of white matter hyperintensities. Solid vertical line is the (fixed-effect) inverse-variance weighted estimate.

**
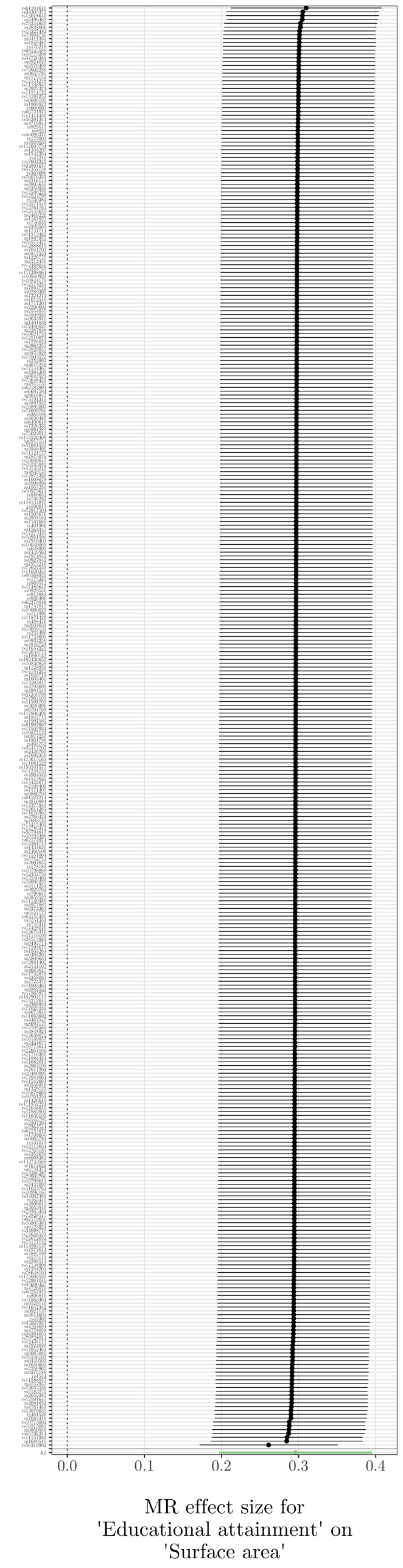
**

**Supplementary Fig. 13** Leave-one-out plot for MR analysis of educational attainment on surface area.

**
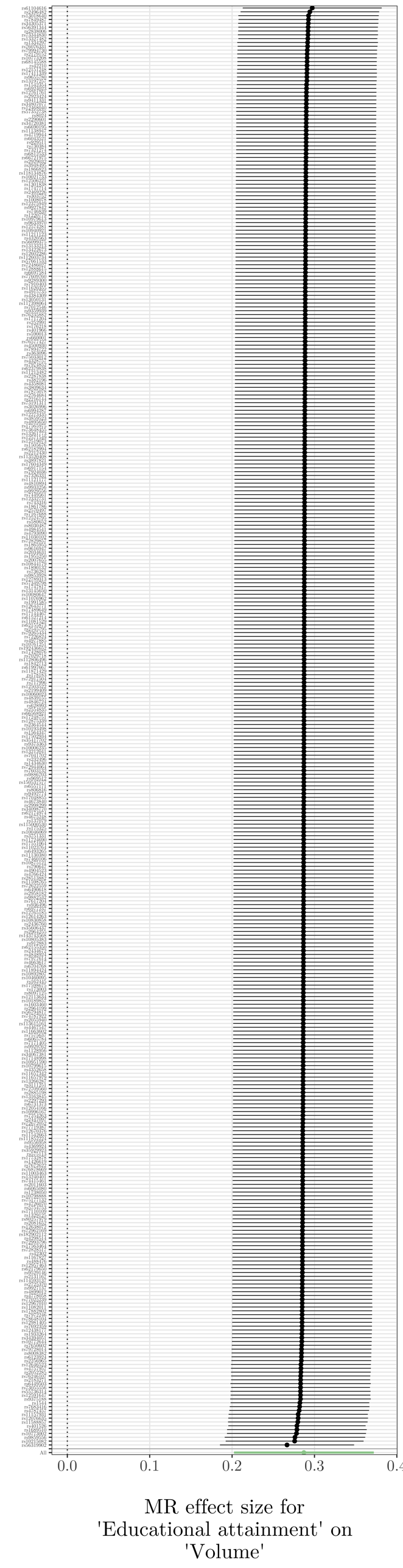
**

**Supplementary Fig. 14** Leave-one-out plot for MR analysis of educational attainment on volume.

**
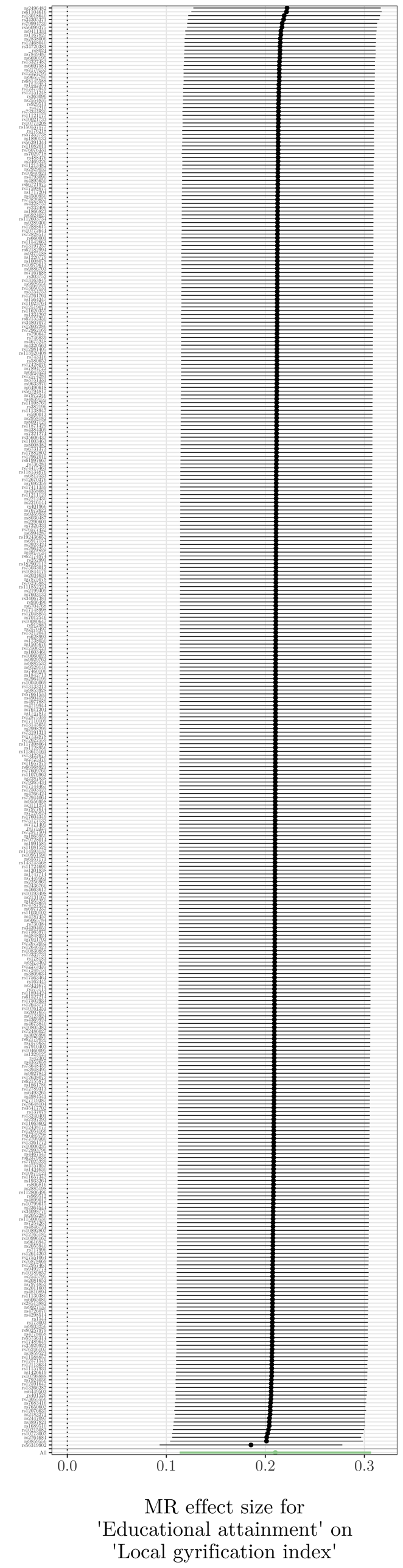
**

**Supplementary Fig. 15** Leave-one-out plot for MR analysis of educational attainment on local gyrification index.

**
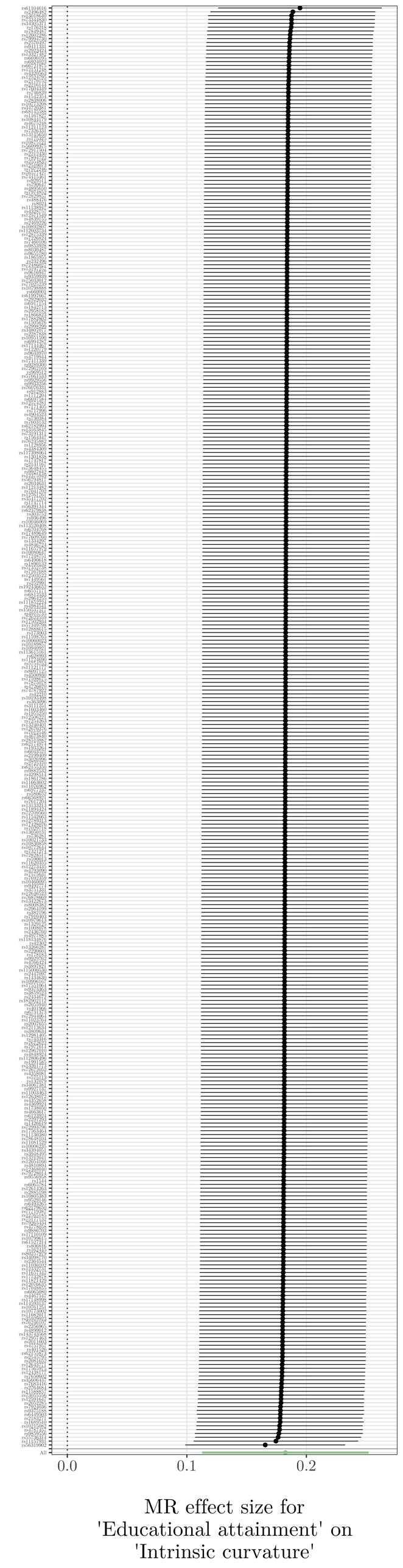
**

**Supplementary Fig. 16** Leave-one-out plot for MR analysis of educational attainment on intrinsic curvature.

**
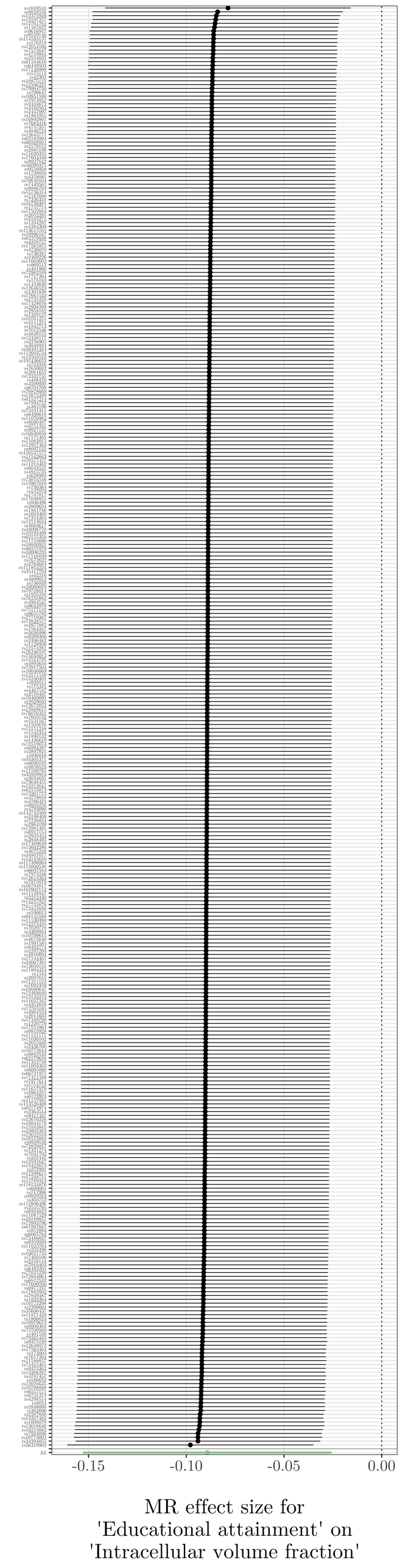
**

**Supplementary Fig. 17** Leave-one-out plot for MR analysis of educational attainment on intracellular volume fraction.

**
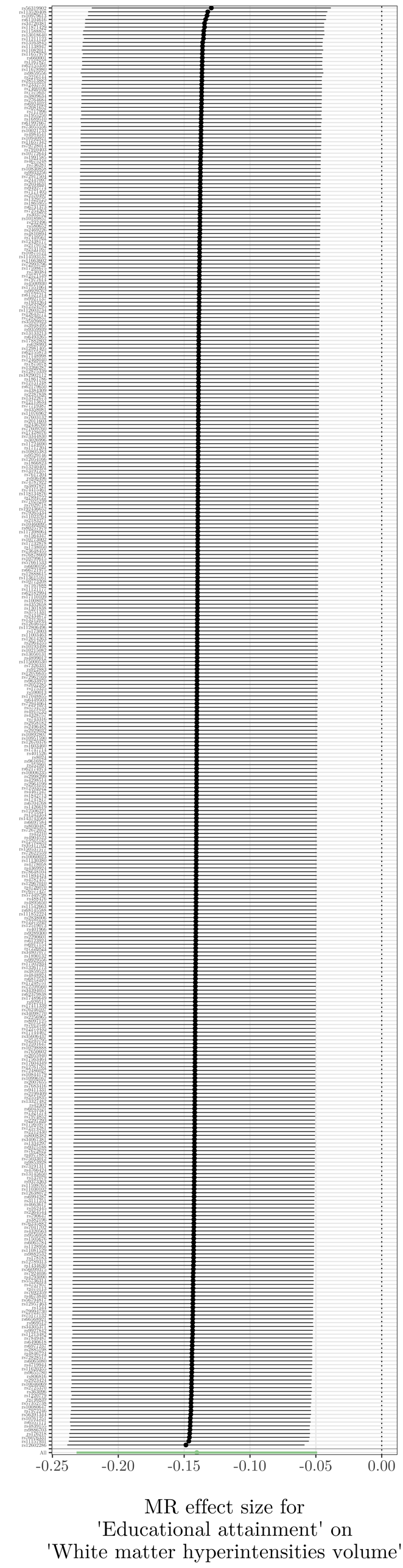
**

**Supplementary Fig. 18** Leave-one-out plot for MR analysis of educational attainment on total volume of white matter hyperintensities.

| 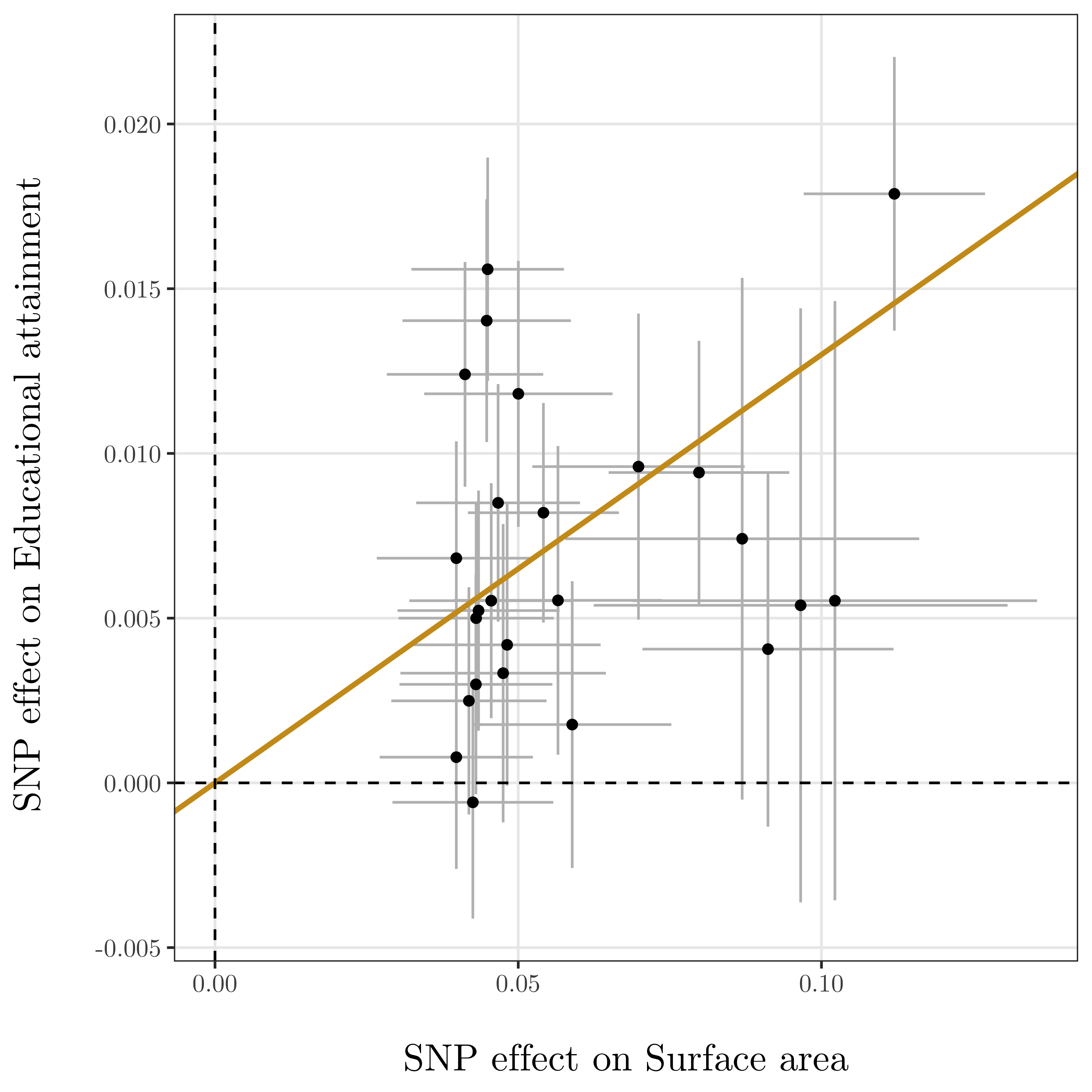 | 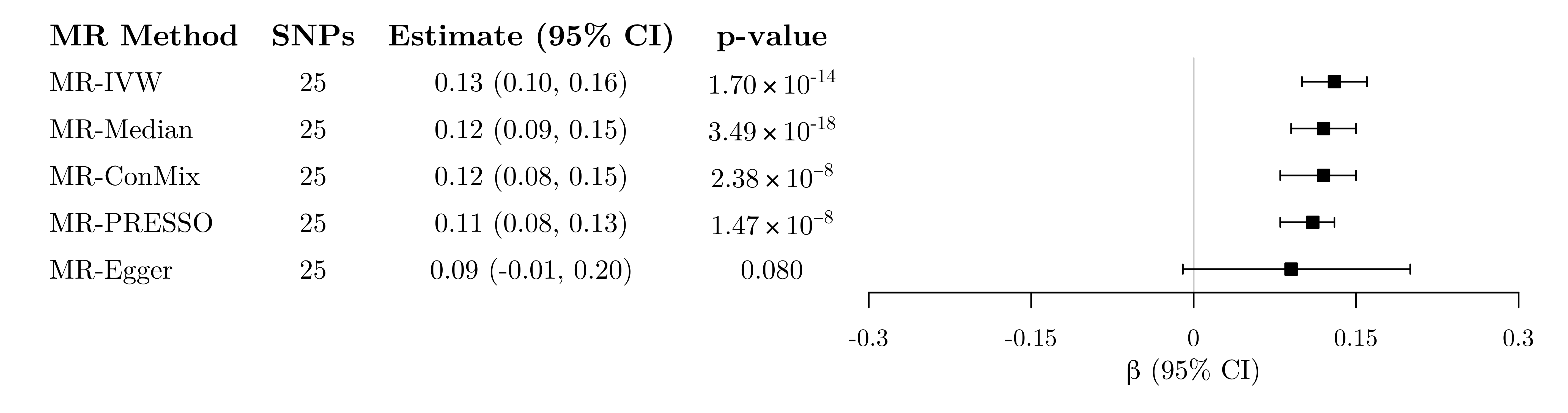 |
| --- | --- |
| 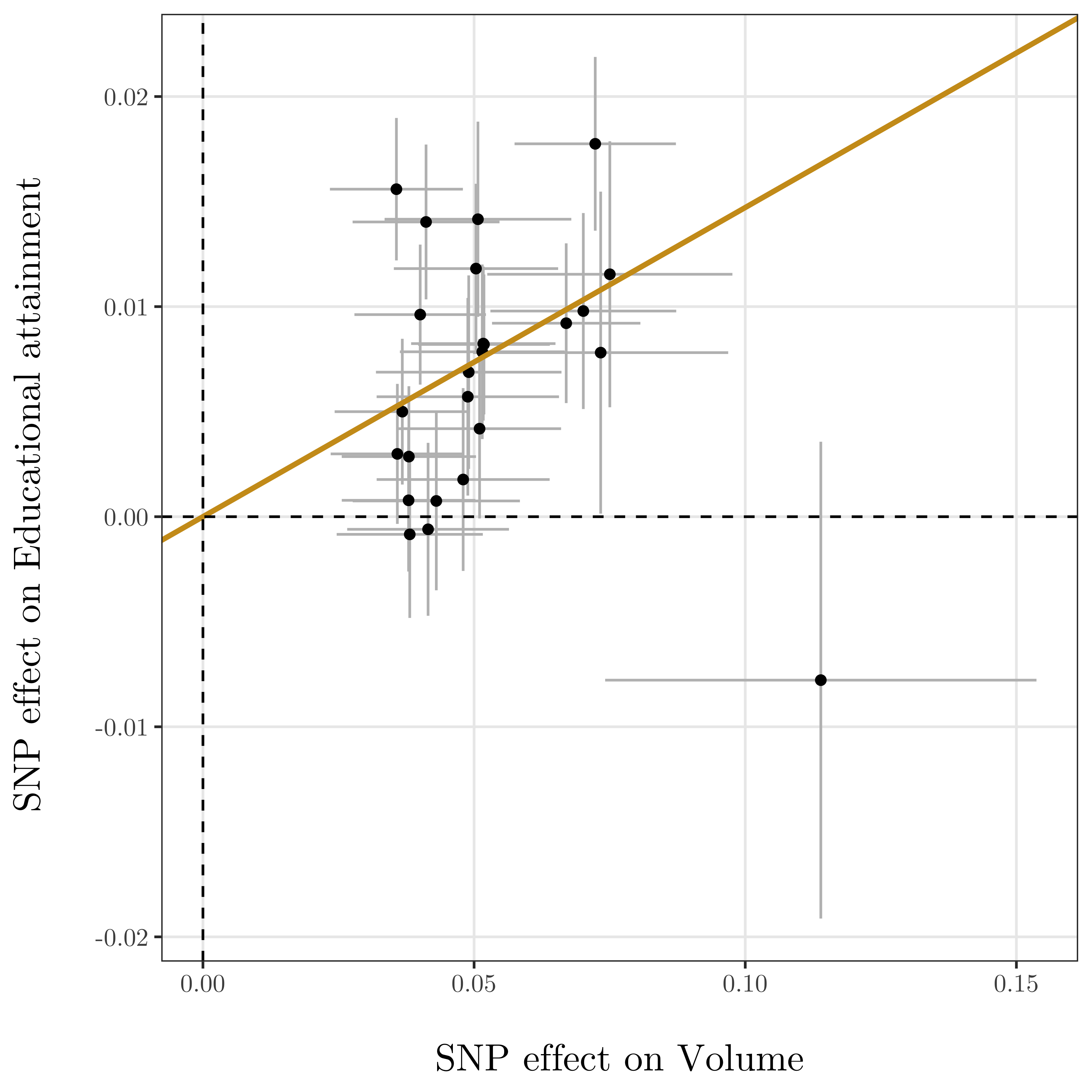 | 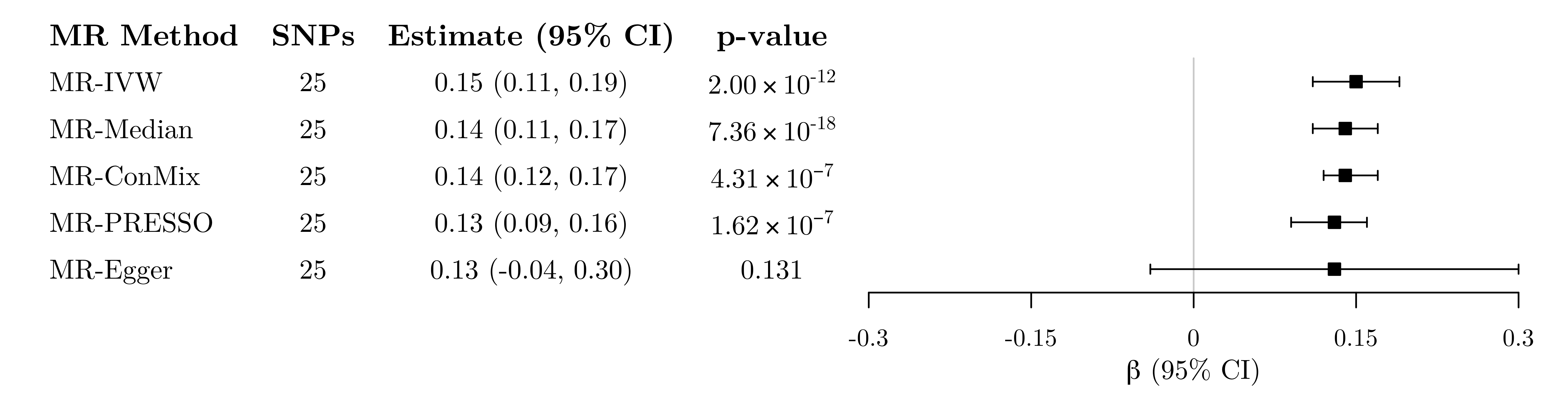 |
| 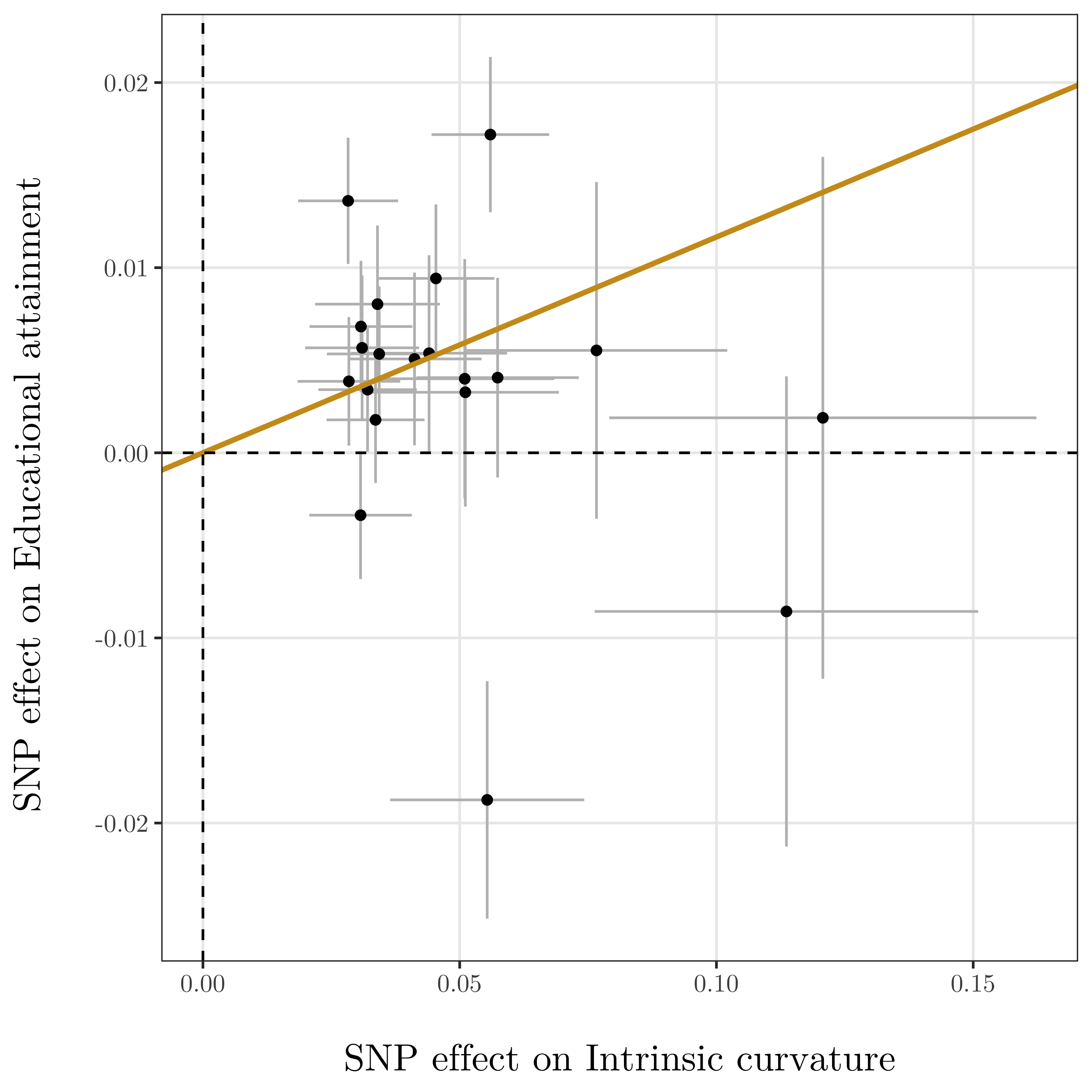 | 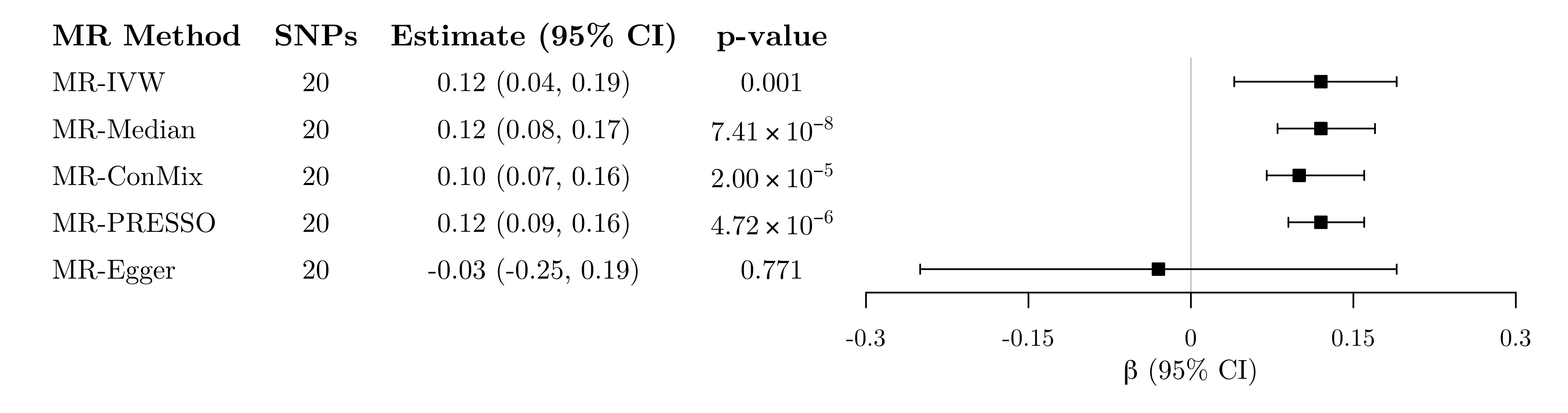 |

**Supplementary Fig. 19** Left: Genetic associations with imaging-derived phenotypes (horizontal axis, standard deviation units) and educational attainment (vertical axis, standard deviation units) for genetic variants associated with each imaging-derived phenotype at a genome-wide level of significance. Horizontal and vertical lines represent 95% confidence intervals for the genetic associations. The regression line through the origin represents the inverse-variance weighted Mendelian randomization estimate for the effect of each imaging-derived phenotype on educational attainment. Right: Mendelian randomization estimates of the association between genetically-proxied brain structure phenotypes and educational attainment. Estimates represent the standard deviation change in years of schooling (95% confidence intervals) per 1 standard deviation increase in genetically-predicted levels of each imaging-derived phenotype.

**
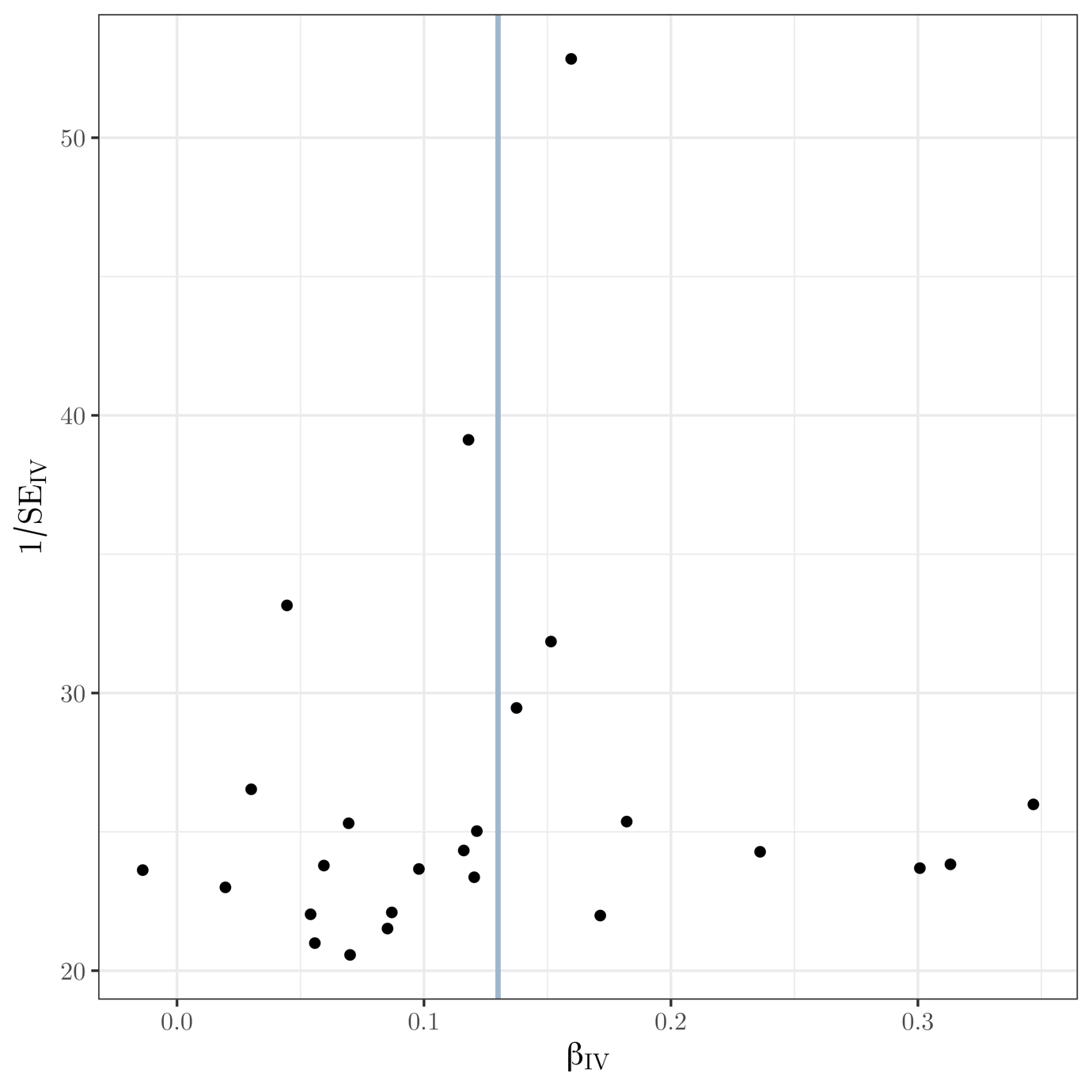
**

**Supplementary Fig. 20** Funnel plot of instrument precision against instrumental variable estimates for each genetic variant separately for Mendelian randomization analysis of surface area on educational attainment. Solid vertical line is the (fixed-effect) inverse-variance weighted estimate.

**
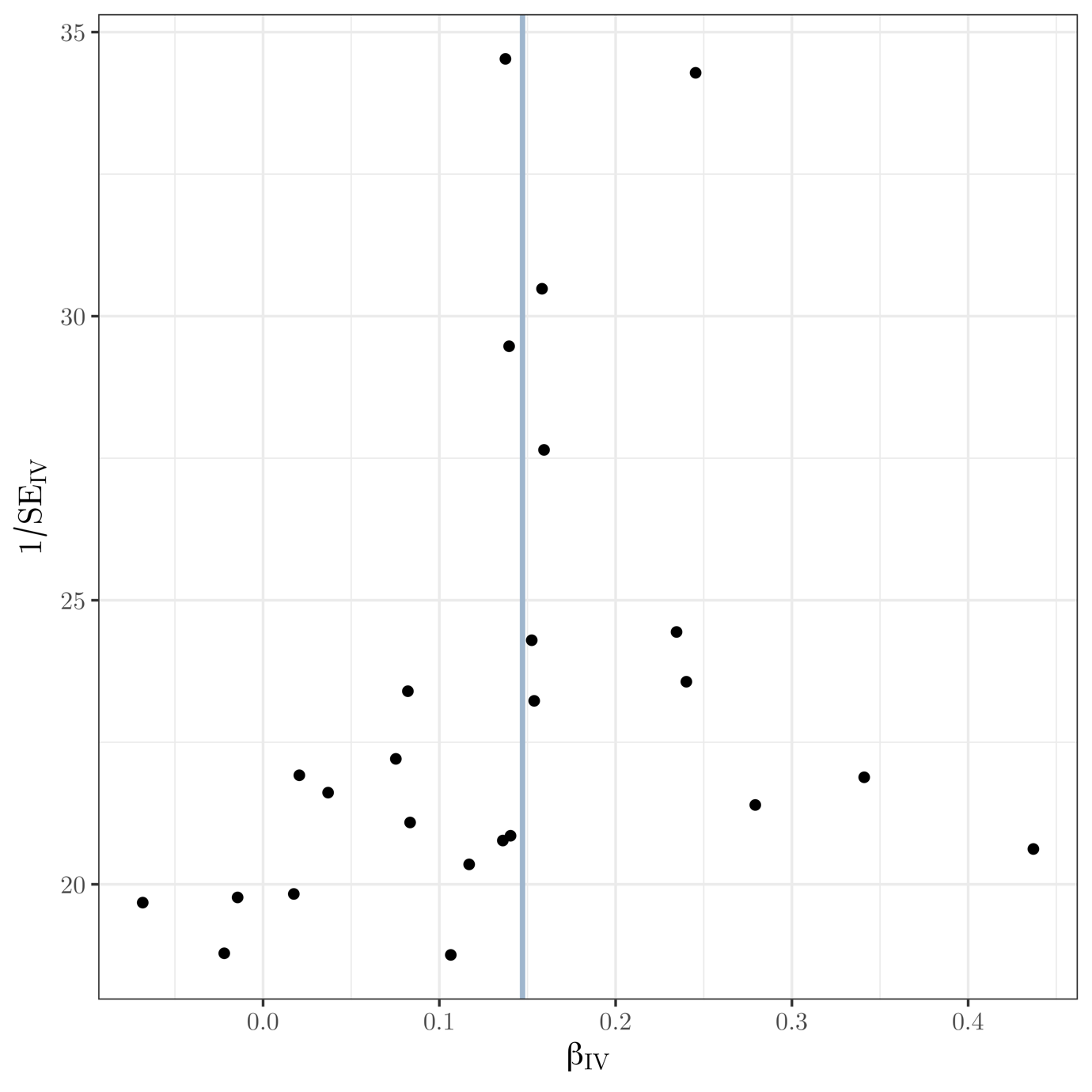
**

**Supplementary Fig. 21** Funnel plot of instrument precision against instrumental variable estimates for each genetic variant separately for Mendelian randomization analysis of volume on educational attainment. Solid vertical line is the (fixed-effect) inverse-variance weighted estimate.

**
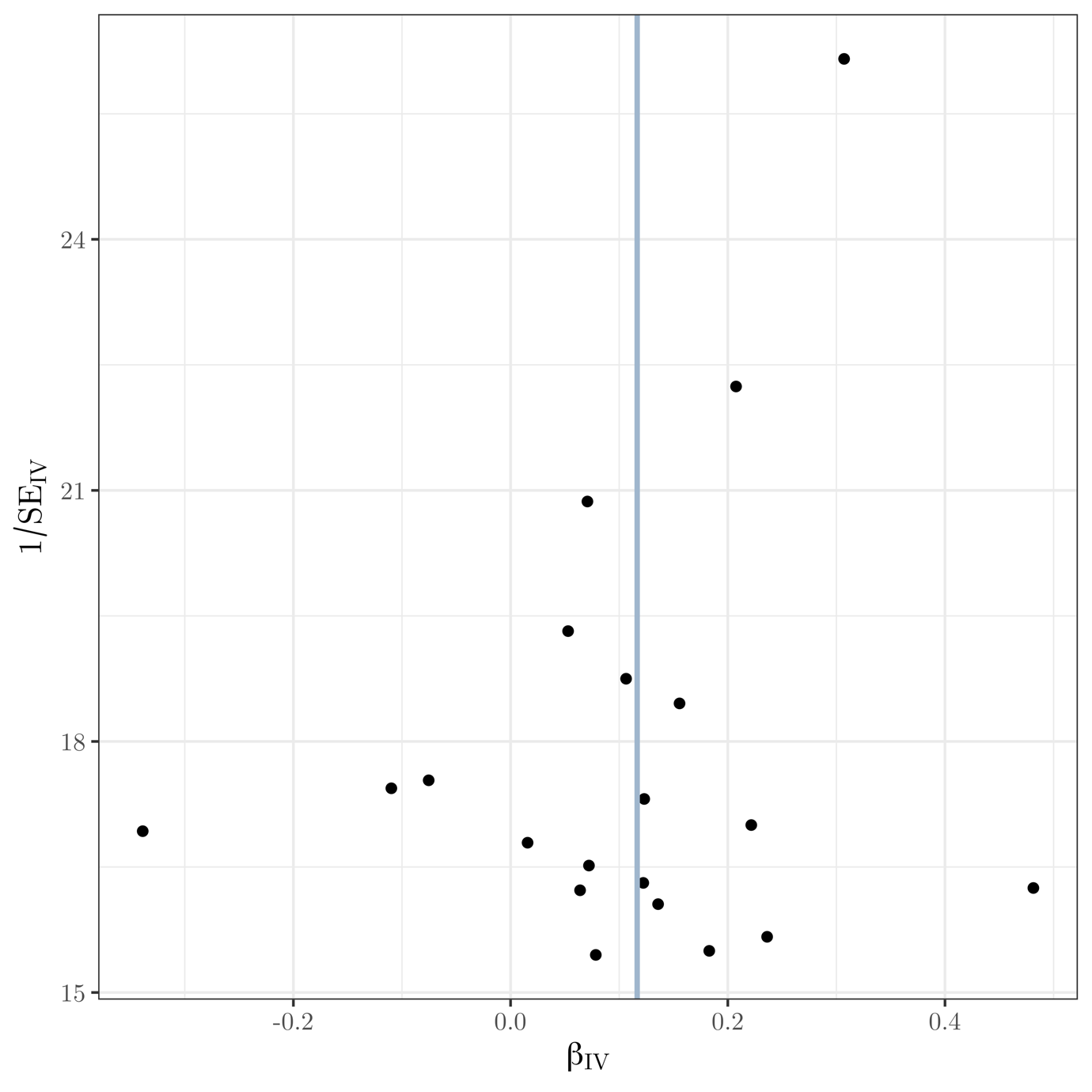
**

**Supplementary Fig. 22** Funnel plot of instrument precision against instrumental variable estimates for each genetic variant separately for Mendelian randomization analysis of intrinsic curvature on educational attainment. Solid vertical line is the (fixed-effect) inverse-variance weighted estimate.

**
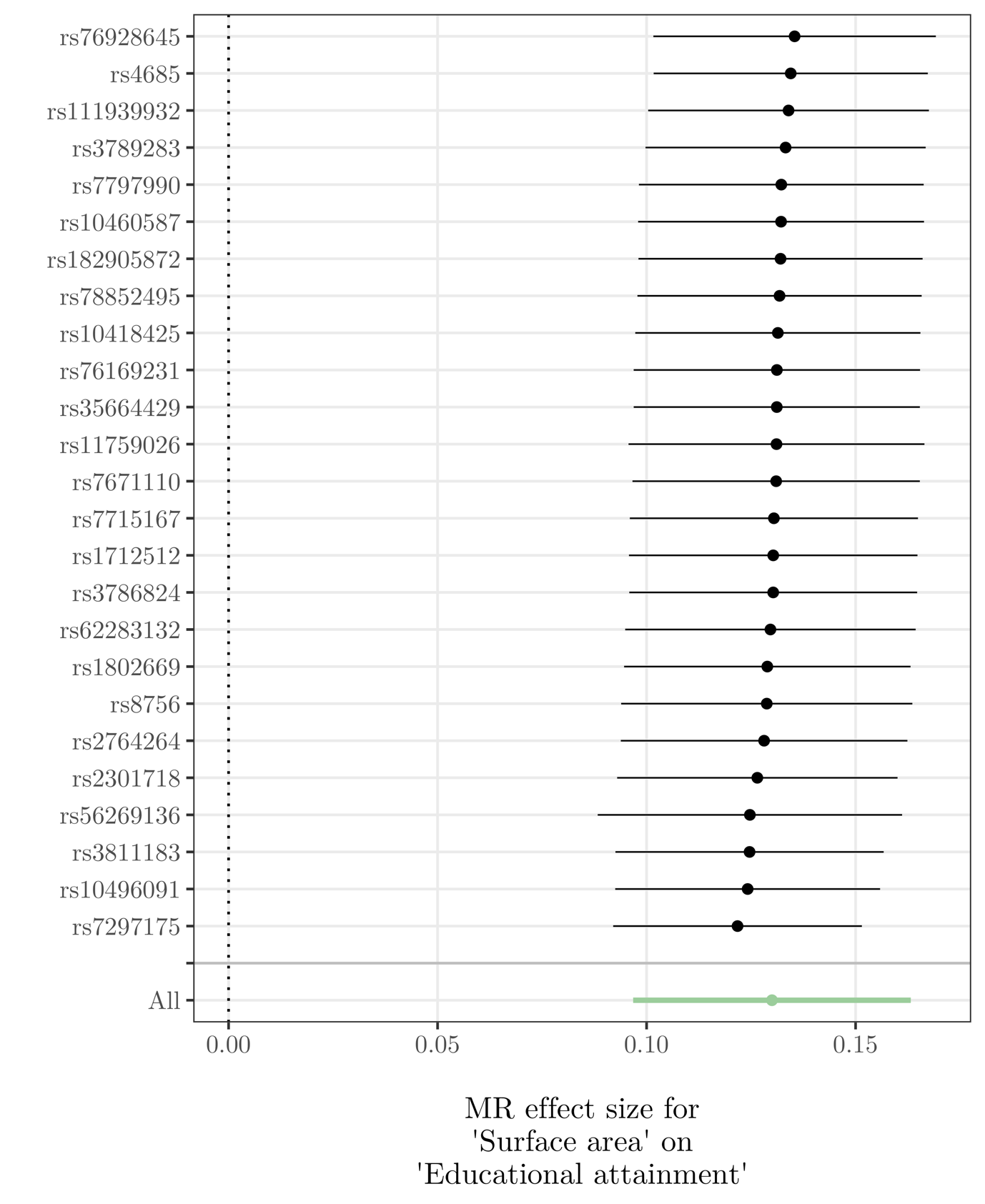
**

**Supplementary Fig. 23** Leave-one-out plot for MR analysis of surface area on educational attainment.

**
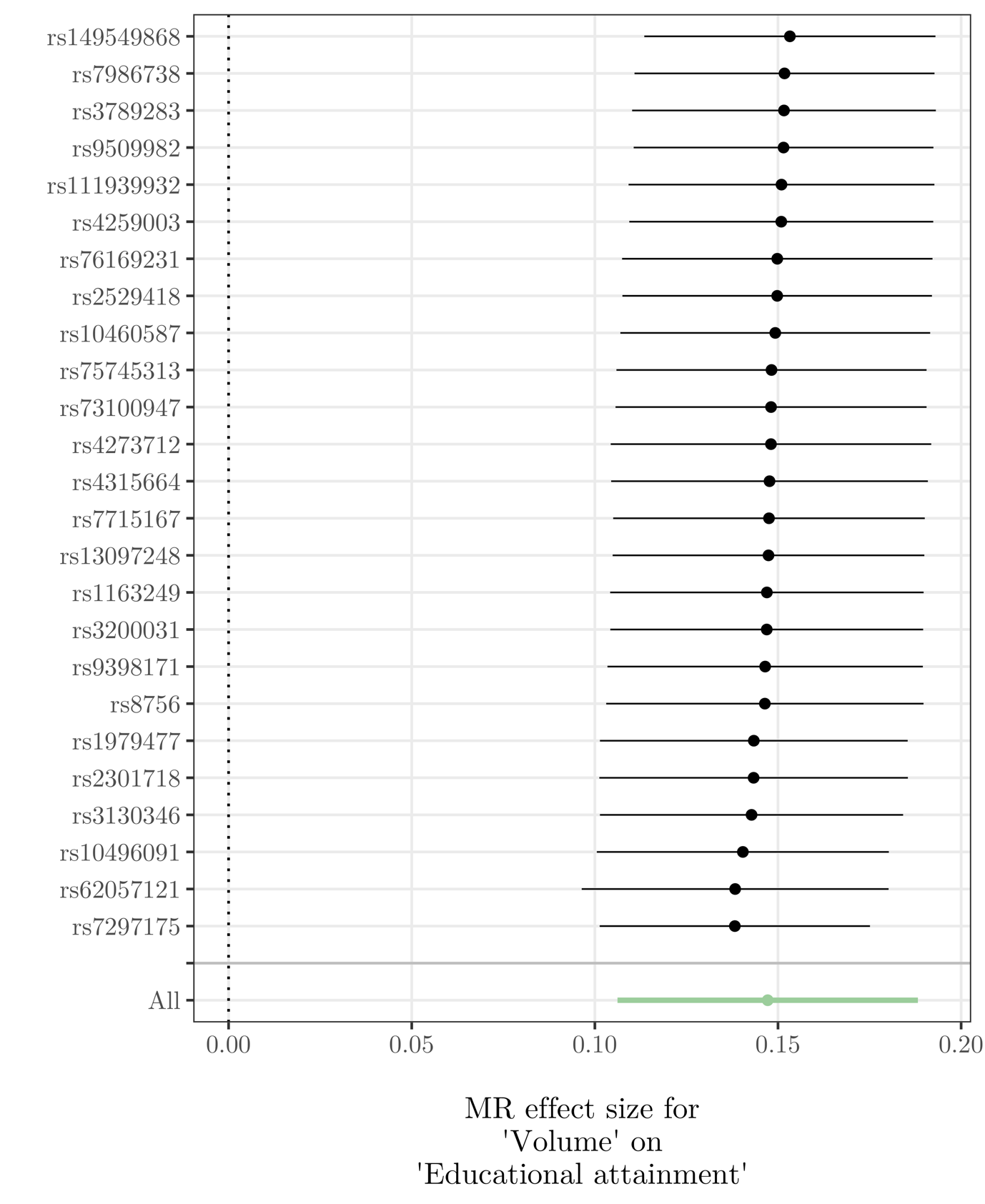
**

**Supplementary Fig. 24** Leave-one-out plot for MR analysis of volume on educational attainment.

**
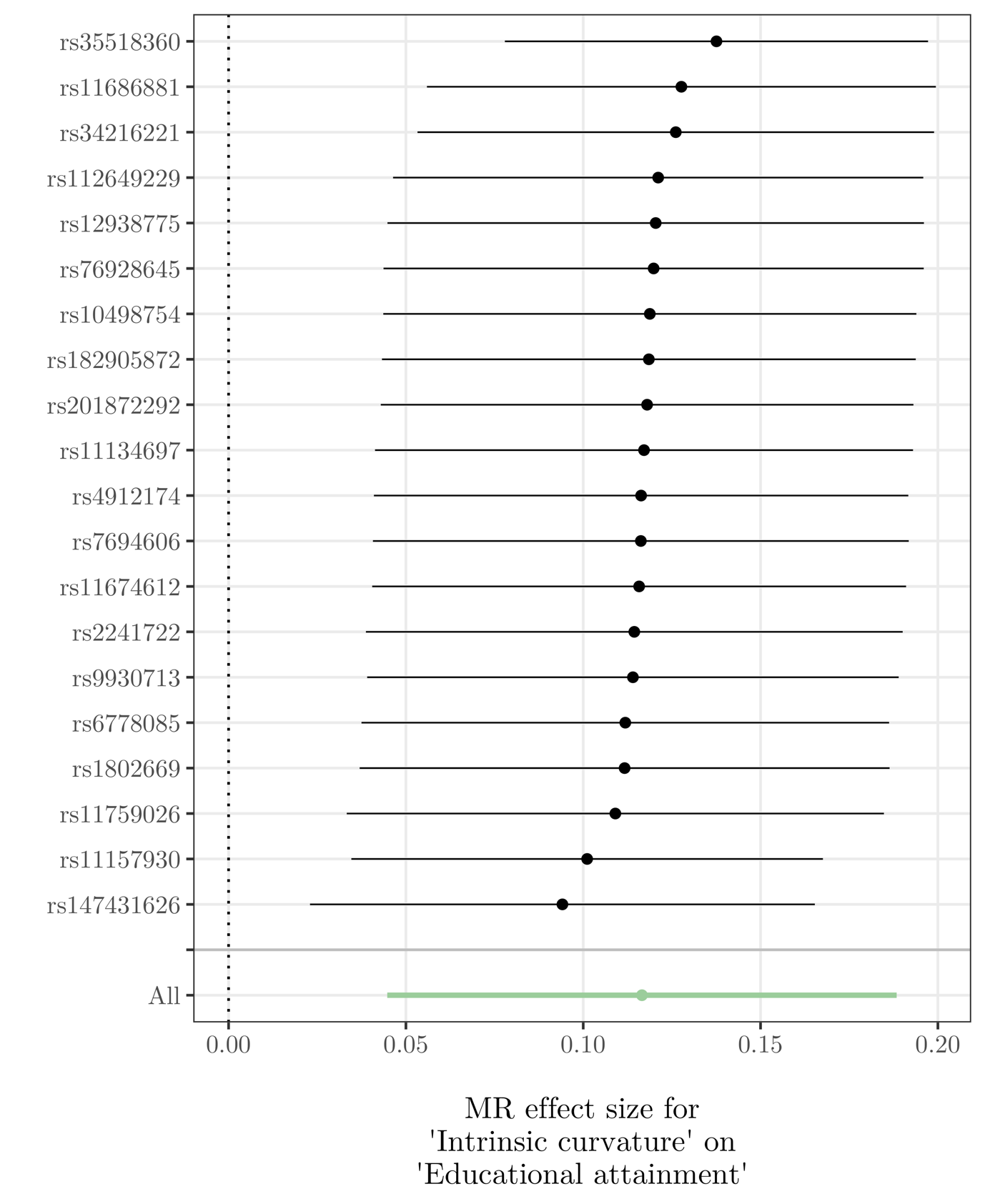
**

**Supplementary Fig. 25** Leave-one-out plot for MR analysis of intrinsic curvature on educational attainment.

| 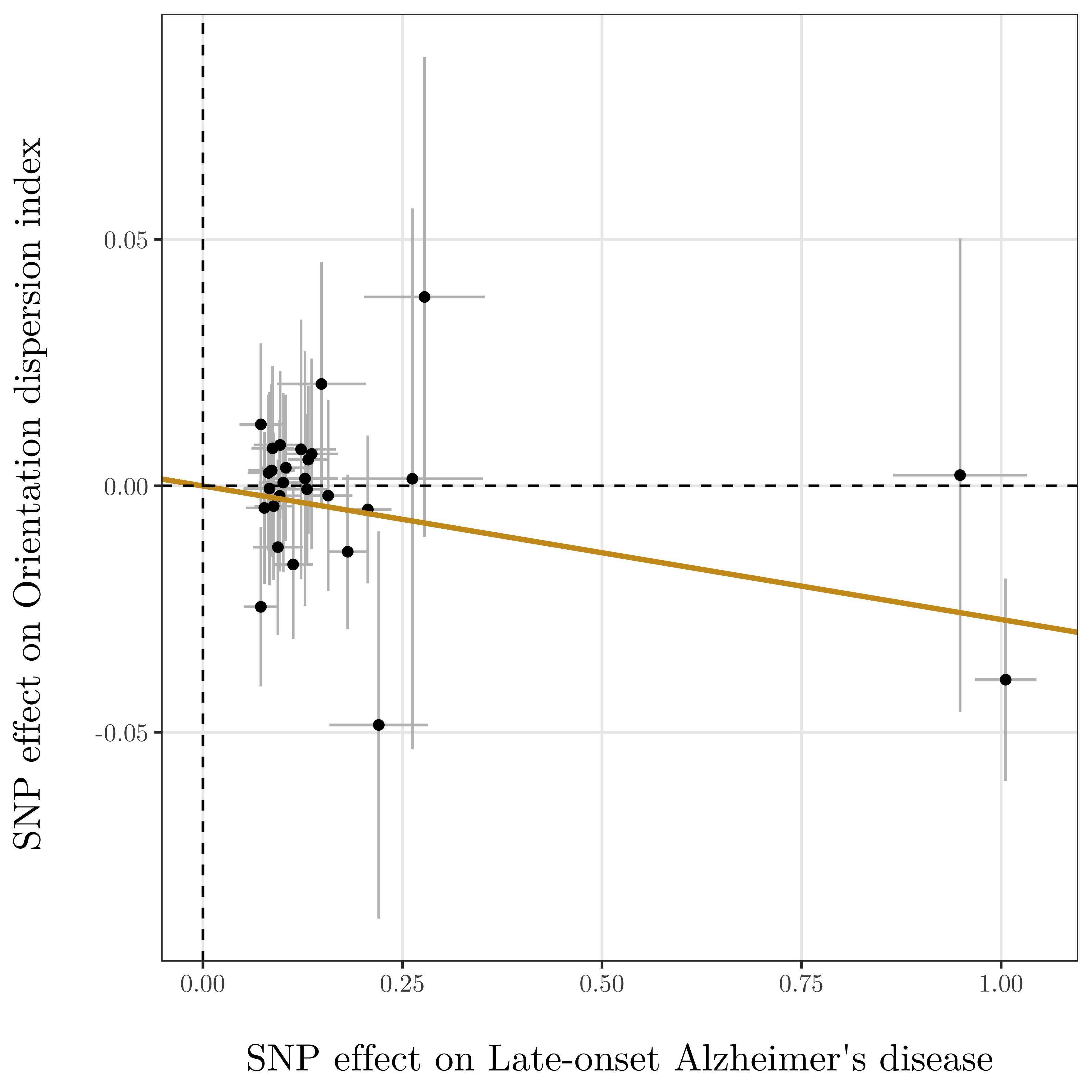 | 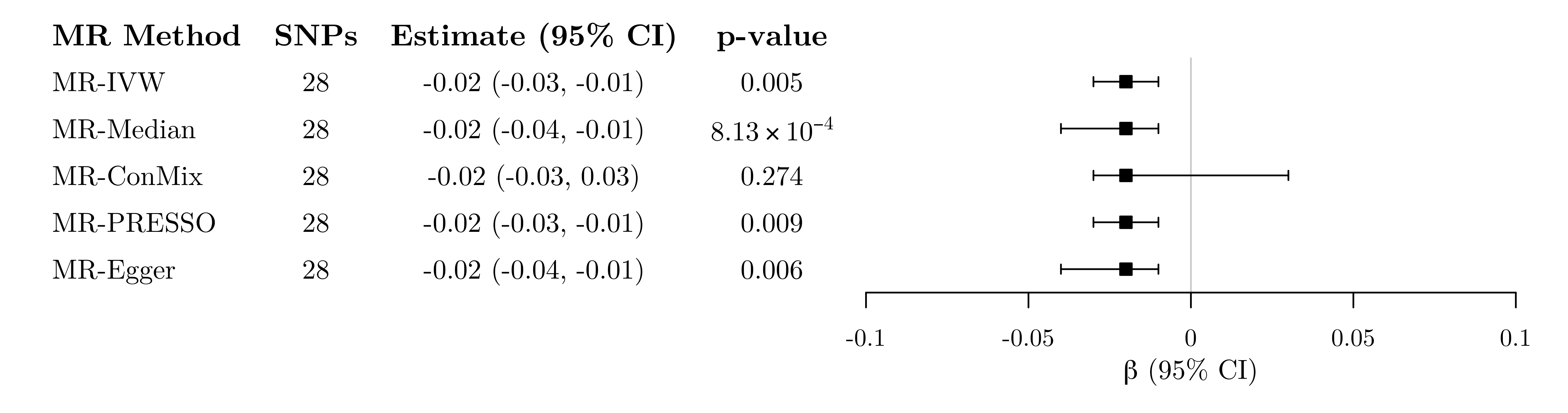 |
| --- | --- |
| 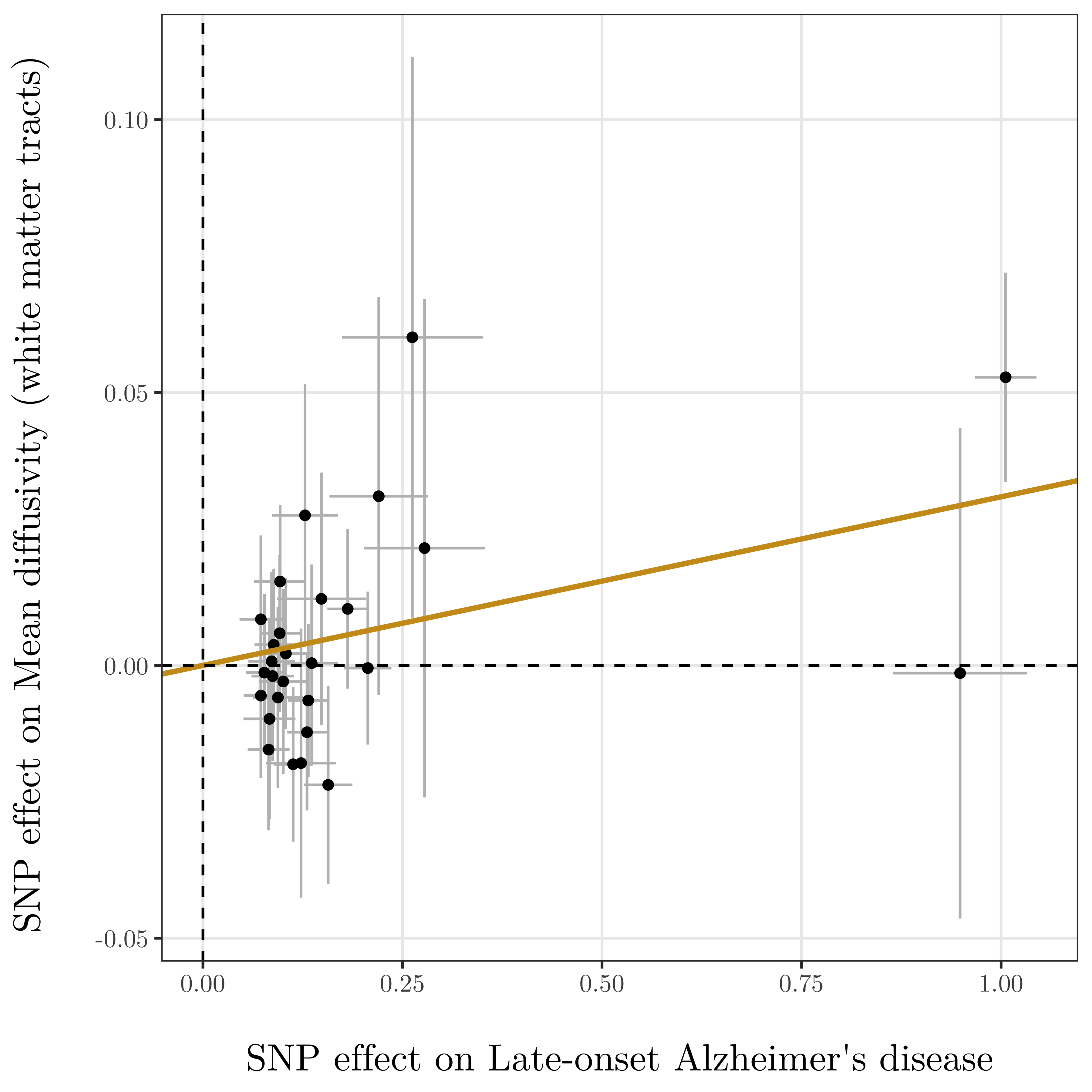 | 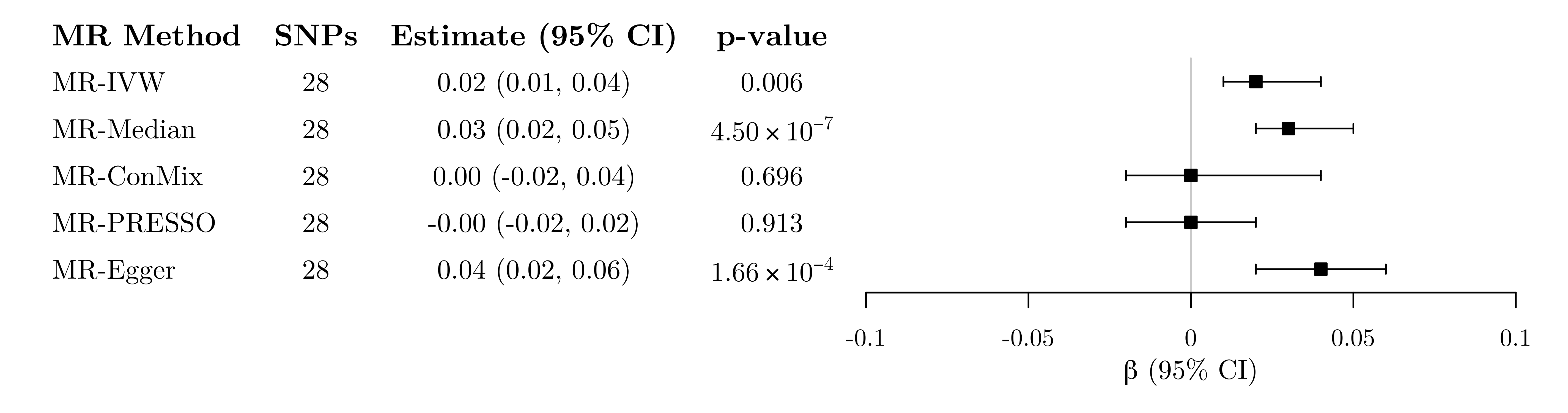 |
| 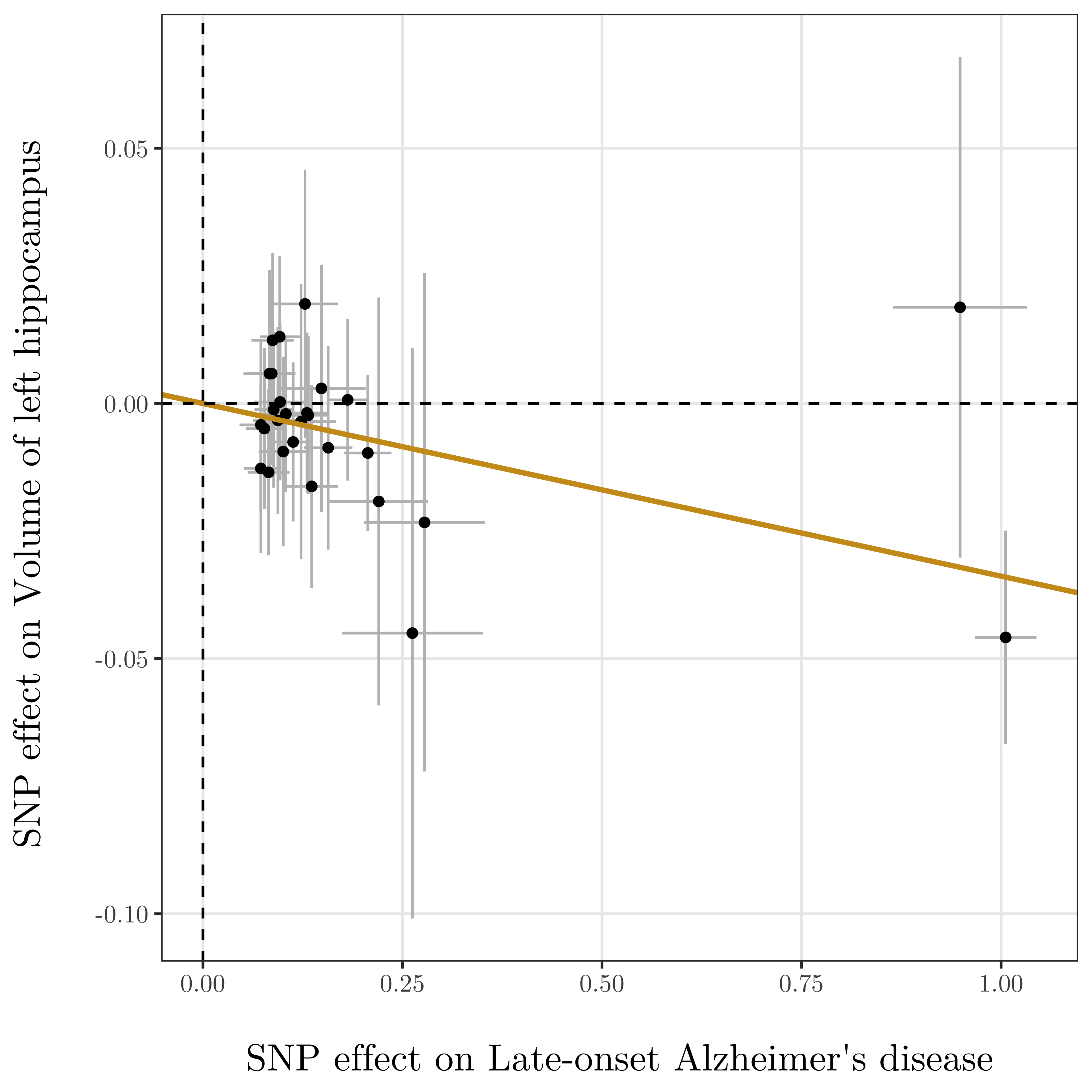 | 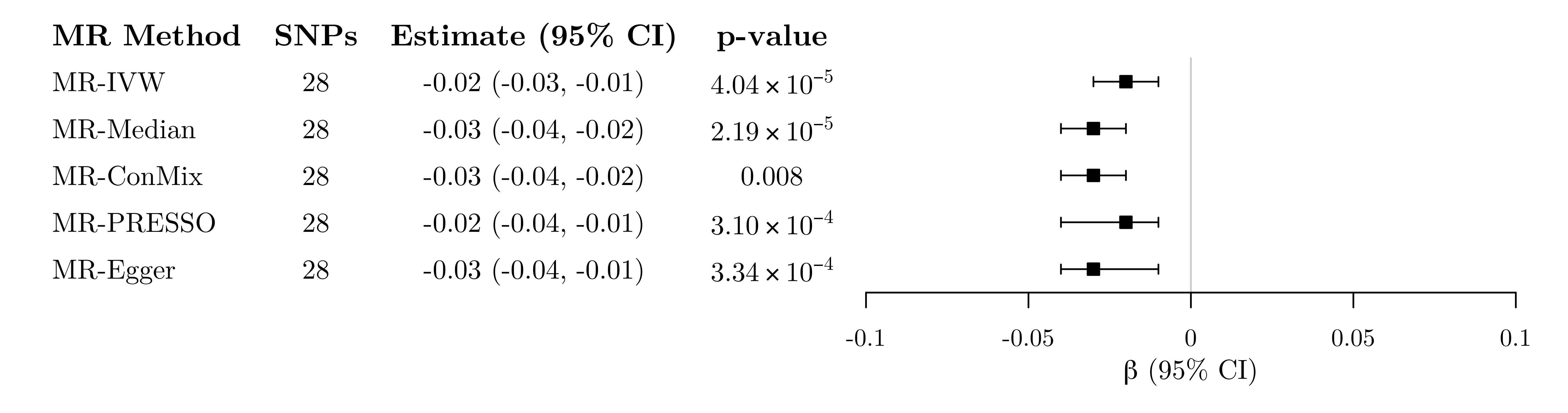 |
| 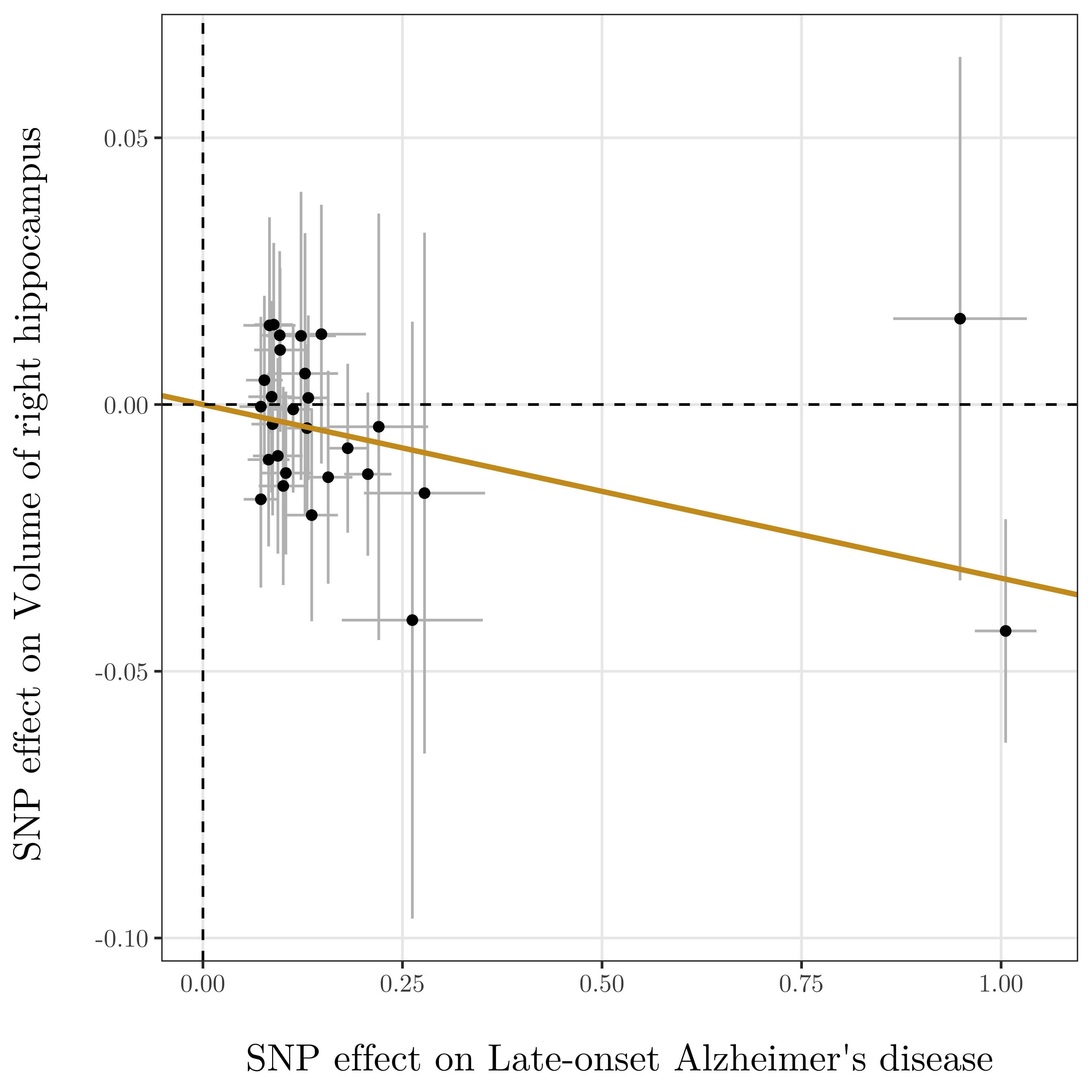 | 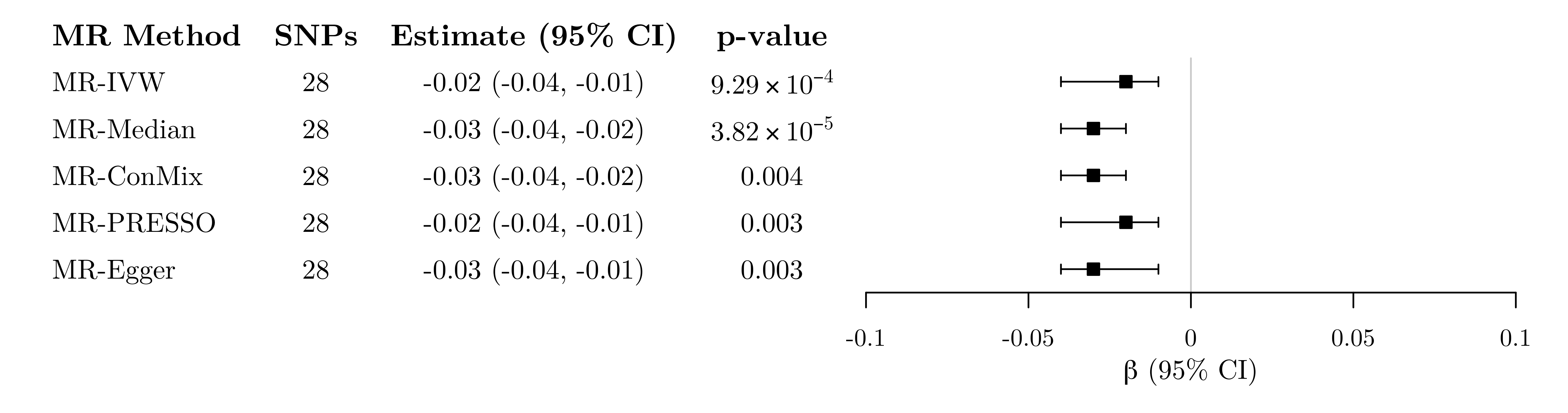 |

**Supplementary Fig. 26** Left: Genetic associations with late-onset Alzheimer’s disease (horizontal axis, log odds ratios) and with four imaging-derived phenotypes (vertical axis, standard deviation units) for 28 genetic variants associated with late-onset Alzheimer’s disease at a genome-wide level of significance. Horizontal and vertical lines represent 95% confidence intervals for the genetic associations. The regression line through the origin represents the inverse-variance weighted Mendelian randomization estimate for the effect of late-onset Alzheimer’s disease on each imaging-derived phenotype. Right: Mendelian randomization estimates of the association between genetically-proxied late-onset Alzheimer’s disease and brain structure phenotypes. Estimates represent the average change in each imaging-derived phenotype (standard deviation units) per doubling (2-fold increase) in the odds of genetically-predicted late-onset AD.

**
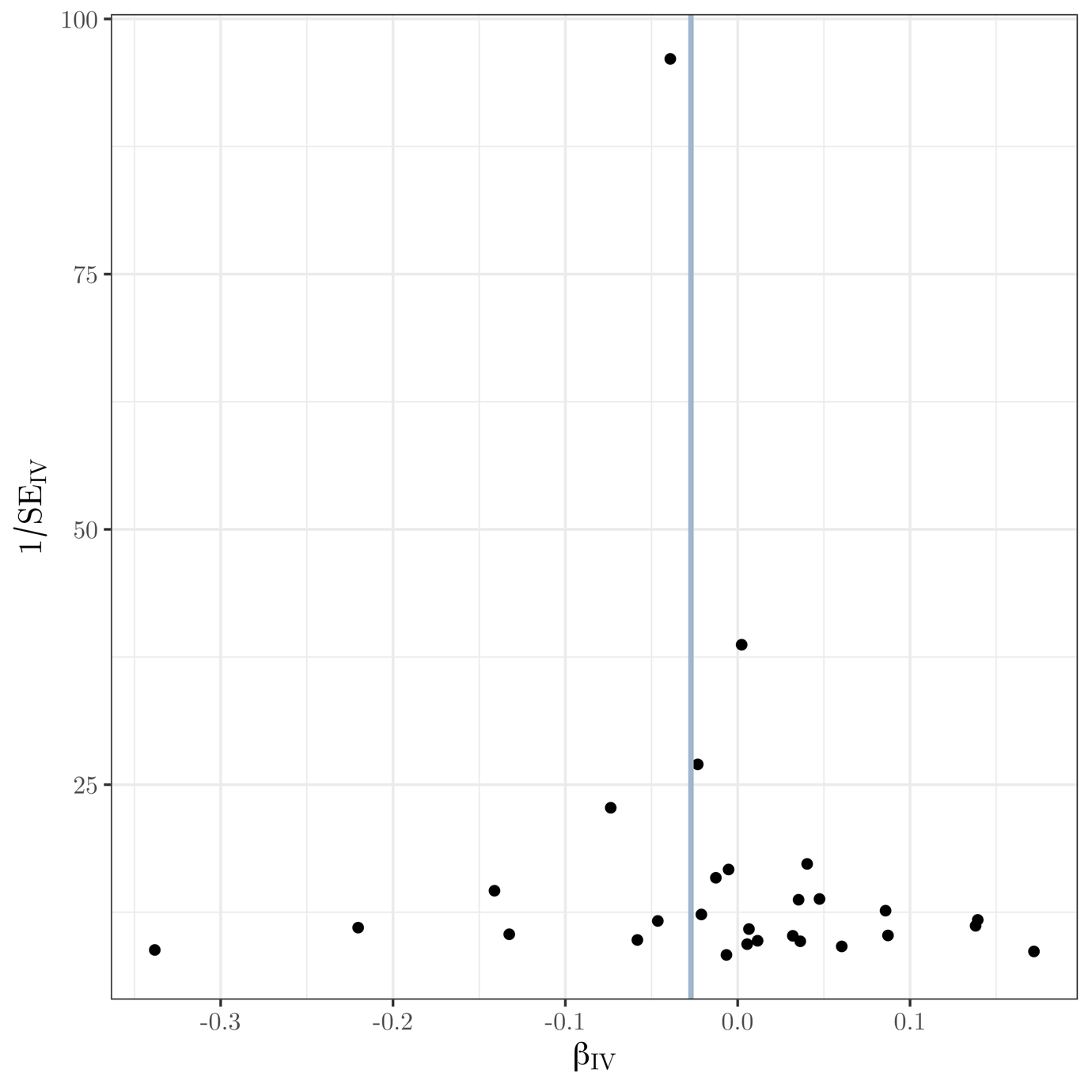
**

**Supplementary Fig. 27** Funnel plot of instrument precision against instrumental variable estimates for each genetic variant separately for Mendelian randomization analysis of late-onset AD on orientation dispersion index. Solid vertical line is the (fixed-effect) inverse-variance weighted estimate.

**
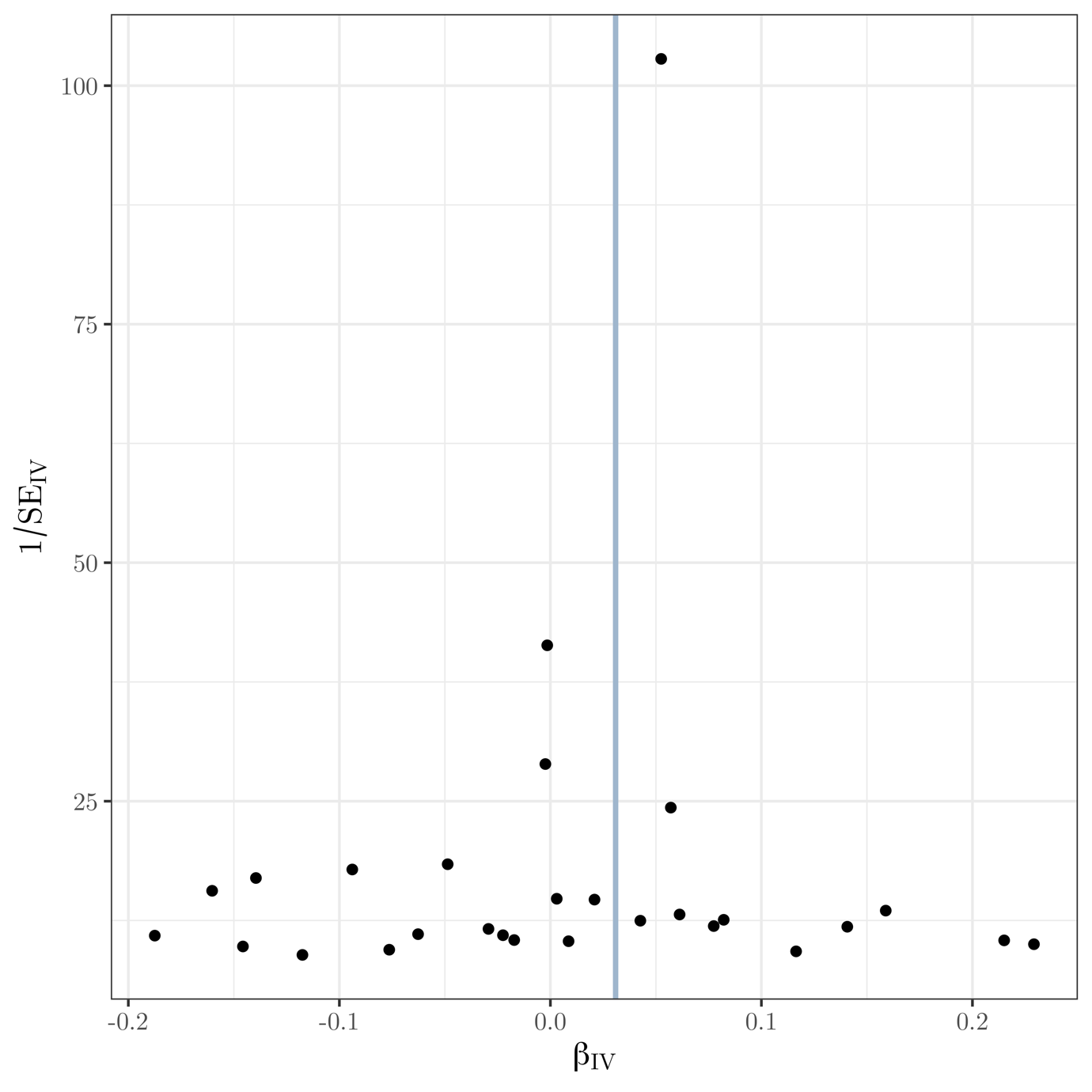
**

**Supplementary Fig. 28** Funnel plot of instrument precision against instrumental variable estimates for each genetic variant separately for Mendelian randomization analysis of late-onset AD on mean diffusivity (white matter tracts). Solid vertical line is the (fixed-effect) inverse-variance weighted estimate.

**
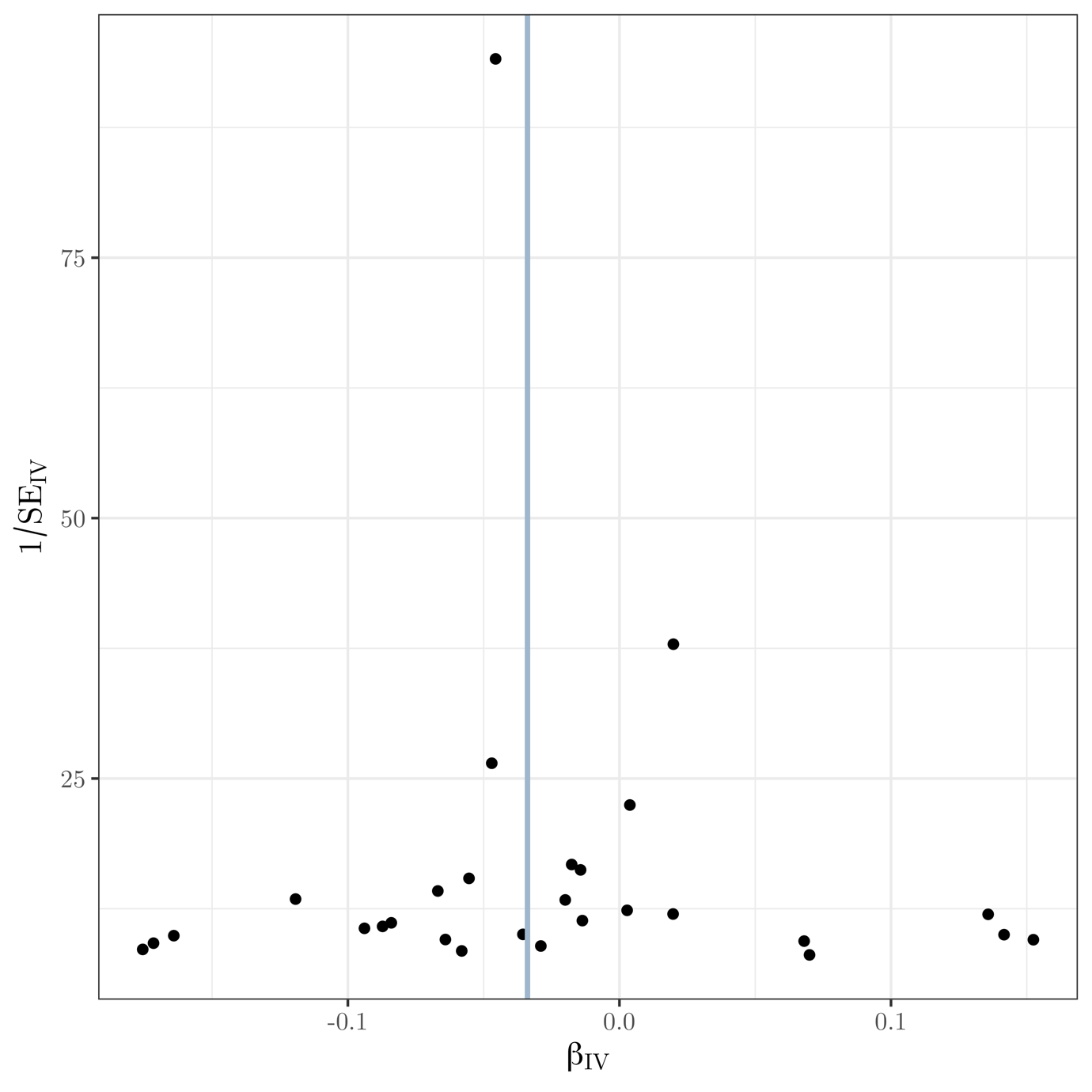
**

**Supplementary Fig. 29** Funnel plot of instrument precision against instrumental variable estimates for each genetic variant separately for Mendelian randomization analysis of late-onset AD on volume of left hippocampus. Solid vertical line is the (fixed-effect) inverse-variance weighted estimate.

**
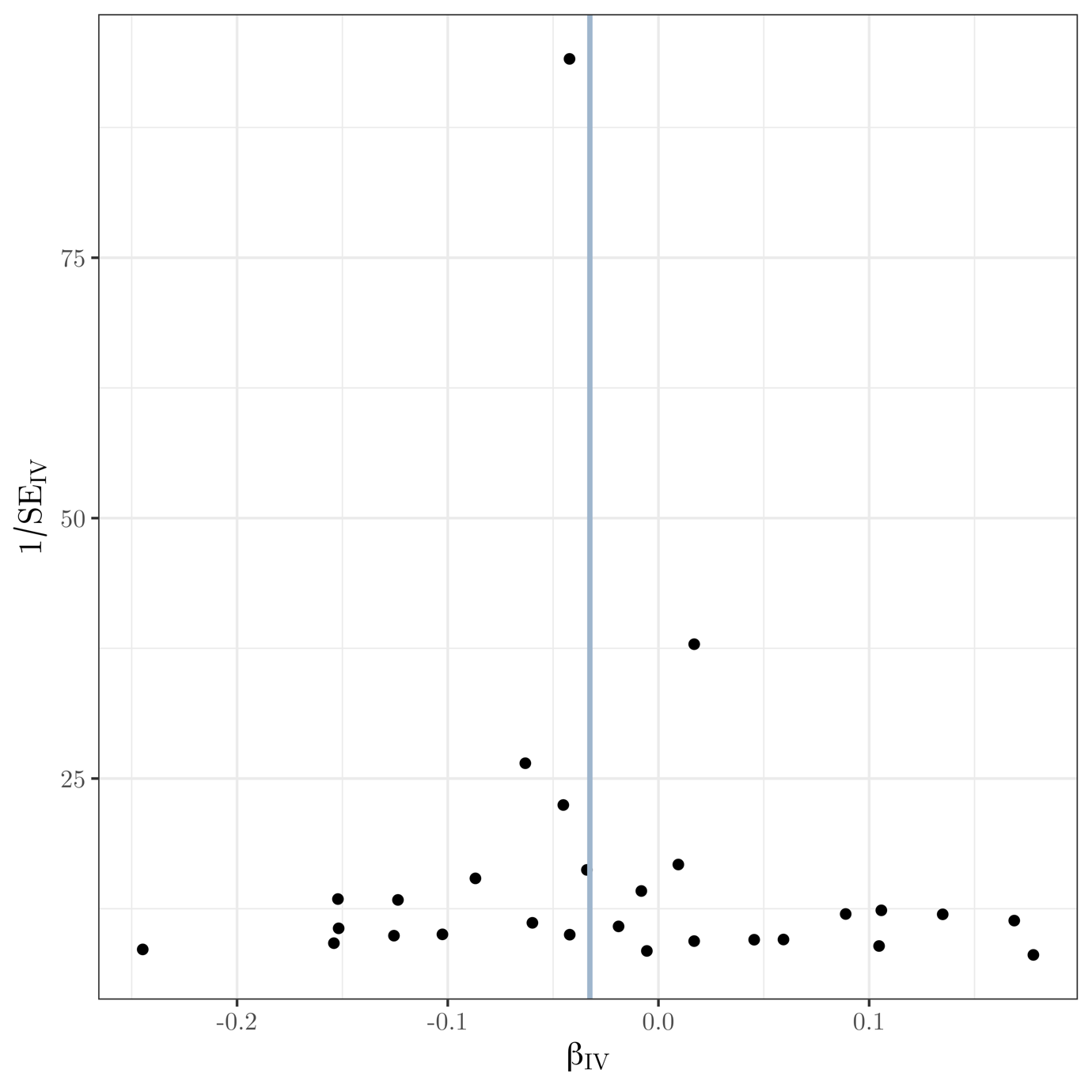
**

**Supplementary Fig. 30** Funnel plot of instrument precision against instrumental variable estimates for each genetic variant separately for Mendelian randomization analysis of late-onset AD on volume of right hippocampus. Solid vertical line is the (fixed-effect) inverse-variance weighted estimate.

| 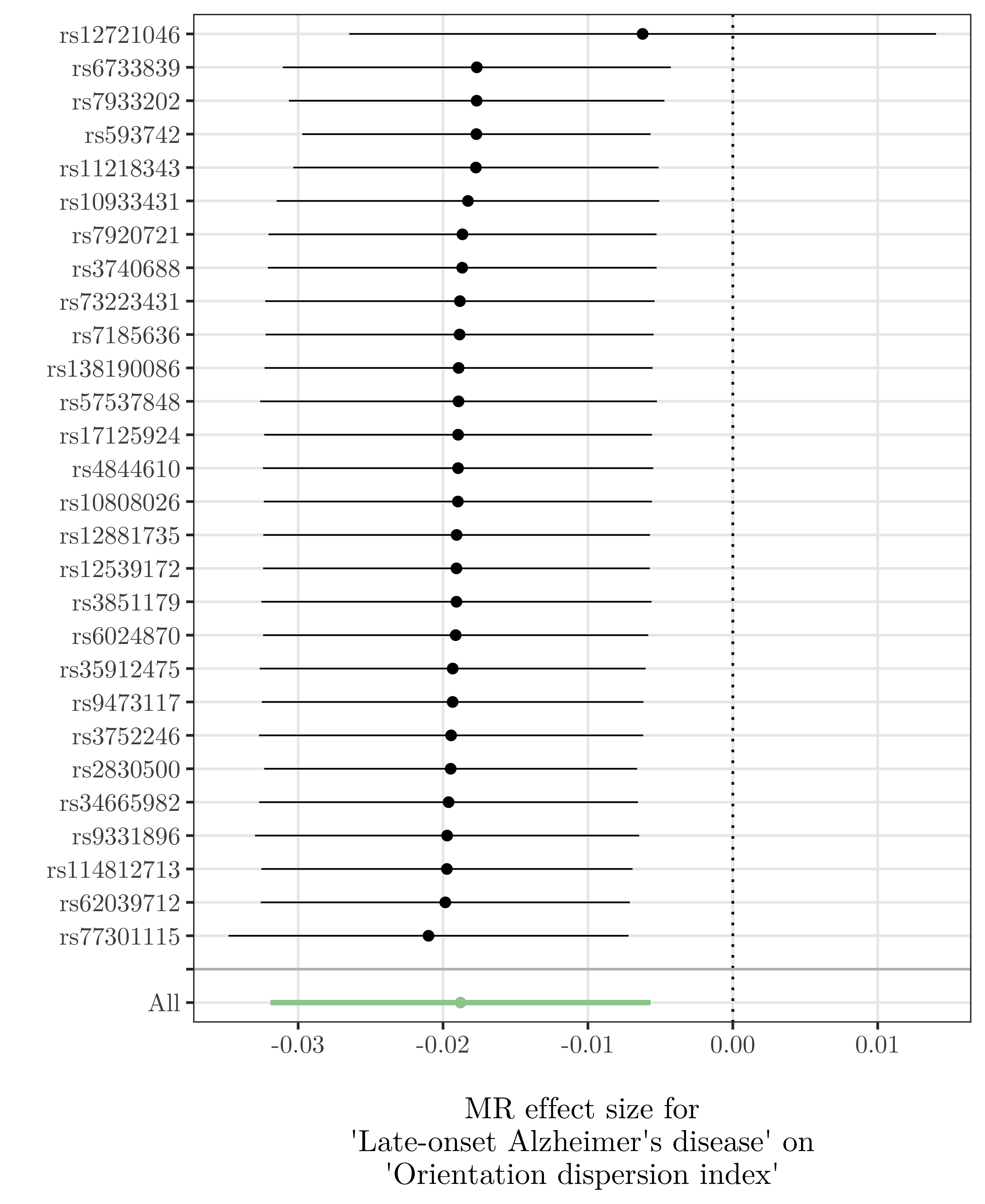 | 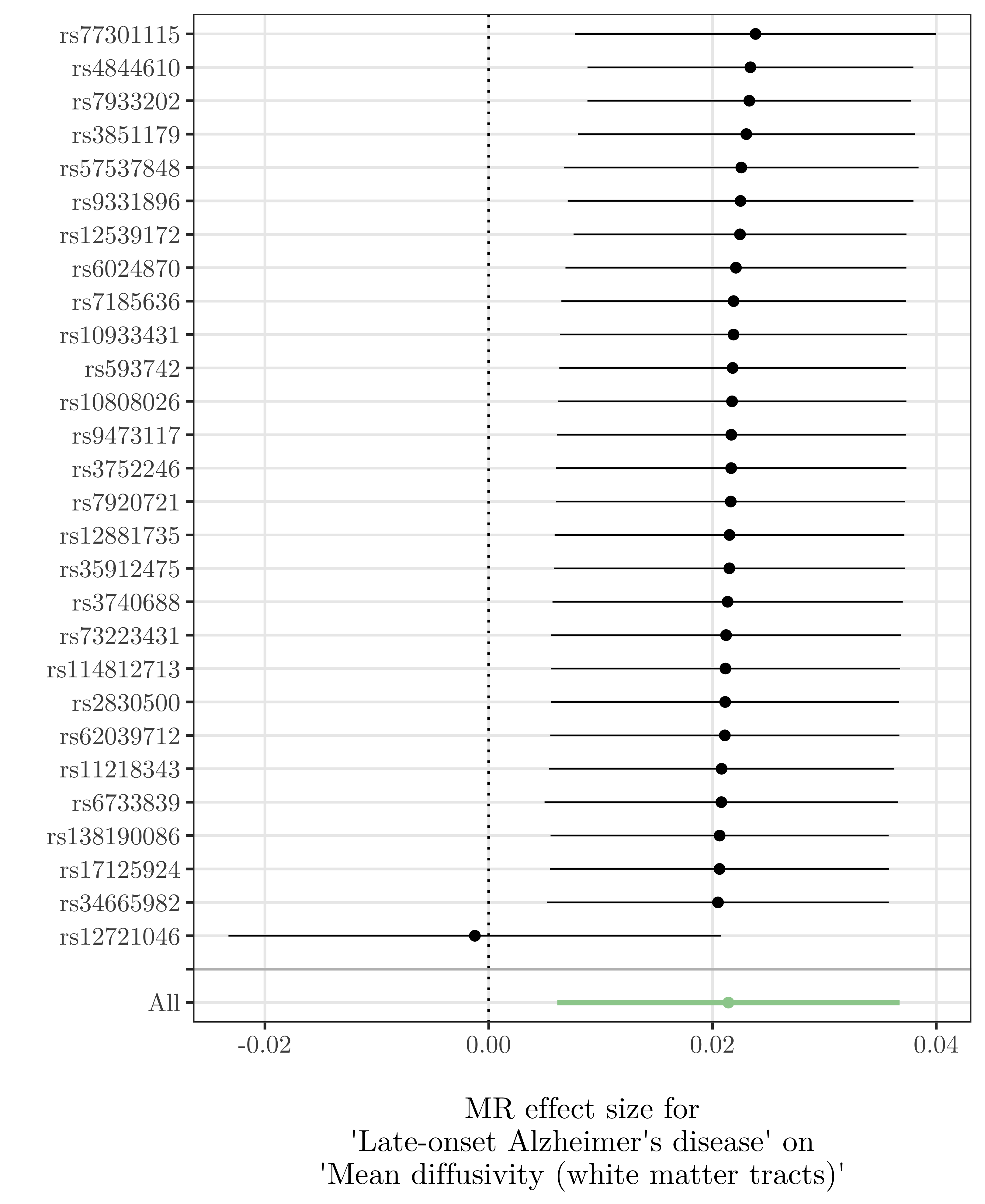 |
| --- | --- |
| 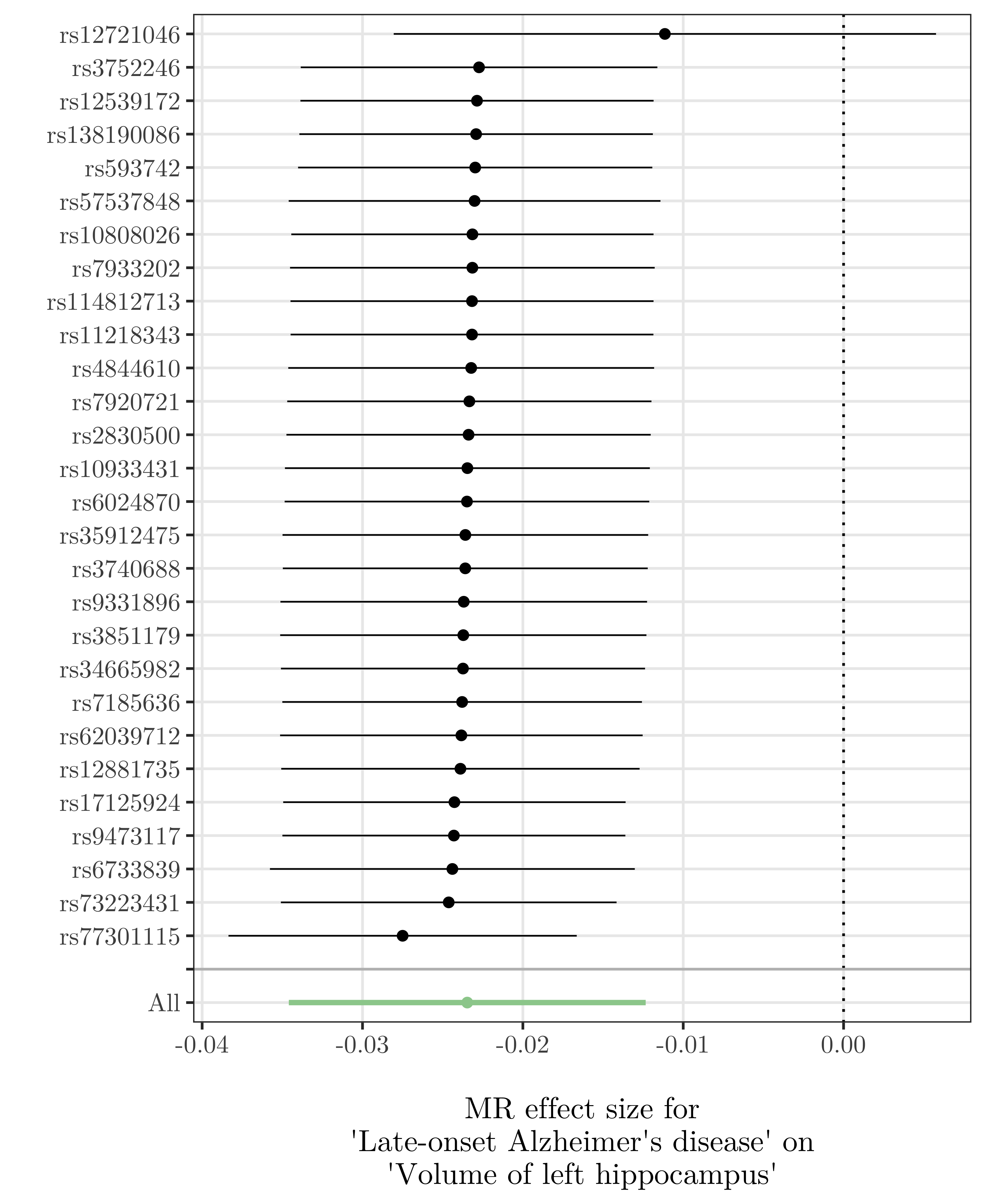 | 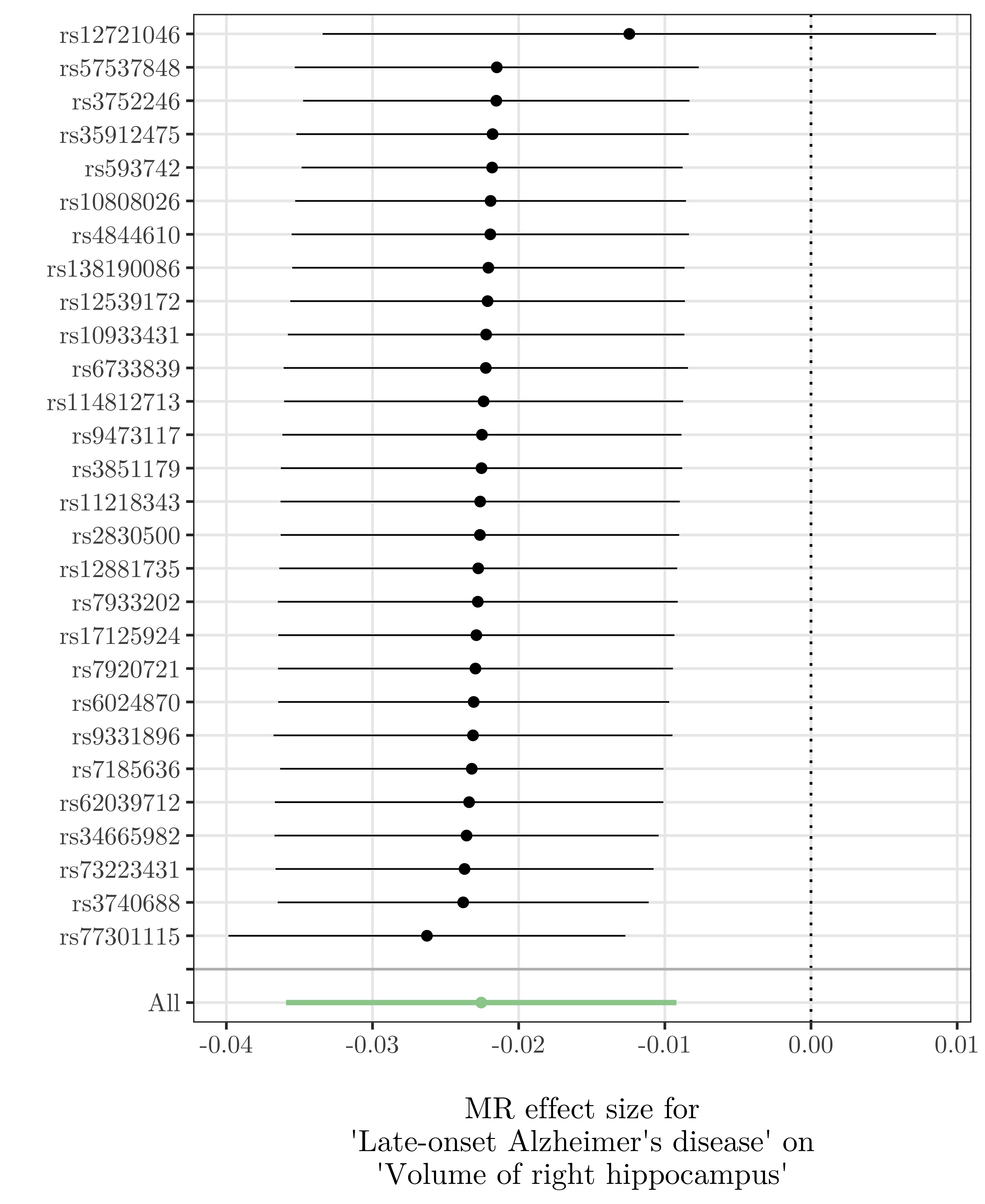 |

**Supplementary Fig. 31 Leave-one-out plots for the analysis of late-onset Alzheimer’s disease on four imaging-derived phenotypes.** Point estimates represent the inverse-variance weighted (IVW) estimate when excluding each SNP in turn. Horizontal lines represent 95% confidence intervals around the IVW estimate.

| 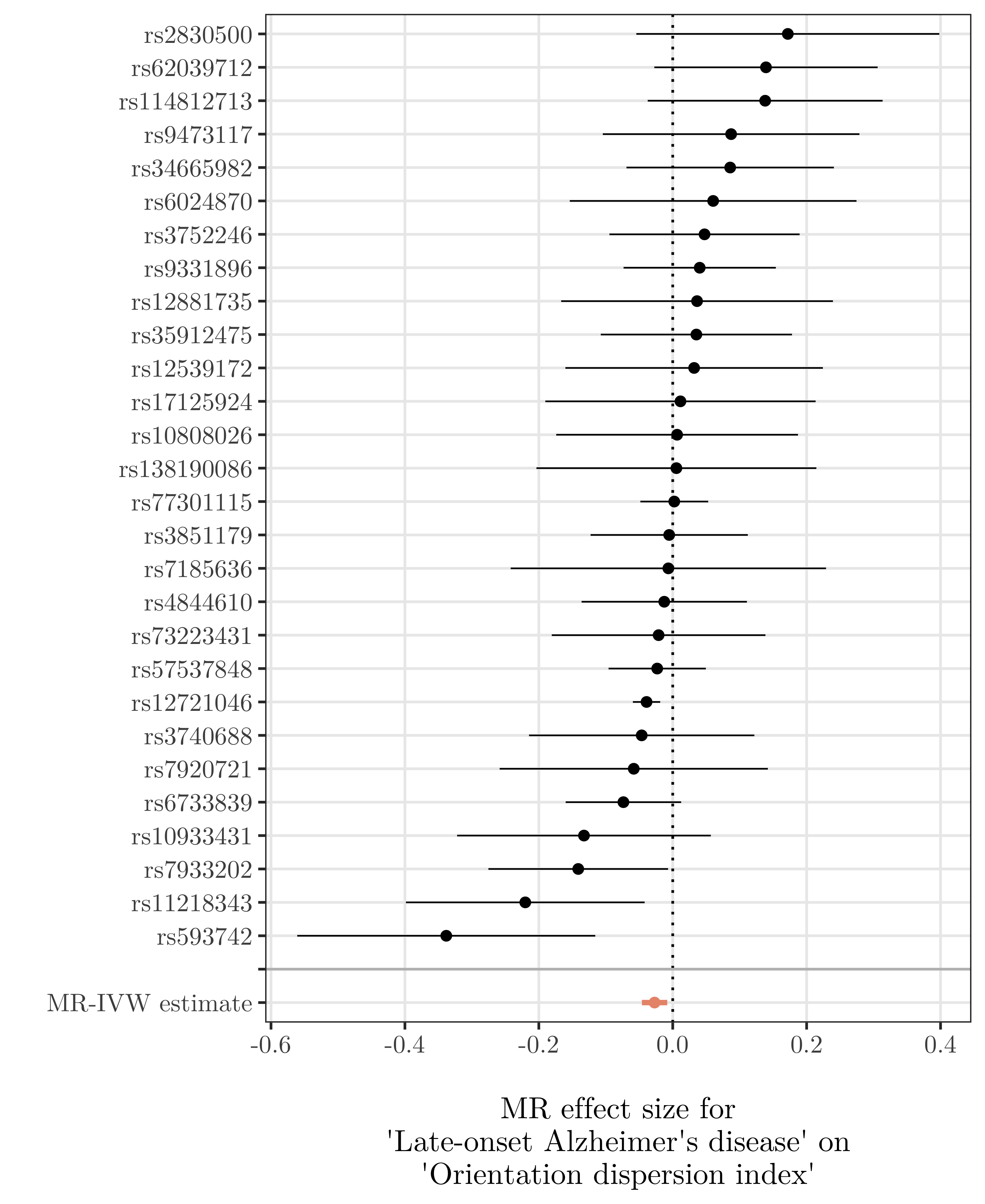 | 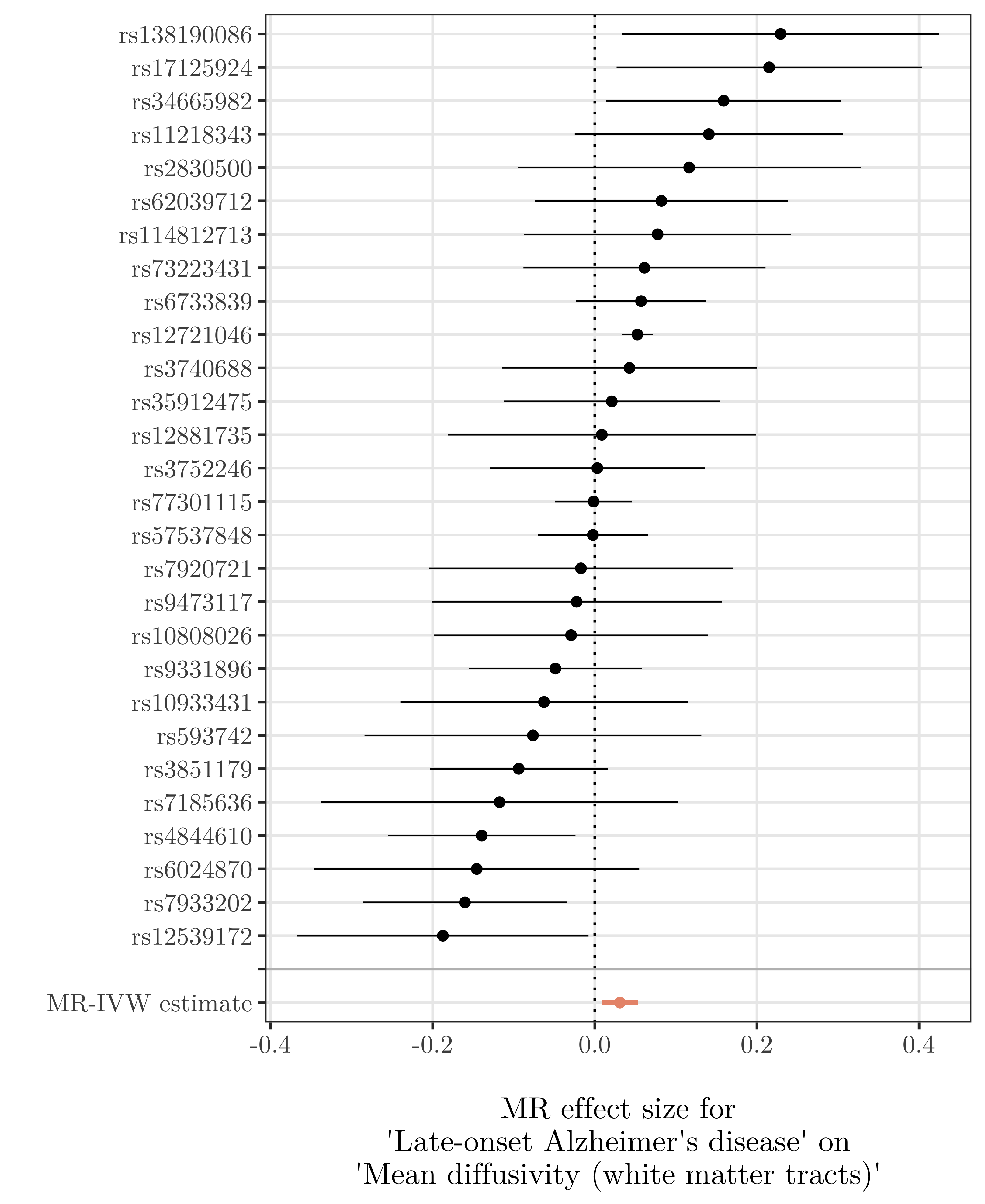 |
| --- | --- |
| 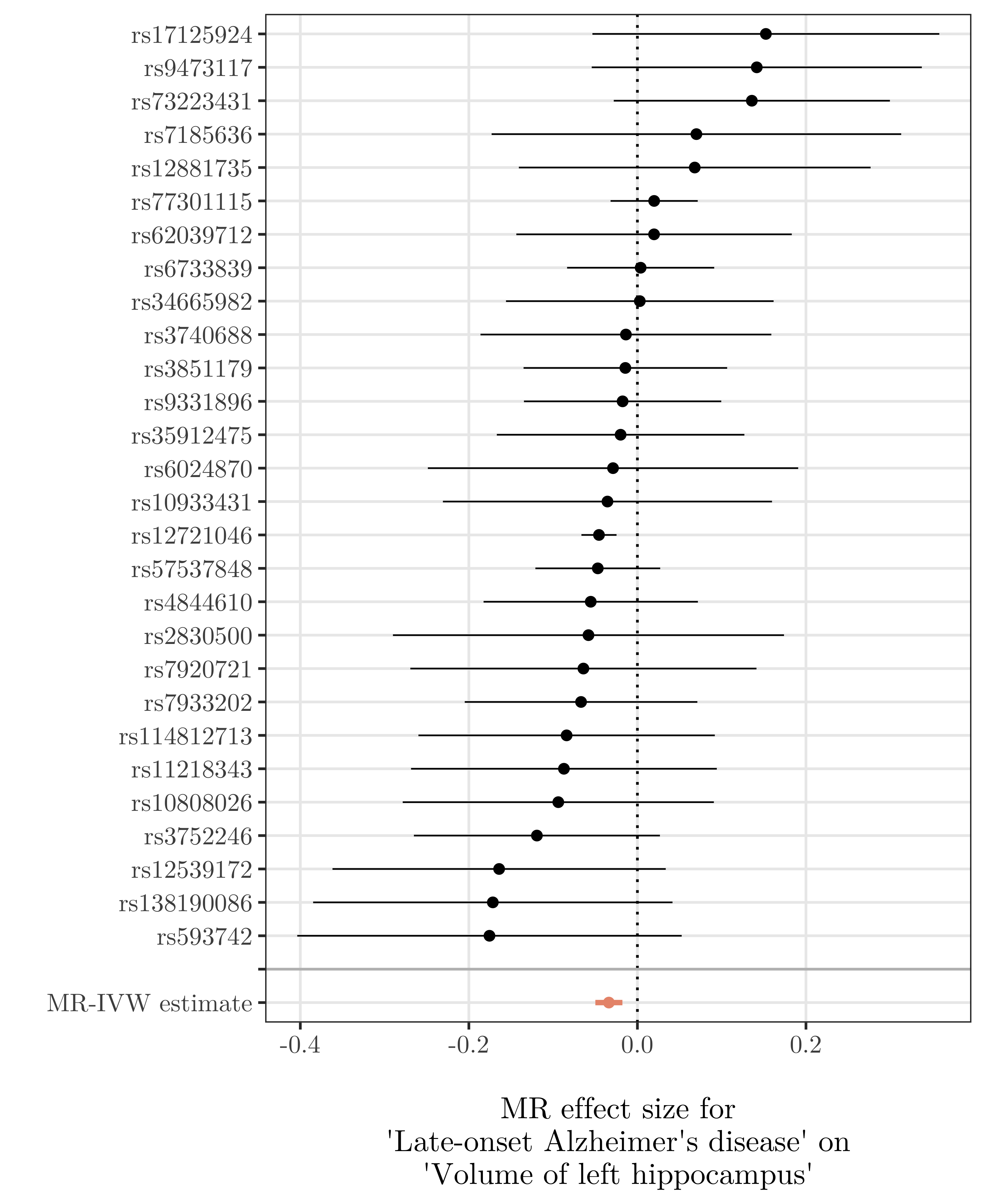 | 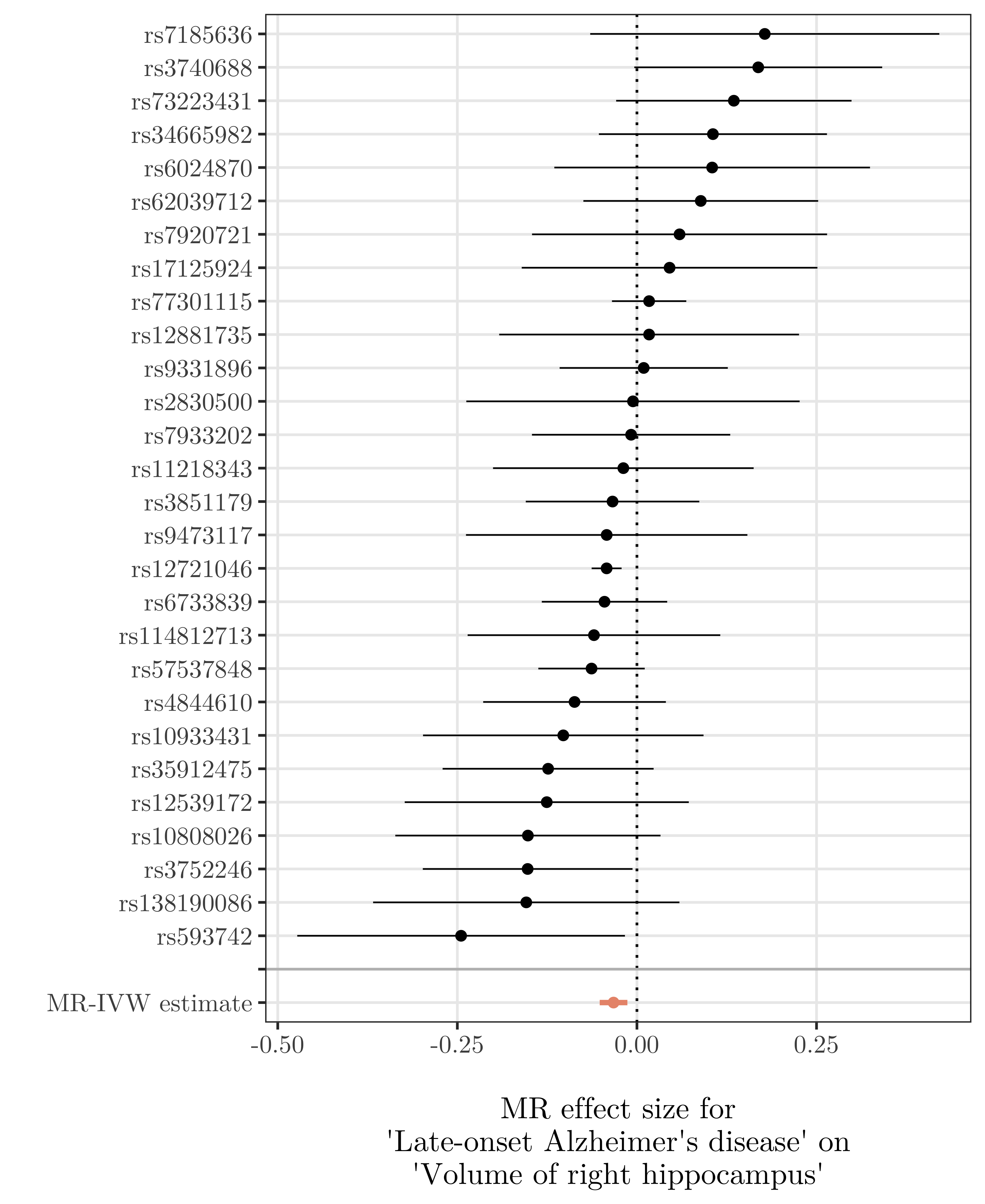 |

**Supplementary Fig. 32 Single-SNP analysis forest plots of the effect of late-onset Alzheimer’s disease on four imaging-derived phenotypes**. Point estimates represent the variant-specific ratio estimates for each SNP (in black), and the inverse-variance weighted (IVW) estimate (in orange). Horizontal lines represent 95% confidence intervals around the variant-specific ratio estimates and the IVW estimate.

## **References**

1. Ronan L, Voets N, Rua C, Alexander-Bloch A, Hough M, Mackay C, et al. Differential Tangential Expansion as a Mechanism for Cortical Gyrification. Cereb Cortex. 2014;24(8):2219–28.

2. Ronan L, Voets NL, Hough M, Mackay C, Roberts N, Suckling J, et al. Consistency and interpretation of changes in millimeter-scale cortical intrinsic curvature across three independent datasets in schizophrenia. Neuroimage. 2012;63(1):611–21.

3. Pillay P, Manger PR. Order‐specific quantitative patterns of cortical gyrification. Eur J Neurosci. 2007;25(9):2705–12.

4. Schaer M, Cuadra MB, Tamarit L, Lazeyras F, Eliez S, Thiran JP. A Surface-Based Approach to Quantify Local Cortical Gyrification. Ieee T Med Imaging. 2008;27(2):161–70.

5. RONAN L, PIENAAR R, WILLIA G, BULLMORE E, CROW TJ, ROBERTS N, et al. INTRINSIC CURVATURE: A MARKER OF MILLIMETER-SCALE TANGENTIAL CORTICO-CORTICAL CONNECTIVITY? Int J Neural Syst. 2011;21(05):351–66.

6. Basser PJ, Mattiello J, Lebihan D. Estimation of the Effective Self-Diffusion Tensor from the NMR Spin Echo. J Magnetic Reson Ser B. 1994;103(3):247–54.

7. Clark KA, Nuechterlein KH, Asarnow RF, Hamilton LS, Phillips OR, Hageman NS, et al. Mean diffusivity and fractional anisotropy as indicators of disease and genetic liability to schizophrenia. J Psychiatr Res. 2011;45(7):980–8.

8. Zhao B, Li T, Yang Y, Wang X, Luo T, Shan Y, et al. Common genetic variation influencing human white matter microstructure. Science. 2021;372(6548):eabf3736.

9. Pierpaoli C, Jezzard P, Basser PJ, Barnett A, Chiro GD. Diffusion tensor MR imaging of the human brain. Radiology. 1996;201(3):637–48.

10. Zhang H, Schneider T, Wheeler-Kingshott CA, Alexander DC. NODDI: Practical in vivo neurite orientation dispersion and density imaging of the human brain. Neuroimage. 2012;61(4):1000–16.

11. Fukutomi H, Glasser MF, Zhang H, Autio JA, Coalson TS, Okada T, et al. Neurite imaging reveals microstructural variations in human cerebral cortical gray matter. Neuroimage. 2018;182:488–99.

12. Schroeter ML, Stein T, Maslowski N, Neumann J. Neural correlates of Alzheimer’s disease and mild cognitive impairment: A systematic and quantitative meta-analysis involving 1351 patients. Neuroimage. 2009;47(4):1196–206.

13. Yang J, Pan P, Song W, Huang R, Li J, Chen K, et al. Voxelwise meta-analysis of gray matter anomalies in Alzheimer’s disease and mild cognitive impairment using anatomic likelihood estimation. J Neurol Sci. 2012;316(1–2):21–9.

14. Mak E, Su L, Williams GB, Watson R, Firbank MJ, Blamire AM, et al. Progressive cortical thinning and subcortical atrophy in dementia with Lewy bodies and Alzheimer’s disease. Neurobiol Aging. 2015;36(4):1743–50.

15. Singh V, Chertkow H, Lerch JP, Evans AC, Dorr AE, Kabani NJ. Spatial patterns of cortical thinning in mild cognitive impairment and Alzheimer’s disease. Brain. 2006;129(11):2885–93.

16. Koval I, Bône A, Louis M, Lartigue T, Bottani S, Marcoux A, et al. AD Course Map charts Alzheimer’s disease progression. Sci Rep-uk. 2021;11(1):8020.

17. Krumm S, Kivisaari SL, Probst A, Monsch AU, Reinhardt J, Ulmer S, et al. Cortical thinning of parahippocampal subregions in very early Alzheimer’s disease. Neurobiol Aging. 2016;38:188–96.

18. Yang H, Xu H, Li Q, Jin Y, Jiang W, Wang J, et al. Study of brain morphology change in Alzheimer’s disease and amnestic mild cognitive impairment compared with normal controls. Gen Psychiatry. 2019;32(2):e100005.

19. Im K, Lee JM, Seo SW, Kim SH, Kim SI, Na DL. Sulcal morphology changes and their relationship with cortical thickness and gyral white matter volume in mild cognitive impairment and Alzheimer’s disease. Neuroimage. 2008;43(1):103–13.

20. Zhao H, Shao PF. Altered local gyrification index and corresponding functional connectivity in Alzheimer disease. Alzheimer’s Dementia. 2021;17(S4).

21. Solé-Padullés C, Bartrés-Faz D, Junqué C, Vendrell P, Rami L, Clemente IC, et al. Brain structure and function related to cognitive reserve variables in normal aging, mild cognitive impairment and Alzheimer’s disease. Neurobiol Aging. 2009;30(7):1114–24.

22. Arenaza-Urquijo EM, Landeau B, Joie RL, Mevel K, Mézenge F, Perrotin A, et al. Relationships between years of education and gray matter volume, metabolism and functional connectivity in healthy elders. Neuroimage. 2013;83:450–7.

23. Bartrés-Faz D, Solé-Padullés C, Junqué C, Rami L, Bosch B, Bargalló N, et al. Interactions of cognitive reserve with regional brain anatomy and brain function during a working memory task in healthy elders. Biol Psychol. 2009;80(2):256–9.

24. Kim JP, Seo SW, Shin HY, Ye BS, Yang JJ, Kim C, et al. Effects of education on aging-related cortical thinning among cognitively normal individuals. Neurology. 2015;85(9):806–12.

25. Vaqué-Alcázar L, Sala-Llonch R, Valls-Pedret C, Vidal-Piñeiro D, Fernández-Cabello S, Bargalló N, et al. Differential age-related gray and white matter impact mediates educational influence on elders’ cognition. Brain Imaging Behav. 2017;11(2):318–32.

26. Bartrés-Faz D, Arenaza-Urquijo EM. Structural and Functional Imaging Correlates of Cognitive and Brain Reserve Hypotheses in Healthy and Pathological Aging. Brain Topogr. 2011;24(3–4):340.

27. Nyberg L, Magnussen F, Lundquist A, Baaré W, Bartrés-Faz D, Bertram L, et al. Educational attainment does not influence brain aging. Proc National Acad Sci. 2021;118(18):e2101644118.

28. Consortium A, Liu Y, Julkunen V, Paajanen T, Westman E, Wahlund LO, et al. Education increases reserve against Alzheimer’s disease—evidence from structural MRI analysis. Neuroradiology. 2012;54(9):929–38.

29. Tang X, Varma VR, Miller MI, Carlson MC. Education is associated with sub-regions of the hippocampus and the amygdala vulnerable to neuropathologies of Alzheimer’s disease. Brain Struct Funct. 2017;222(3):1469–79.

30. Vogt NM, Hunt JF, Adluru N, Dean DC, Johnson SC, Asthana S, et al. Cortical Microstructural Alterations in Mild Cognitive Impairment and Alzheimer’s Disease Dementia. Cereb Cortex. 2020;30(5):2948–60.

31. Nazeri A, Schifani C, Anderson JAE, Ameis SH, Voineskos AN. In Vivo Imaging of Gray Matter Microstructure in Major Psychiatric Disorders: Opportunities for Clinical Translation. Biological Psychiatry Cognitive Neurosci Neuroimaging. 2020;5(9):855–64.

32. Weston PSJ, Simpson IJA, Ryan NS, Ourselin S, Fox NC. Diffusion imaging changes in grey matter in Alzheimer’s disease: a potential marker of early neurodegeneration. Alzheimer’s Res Ther. 2015;7(1):47.

33. Jacobs HIL, Boxtel MPJ, Gronenschild EHBM, Uylings HBM, Jolles J, Verhey FRJ. Decreased gray matter diffusivity: A potential early Alzheimer’s disease biomarker? Alzheimer’s Dementia. 2013;9(1):93–7.

34. Müller MJ, Greverus D, Dellani PR, Weibrich C, Wille PR, Scheurich A, et al. Functional implications of hippocampal volume and diffusivity in mild cognitive impairment. Neuroimage. 2005;28(4):1033–42.

35. Kantarci K, Petersen RC, Boeve BF, Knopman DS, Weigand SD, O’Brien PC, et al. DWI predicts future progression to Alzheimer disease in amnestic mild cognitive impairment. Neurology. 2005;64(5):902–4.

36. Bozzali M, Falini A, Franceschi M, Cercignani M, Zuffi M, Scotti G, et al. White matter damage in Alzheimer’s disease assessed in vivo using diffusion tensor magnetic resonance imaging. J Neurology Neurosurg Psychiatry. 2002;72(6):742.

37. Naggara O, Oppenheim C, Rieu D, Raoux N, Rodrigo S, Barba GD, et al. Diffusion tensor imaging in early Alzheimer’s disease. Psychiatry Res Neuroimaging. 2006;146(3):243–9.

38. Stahl R, Dietrich O, Teipel SJ, Hampel H, Reiser MF, Schoenberg SO. White Matter Damage in Alzheimer Disease and Mild Cognitive Impairment: Assessment with Diffusion-Tensor MR Imaging and Parallel Imaging Techniques. Radiology. 2007;243(2):483–92.

39. Takahashi S, Yonezawa H, Takahashi J, Kudo M, Inoue T, Tohgi H. Selective reduction of diffusion anisotropy in white matter of Alzheimer disease brains measured by 3.0 Tesla magnetic resonance imaging. Neurosci Lett. 2002;332(1):45–8.

40. Teipel SJ, Meindl T, Wagner M, Kohl T, Bürger K, Reiser MF, et al. White Matter Microstructure in Relation to Education in Aging and Alzheimer’s Disease1. J Alzheimer’s Dis. 2009;17(3):571–83.

41. Jack CR, Knopman DS, Jagust WJ, Shaw LM, Aisen PS, Weiner MW, et al. Hypothetical model of dynamic biomarkers of the Alzheimer’s pathological cascade. Lancet Neurology. 2010;9(1):119–28.

42. Villemagne VL, Burnham S, Bourgeat P, Brown B, Ellis KA, Salvado O, et al. Amyloid β deposition, neurodegeneration, and cognitive decline in sporadic Alzheimer’s disease: a prospective cohort study. Lancet Neurology. 2013;12(4):357–67.

43. Miller MI, Younes L, Ratnanather JT, Brown T, Trinh H, Lee DS, et al. Amygdalar atrophy in symptomatic Alzheimer’s disease based on diffeomorphometry: the BIOCARD cohort. Neurobiol Aging. 2015;36:S3–10.

44. Tang X, Holland D, Dale AM, Younes L, Miller MI, Initiative for the ADN. Shape abnormalities of subcortical and ventricular structures in mild cognitive impairment and Alzheimer’s disease: Detecting, quantifying, and predicting. Hum Brain Mapp. 2014;35(8):3701–25.

45. Salthouse TA. Neuroanatomical Substrates of Age-Related Cognitive Decline. Psychol Bull. 2011;137(5):753–84.

46. Garde E, Mortensen EL, Krabbe K, Rostrup E, Larsson HB. Relation between age-related decline in intelligence and cerebral white-matter hyperintensities in healthy octogenarians: a longitudinal study. Lancet. 2000;356(9230):628–34.

47. Maillard P, Carmichael O, Fletcher E, Reed B, Mungas D, DeCarli C. Coevolution of white matter hyperintensities and cognition in the elderly. Neurology. 2012;79(5):442–8.

48. Moroni F, Ammirati E, Rocca MA, Filippi M, Magnoni M, Camici PG. Cardiovascular disease and brain health: Focus on white matter hyperintensities. Int J Cardiol Hear Vasc. 2018;19:63–9.

49. Lee JJ, Wedow R, Okbay A, Kong E, Maghzian O, Zacher M, et al. Gene discovery and polygenic prediction from a genome-wide association study of educational attainment in 1.1 million individuals. Nat Genet. 2018;50(8):1112–21.

50. Kunkle BW, Grenier-Boley B, Sims R, Bis JC, Damotte V, Naj AC, et al. Genetic meta-analysis of diagnosed Alzheimer’s disease identifies new risk loci and implicates Aβ, tau, immunity and lipid processing. Nat Genet. 2019;51(3):414–30.

51. Lambert JC, Ibrahim-Verbaas CA, Harold D, Naj AC, Sims R, Bellenguez C, et al. Meta-analysis of 74,046 individuals identifies 11 new susceptibility loci for Alzheimer’s disease. Nat Genet. 2013;45(12):1452–8.

52. Sudlow C, Gallacher J, Allen N, Beral V, Burton P, Danesh J, et al. UK Biobank: An Open Access Resource for Identifying the Causes of a Wide Range of Complex Diseases of Middle and Old Age. Plos Med. 2015;12(3):e1001779.

53. Miller KL, Alfaro-Almagro F, Bangerter NK, Thomas DL, Yacoub E, Xu J, et al. Multimodal population brain imaging in the UK Biobank prospective epidemiological study. Nat Neurosci. 2016;19(11):1523–36.

54. Warrier V, Stauffer EM, Huang QQ, Wigdor EM, Slob EAW, Seidlitz J, et al. The genetics of cortical organisation and development: a study of 2,347 neuroimaging phenotypes. Biorxiv. 2022;2022.09.08.507084.

55. Fischl B, Kouwe A van der, Destrieux C, Halgren E, Ségonne F, Salat DH, et al. Automatically Parcellating the Human Cerebral Cortex. Cereb Cortex. 2004;14(1):11–22.

56. Daducci A, Canales-Rodríguez EJ, Zhang H, Dyrby TB, Alexander DC, Thiran JP. Accelerated Microstructure Imaging via Convex Optimization (AMICO) from diffusion MRI data. Neuroimage. 2015;105:32–44.

57. Rosen AFG, Roalf DR, Ruparel K, Blake J, Seelaus K, Villa LP, et al. Quantitative assessment of structural image quality. Neuroimage. 2018;169:407–18.

58. Jahanshad N, Kochunov PV, Sprooten E, Mandl RC, Nichols TE, Almasy L, et al. Multi-site genetic analysis of diffusion images and voxelwise heritability analysis: A pilot project of the ENIGMA–DTI working group. Neuroimage. 2013;81:455–69.

59. Kochunov P, Jahanshad N, Sprooten E, Nichols TE, Mandl RC, Almasy L, et al. Multi-site study of additive genetic effects on fractional anisotropy of cerebral white matter: Comparing meta and megaanalytical approaches for data pooling. Neuroimage. 2014;95:136–50.

60. Smith SM, Douaud G, Chen W, Hanayik T, Alfaro-Almagro F, Sharp K, et al. An expanded set of genome-wide association studies of brain imaging phenotypes in UK Biobank. Nat Neurosci. 2021;1–9.

61. Alfaro-Almagro F, Jenkinson M, Bangerter NK, Andersson JLR, Griffanti L, Douaud G, et al. Image processing and Quality Control for the first 10,000 brain imaging datasets from UK Biobank. Neuroimage. 2018;166:400–24.

62. Marchini J, Howie B. Genotype imputation for genome-wide association studies. Nat Rev Genet. 2010;11(7):499–511.

63. Slob EAW, Burgess S. A comparison of robust Mendelian randomization methods using summary data. Genet Epidemiol. 2020;44(4):313–29.

64. Bowden J, Smith GD, Haycock PC, Burgess S. Consistent Estimation in Mendelian Randomization with Some Invalid Instruments Using a Weighted Median Estimator. Genet Epidemiol. 2016;40(4):304–14.

65. Burgess S, Thompson SG. Mendelian Randomization: Methods for Causal Inference Using Genetic Variants. 2nd ed. CRC Press, Taylor & Francis Group; 2021.

66. Burgess S, Foley CN, Allara E, Staley JR, Howson JMM. A robust and efficient method for Mendelian randomization with hundreds of genetic variants. Nat Commun. 2020;11(1):376.

67. Bowden J, Smith GD, Burgess S. Mendelian randomization with invalid instruments: effect estimation and bias detection through Egger regression. Int J Epidemiol. 2015;44(2):512–25.

68. Burgess S, Thompson SG. Interpreting findings from Mendelian randomization using the MR-Egger method. Eur J Epidemiol. 2017;32(5):377–89.

69. Burgess S. Sample size and power calculations in Mendelian randomization with a single instrumental variable and a binary outcome. Int J Epidemiol. 2014;43(3):922–9.

70. Bowden J, M FDG, Minelli C, Smith GD, Sheehan NA, Thompson JR. Assessing the suitability of summary data for two-sample Mendelian randomization analyses using MR-Egger regression: the role of the I2 statistic. Int J Epidemiol. 2016;45(6):1961–74.

71. Verbanck M, Chen CY, Neale B, Do R. Detection of widespread horizontal pleiotropy in causal relationships inferred from Mendelian randomization between complex traits and diseases. Nat Genet. 2018;50(5):693–8.

72. Burgess S, Thompson SG. Multivariable Mendelian Randomization: The Use of Pleiotropic Genetic Variants to Estimate Causal Effects. Am J Epidemiol. 2015;181(4):251–60.

73. Brouwer RM, Klein M, Grasby KL, Schnack HG, Jahanshad N, Teeuw J, et al. Genetic variants associated with longitudinal changes in brain structure across the lifespan. Nat Neurosci. 2022;25(4):421–32.

74. Park JH, Wacholder S, Gail MH, Peters U, Jacobs KB, Chanock SJ, et al. Estimation of effect size distribution from genome-wide association studies and implications for future discoveries. Nat Genet. 2010;42(7):570–5.
